# A tailored in vivo CRISPR screen identifies *BAP1* as a potent tumor suppressor of sarcoma

**DOI:** 10.1172/jci.insight.192686

**Published:** 2026-06-30

**Authors:** Jianguo Huang, Xingliang Liu, Warren Floyd, William Haugh, Zhaoyu Sun, Melissa J. Kasiewicz, Yaping Wu, Brian Piening, John T. Welle, Wesley K. Rosales, Venkatesh Rajamanickam, So Young Kim, Eric S. Xu, Lixia Luo, Yan Ma, Rutulkumar Patel, Ziqiang Zhang, Brady Bernard, William L. Redmond, Walter J. Urba, R. Bryan Bell, David G. Kirsch

**Affiliations:** 1 Earle A. Chiles Research Institute, Providence Cancer Institute, Portland, Oregon, USA.; 2 Department of Radiation Oncology, Duke University Medical Center, Durham, North Carolina, USA.; 3 Department of Molecular Genetics and Microbiology, Duke University, Durham, North Carolina, USA.; 4 Department of Respiratory and Critical Care Medicine, Shanghai Pudong Hospital, Fudan University Pudong Medical Center, Pudong Hospital of Fudan University, Shanghai, China.; 5 Department of Pharmacology and Cancer Biology, Duke University Medical Center, Durham, North Carolina, USA.; 6 Radiation Medicine Program, Princess Margaret Cancer Center, University Health Network, Toronto, Ontario, Canada.; 7 Department of Radiation Oncology and; 8 Department of Medical Biophysics, University of Toronto, Toronto, Ontario, Canada.

**Keywords:** Genetics, Oncology, Cancer, Mouse models, Therapeutics

## Abstract

Undifferentiated pleomorphic sarcoma (UPS) is one of the most common adult soft-tissue sarcomas (STSs), yet therapeutic progress remains limited because of the absence of recurrent oncogenic driver mutations. To identify tumor suppressors contributing to UPS pathogenesis, we performed a customized in vivo CRISPR/Cas9 screen in mice. This approach identified BRCA1-associated protein 1 (*BAP1*) as a potent tumor suppressor in STS. Integrative analyses using RNA sequencing, multiplex immunohistochemistry, and flow cytometry revealed that *Bap1*-deficient sarcomas exhibited a markedly immunosuppressive tumor microenvironment. Consistent with these findings, BAP1 protein expression was reduced in human UPS, whereas polo-like kinase 1 (PLK1) expression was elevated. Functional studies demonstrated that PLK1 was required for the growth and survival of *Bap1*-deficient sarcomas. Pharmacologic inhibition of PLK1 with volasertib significantly suppressed tumor growth in both syngeneic and autochthonous mouse models. Moreover, combining PLK1 inhibition with anti–PD-1 therapy enhanced tumor control and improved survival compared with either treatment alone. Together, these results identify PLK1 as a potential therapeutic vulnerability in *BAP1*-deficient sarcomas and support further evaluation of combined PLK1 inhibition and immune checkpoint blockade as a treatment strategy for a subset of STSs.

## Abstract 2

<style type=”text/css”>a { text-decoration: none; color: #464feb; } tr th, tr td { border: 1px solid #e6e6e6; } tr th { background-color: #f5f5f5; } </style> A CRISPR screen in mice reveals BAP1 loss drives aggressive sarcoma and immune suppression, identifying PLK1 inhibition plus immunotherapy as a promising treatment strategy.

## Graphical abstract

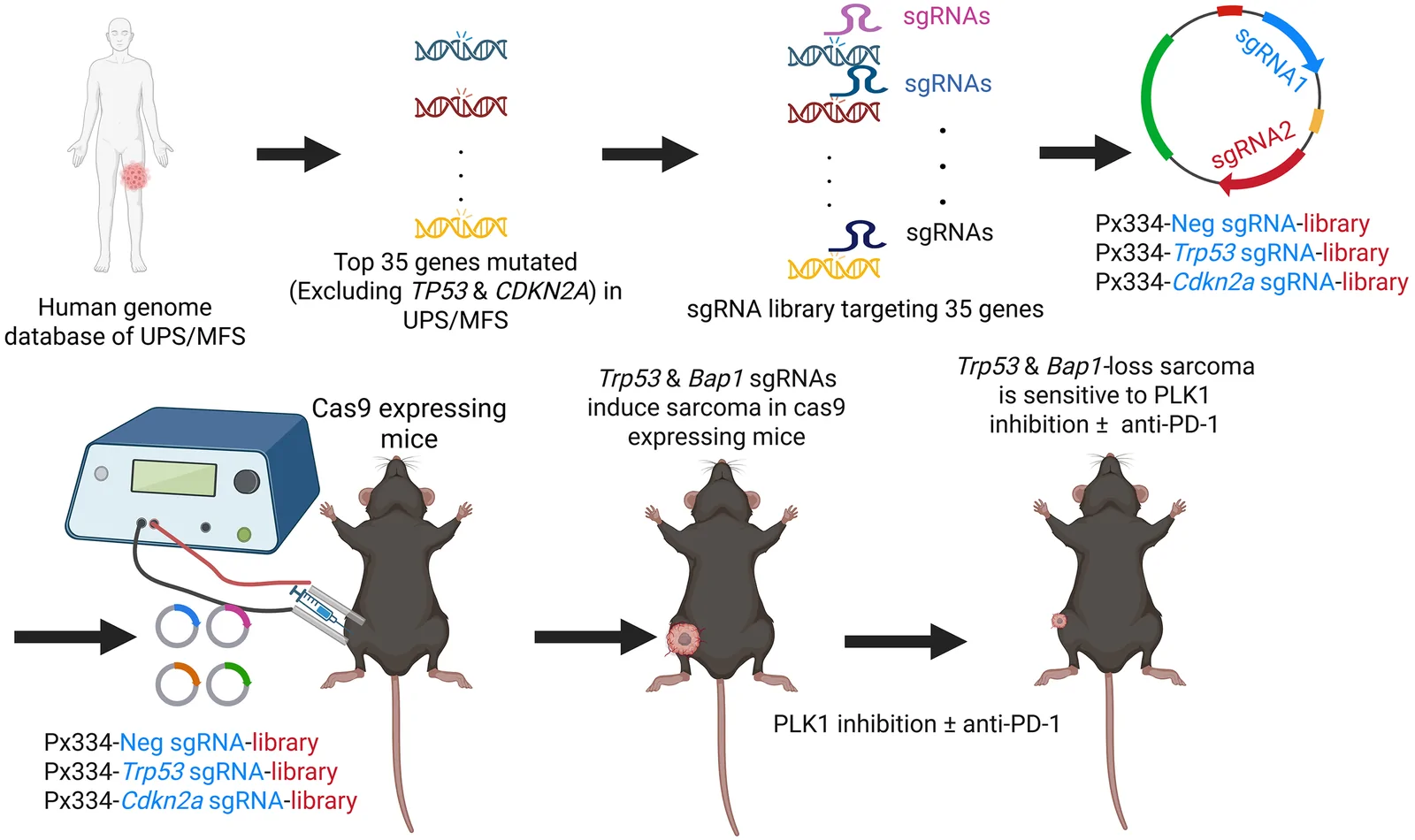

## Introduction

Soft-tissue sarcomas (STSs) are uncommon malignant tumors of mesenchymal origin that arise from various connective tissues (1). More than 75 histological subtypes have been described, creating substantial challenges for the development of subtype-specific therapies. Among these, undifferentiated pleomorphic sarcoma (UPS) is one of the most frequently diagnosed sarcomas in adults. UPS is an aggressive, high-grade STS, with development of pulmonary metastases in nearly half of patients and a median survival of less than 15 months (2, 3). For decades, little therapeutic progress has been made in the treatment of STS. Anthracycline (doxorubicin)-based chemotherapy has remained the standard chemotherapy treatment of high-grade STS, including metastatic UPS, for over 40 years (4). However, unlike other cancers driven by well-characterized genetic mutations, UPS lacks recurrent oncogenic drivers, limiting the development of targeted therapies (5, 6). Identifying genetic drivers in UPS could accelerate the discovery of effective therapeutic strategies for this challenging disease.

Research on human UPS is constrained by the limited availability of biospecimens and reagents, which hampers basic investigations and therapeutic testing. The genetic complexity and absence of well-defined driver mutations further complicate the development of targeted treatments. Genetically engineered mouse models that recapitulate subsets of human UPS are essential tools for studying disease biology and evaluating novel therapies. Several primary mouse models have been established, including primary tumors driven by activation of conditional oncogenic *Kras^G12D^* and deletion of *Trp53* (KP) or *Cdkn2a* (KI), deletion of both *Trp53* and *Pten* (PP), deletion of *Nf1* with *Trp53* (NP) or *Cdkn2a* (NI), and deletion of both *Trp53* and *Rb1* (PR) (7–11). These models have been instrumental in elucidating mechanisms of sarcomagenesis, metastasis, and immune evasion, and in guiding immunotherapeutic strategies (3, 10, 12–16). However, the relevance of these models to human UPS is limited. For instance, *KRAS^G12D^* is an oncogenic driver that is rarely found in human UPS, while *NF1* and *PTEN* are tumor suppressor drivers mutated only in a small subset of human UPS. The molecular pathogenesis of most human UPS remains unknown. After *TP53*, *RB1* is the second most frequently mutated gene in UPS, often through loss-of-function mutations or copy number alterations (CNAs). Yet, compared with KP models, mice with spatial and temporal deletion of *Trp53* and *Rb1* develop sarcomas less frequently and with delayed onset (10). Whether additional genetic alterations could cooperate with *Trp53* and *Rb1* loss to accelerate tumorigenesis remains unexplored.

UPS frequently harbors loss-of-function mutations in tumor suppressor genes, prompting the need to identify those that may cooperate with *Trp53* and/or *Rb1* loss to drive sarcomagenesis. Among these candidates, BRCA1-associated protein 1 (*BAP1*) stands out as a multifunctional tumor suppressor recurrently mutated in uveal melanoma, mesothelioma, cutaneous melanoma, renal cell carcinoma (RCC), and a subset of STSs (17–19). Germline *BAP1* mutations define a tumor predisposition syndrome (BAP1-TPDS), and STS has been reported in affected individuals (20). Transgenic mouse models have demonstrated that *Bap1* deletion can increase malignancy of mesothelioma, RCC, and other cancers (21–24). Additionally, it is suggested that *BAP1* mutations are a late-stage event during cancer progression (25). Furthermore, the role of *BAP1* in STS remains uncharacterized. *BAP1* encodes a deubiquitylase that forms the polycomb repressive–deubiquitylase (PR-DUB) complex, which reverses monoubiquitylation of histone H2A at lysine 119 (H2AK119ub1), a mark deposited by polycomb repressive complex 1 (PRC1) (26). Loss of *BAP1* results in widespread H2AK119ub1 accumulation, leading to global chromatin compaction, activation of DNA damage response, and impaired DNA repair, ultimately compromising cell survival (27). These findings support a broader role for *BAP1* as an epigenetic regulator of tumorigenesis (28–30). Given this background and the lack of clarity regarding *BAP1*’s function in UPS, we included *Bap1* among the candidate genes in our tailored in vivo CRISPR screening library to investigate its contribution to sarcoma development.

In addition to its role in tumorigenesis, emerging evidence suggests that genetic alterations in tumor suppressor pathways may create context-specific therapeutic vulnerabilities. One pathway of interest is signaling through polo-like kinase 1 (*PLK1*), a key mitotic regulator involved in G_2_/M transition, centrosome maturation, and sister chromatid segregation, as well as non-mitotic processes such as DNA replication and the DNA damage response (31–35). *PLK1* is frequently overexpressed in aggressive cancers and has been shown to promote tumorigenesis in multiple model systems, including STSs (35, 36). Although recurrent *PLK1* alterations are not commonly observed in UPS, elevated PLK1 activity may represent a broader feature of highly proliferative sarcomas and could contribute to tumor progression and therapeutic resistance. Notably, prior studies have suggested that epigenetic regulators can influence *PLK1* expression in specific cellular contexts. For example, ChIP-seq analysis has shown that *Bap1* binds to the promoter of *Plk1* in B cells, with increased H2AK119ub1 levels observed upon *Bap1* loss, suggesting potential transcriptional regulation (37). However, whether a similar regulatory relationship exists in sarcoma remains unclear. Based on these observations and the known role of *PLK1* in cancer biology, we investigated the functional importance of *PLK1* signaling in sarcoma models and evaluated whether pharmacologic PLK1 inhibition could represent a therapeutic strategy, including in the context of *Bap1* loss.

## Results

### Intramuscular injection of a CRISPR sgRNA library induces spontaneous sarcomas in mice.

**Figure 1:**
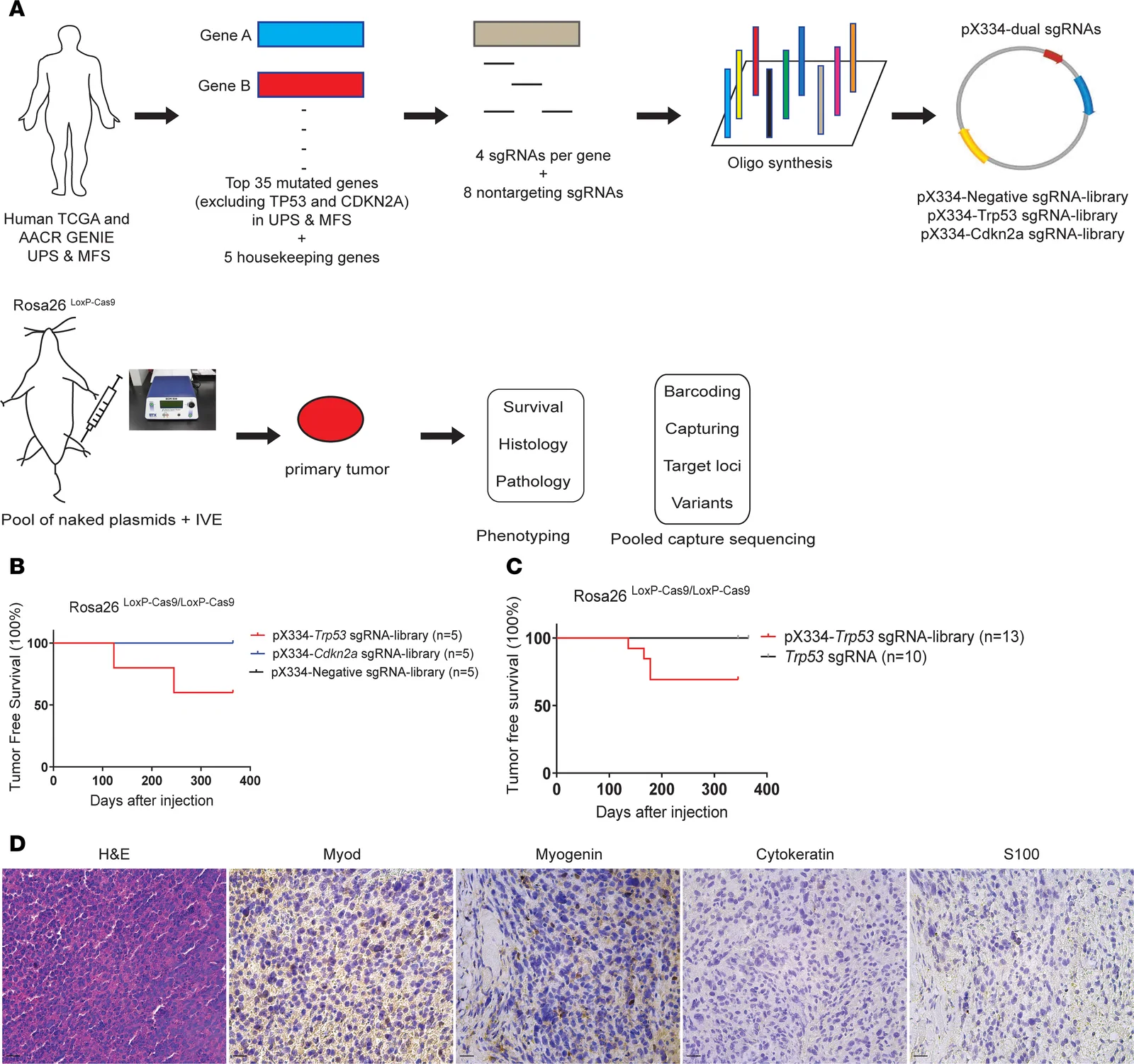
In vivo CRISPR/Cas9 screen identifies tumor suppressors of UPS in Rosa26^loxP-Cas9^ mice. (**A**) Schematic of the direct in vivo CRISPR/Cas9 screening strategy. (**B**) Two primary tumors developed in Rosa26^loxP-Cas9/loxP-Cas9^ mice following intramuscular delivery of the pX334–*Trp53* sgRNA library (*n* = 5), while no tumors formed in mice receiving the pX334–Negative sgRNA library or pX334–*Cdkn2a* sgRNA library (*n* = 5 each). (**C**) In a separate cohort, 5 tumors developed in mice injected with the pX334–*Trp53* sgRNA library (*n* = 13), but no tumors formed in mice injected with pX334–*Trp53* sgRNA alone (*n* = 10). (**D**) Representative H&E and IHC staining (MyoD, myogenin, cytokeratin, S100) of a tumor induced by the pX334–*Trp53* sgRNA library. Scale bars: 100 μm. For all experiments, *n* denotes biologically independent mice. Representative images are from experiments performed at least twice with similar results.

To investigate the tumor suppressor function of genes frequently altered in human UPS, we employed a direct in vivo autochthonous screening strategy. This approach enables targeted mutagenesis within the native skeletal muscle microenvironment of mice (38). Using data from The Cancer Genome Atlas (TCGA) and the Genomics Evidence Neoplasia Information Exchange (GENIE), we identified the top 35 genes with loss-of-function mutations or CNAs in human UPS and myxofibrosarcoma (MFS), which share similar genetic profiles (Supplemental Table 1; supplemental material available online with this article; https://doi.org/10.1172/jci.insight.192686DS1), along with tumor protein p53 (*TP53*) and cyclin-dependent kinase inhibitor 2A (*CDKN2A*) (39). We designed a custom single-guide RNA (sgRNA) library targeting these 35 genes (excluding *Trp53* and *Cdkn2a*), with 4 sgRNAs per gene sourced from the mouse Brie library (Supplemental Table 2) (40). Additionally, we included sgRNAs targeting 5 housekeeping genes: ribosomal protein L7 (*Rpl7*), ribosomal protein 19 (*Rps19*), ribosomal protein L22 (*Rpl22*), ribosomal protein 18 (*Rps18*), and ribosomal protein 11 (*Rps11*) (38). The customized sgRNA library also included 8 nontargeting control sgRNAs from the mouse Brie library (Supplemental Table 2). Given that *TP53* is mutated in approximately 65% of human UPSs and MFSs (39), and that deletion of *Cdkn2a* can substitute for *Trp53* loss to induce sarcomagenesis in conjunction with *Kras^G12D^* (41), we cloned this customized sgRNA library into 3 different plasmid backbones: (a) negative control sgRNA–expressing plasmid (pX334–Negative sgRNA library); (b) *Trp53* sgRNA–expressing plasmid (pX334–*Trp53* sgRNA library); and (c) *Cdkn2a* sgRNA–expressing plasmid (pX334–*Cdkn2a* sgRNA library) (Figure 1A). The *Trp53* sgRNA and *Cdkn2a* sgRNA in these constructs have been confirmed to induce robust and specific editing in the targeting loci, according to previous studies (41, 42). All 3 plasmid libraries were sequenced to confirm full coverages of all sgRNAs (Supplemental Figure 1). We used an in vivo electroporation (IVE) method to deliver these plasmids into the gastrocnemius muscle of Cas9-expressing mice, enabling CRISPR/Cas9–mediated mutagenesis of tumor suppressor genes and generation of UPS-like tumors (11, 41). Only the pX334–*Trp53* sgRNA library induced tumors in both Rosa26^loxP-Cas9/loxP-Cas9^ and Rosa26^loxP-Cas9/+^ mice (Figure 1B and Supplemental Figure 2). In an independent cohort, we confirmed that the pX334–*Trp53* sgRNA library induced tumors in Rosa26^loxP-Cas9/loxP-Cas9^ mice, whereas the pX334–*Trp53* sgRNA alone (lacking sgRNAs for other tumor suppressors) failed to induce tumors (Figure 1C). These results suggest that mutation of at least one additional gene from the library is required to cooperate with *Trp53* loss in driving tumorigenesis.

To characterize the tumors induced by the pX334–*Trp53* sgRNA library, we performed hematoxylin and eosin (H&E) staining and immunohistochemistry (IHC) on sections from 10 tumors using antibodies against cytokeratin, MyoD, myogenin, and S100 (Figure 1D). A blinded pathological review determined that all tumors were poorly differentiated, negative for cytokeratin and S100, and weak or negative for MyoD and myogenin, consistent with UPS or myogenic UPS phenotypes.

### Targeted-capture sequencing identifies multiple tumor suppressor candidates from the Trp53 library–induced tumors.

We generated a total of 14 tumors in mice using the pX334–*Trp53* sgRNA library alone, and an additional 3 tumors following co-delivery of the pX334–*Trp53* sgRNA library with a *Pten* sgRNA (Supplemental Figure 3). To validate CRISPR/Cas9–mediated gene editing using our IVE method, we injected the pX334–*Trp53* sgRNA library into the gastrocnemius muscle of 2 mice. Four weeks after injection, the muscle tissues were harvested, and genomic DNA was extracted from both tumors (S1–S17) and injected muscle tissues (S18–S19) for targeted-capture sequencing (38).

**Figure 2:**
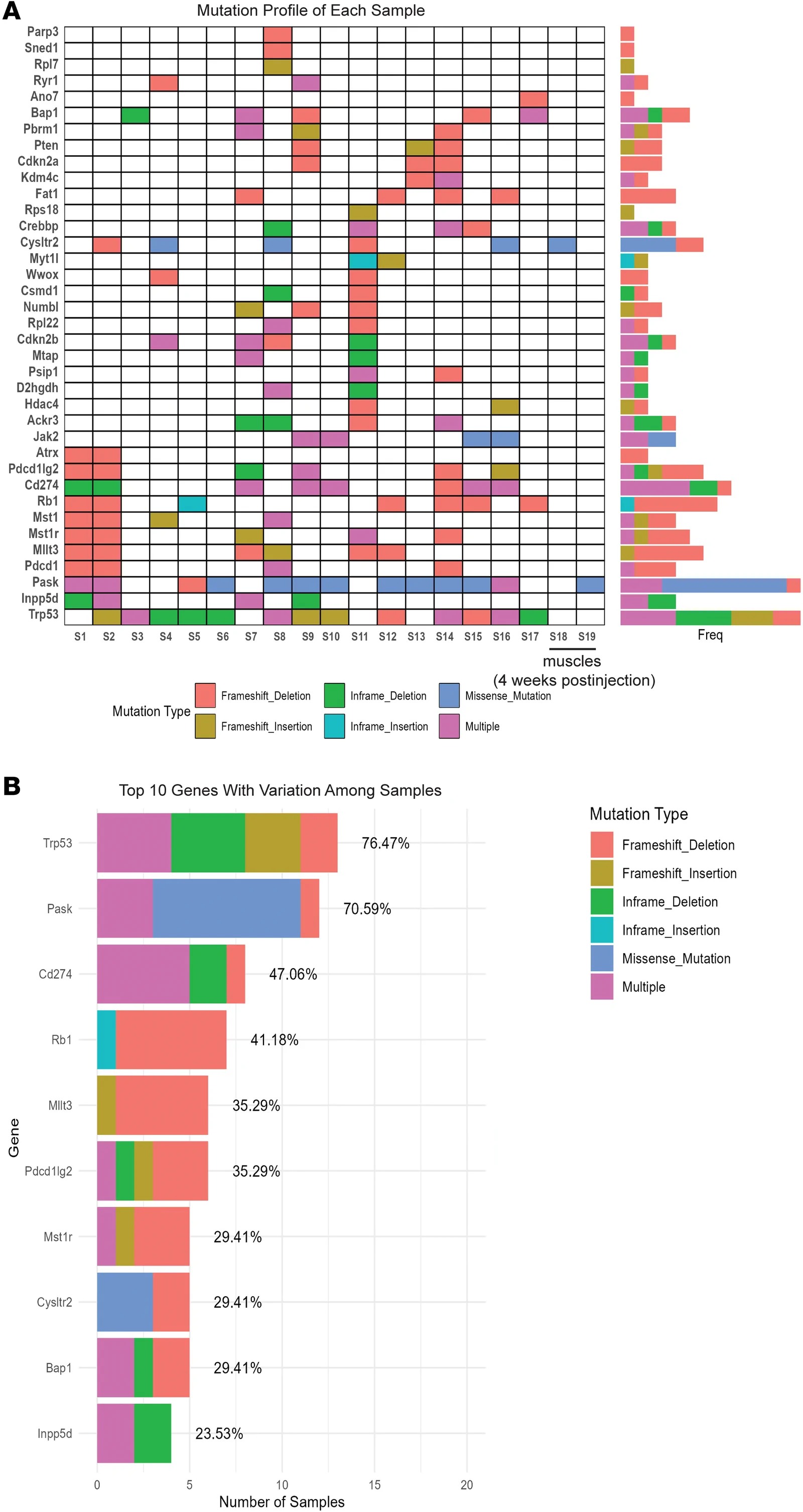
Targeted-capture sequencing of tumors induced by the pX334–*Trp53* sgRNA library. (**A**) Mutation profiles from targeted-capture sequencing of injected muscle tissues (S18–S19) and tumors (S1–S17), including 3 tumors with additional *Pten* mutations (S9, S13, S14). (**B**) Top 10 most frequently mutated genes across all tumors.

Bioinformatic analysis revealed CRISPR/Cas9–mediated editing in *Cysltr2* (S18) and *Pask* (S19) in the injected muscle tissues. However, editing was not detected in most targeted genes, including *Trp53*, in these samples. This may be due to the absence of transformed cells harboring *Trp53* mutations in the harvested tissues or the inability of our sequencing method to detect low-frequency edits in underrepresented mutant cells. Interestingly, *CYSLTR2* ranks among the top 3 genes with loss-of-function mutations or CNAs in human UPS and MFS, following *TP53* and *RB1*. *PASK* also shows the highest mutation frequency in UPS and MFS compared with other cancers in the TCGA database. These findings confirm that our IVE method can induce gene editing in vivo using pooled plasmid libraries (Figure 2A).

**Figure 3:**
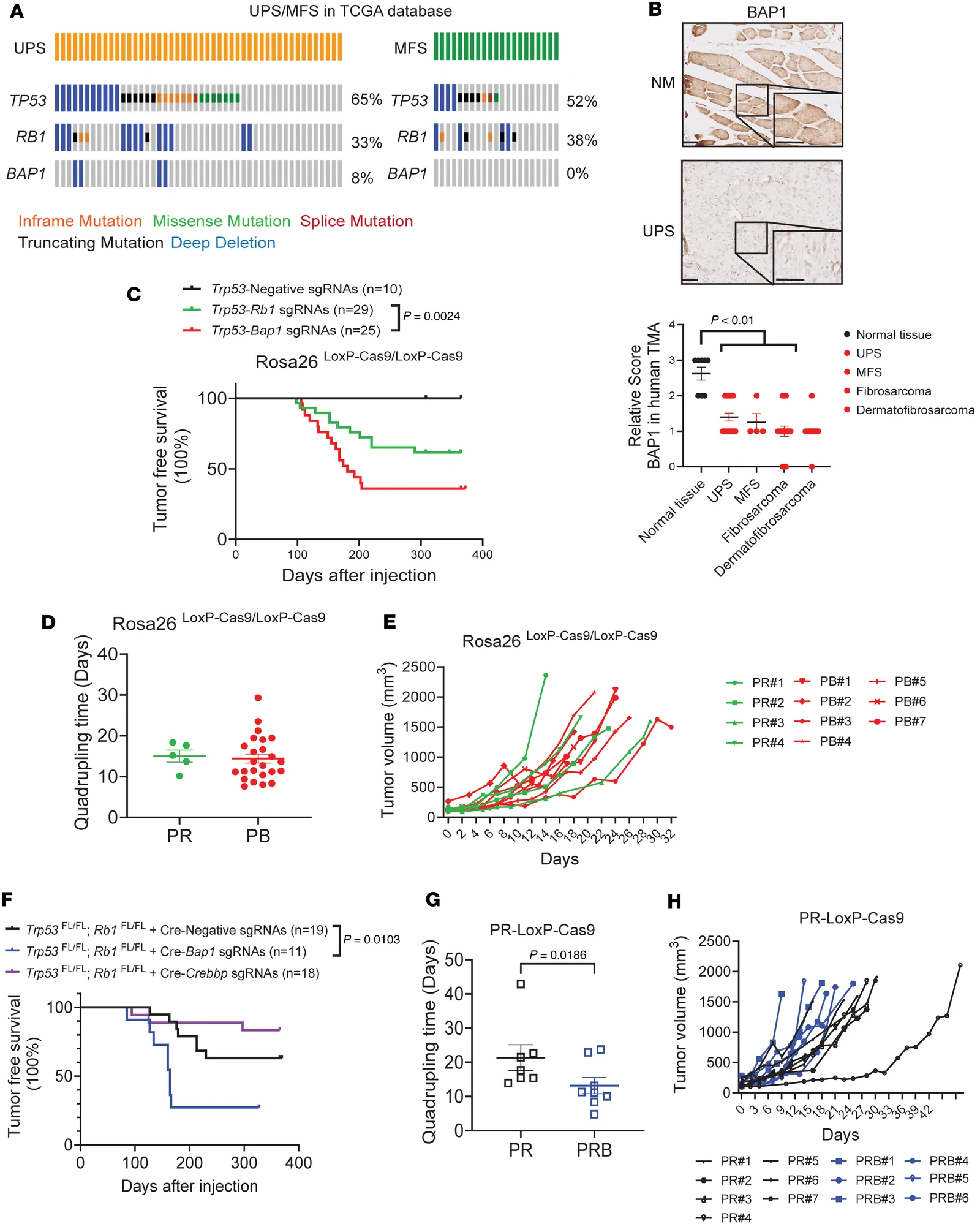
Validation of *Bap1* as a potent tumor suppressor in UPS. (**A**) Mutation rates of 3 genes (*TP53*, *RB1*, and *BAP1*) in human UPS/MFS according to TCGA database. (**B**) IHC of a human sarcoma tissue array shows that BAP1 expression is significantly reduced in UPS, MFS, fibrosarcoma, and dermatofibrosarcoma compared with normal muscle (NM). Scale bars: 50 μm. Quantification was performed on independent tissue cores, and statistical significance was assessed using a 2-tailed Student’s *t* test. (**C**) IVE of plasmids expressing sgRNAs targeting *Trp53* and *Bap1* or *Trp53* and *Rb1* induced tumors in Rosa26^loxP-Cas9/loxP-Cas9^ mice. Tumor-free survival (latency to tumor formation) was analyzed using Kaplan-Meier curves and compared using the log-rank test. (**D**) Tumor quadrupling times were similar between PR CRISPR and PB CRISPR tumors. Statistical comparisons were performed using a 2-tailed Student’s *t* test. (**E**) Growth curves of representative PR and PB CRISPR tumors. (**F**) IVE of Cre recombinase and sgRNA targeting *Bap1* (*n* = 11) induced significantly higher tumor penetrance compared with Cre alone or Cre plus sgRNA targeting *Crebbp* (3 tumors in 18 mice). Tumor-free survival (latency to tumor formation) was analyzed using Kaplan-Meier curves and compared using the log-rank test. (**G**) PRB Cre/CRISPR tumors grew significantly faster than PR Cre tumors. Statistical comparisons were performed using a 2-tailed Student’s *t* test. (**H**) Growth curves of representative PR Cre and PRB Cre/CRISPR tumors. For all experiments, *n* denotes biologically independent mice. Data are presented as mean ± SEM unless otherwise indicated. Representative images and growth curves are from experiments repeated at least twice with similar results.

In tumors generated by co-delivery of the pX334–*Trp53* sgRNA library and the *Pten* sgRNA, *Pten* editing was detected exclusively in those samples, whereas no *Pten* edits were observed in tumors induced by the pX334–*Trp53* sgRNA library alone (Figure 2A and Supplemental Figure 3). Editing of *Trp53* was detected in nearly all tumors, although 4 tumors (S1, S7, S11, and S13) showed no detectable edits by targeted-capture sequencing. To further investigate, we performed T7 endonuclease I (T7E1) assays and Sanger sequencing on genomic DNA from these tumors. In tumor S1, we confirmed 82% insertions and deletions (indels) at the *Trp53* locus, including a 3-nucleotide deletion in one allele (Supplemental Figure 4, A and B). It is possible that the other allele harbors a large deletion undetectable by our method, consistent with previous reports of large CRISPR-induced deletions in *Trp53* (11). Owing to limitations in Sanger sequencing sensitivity (43), we performed next-generation sequencing on tumors S7, S11, and S13. We detected approximately 9% indels in S7, 11% in S11, and 12% in S13, with multiple indel variants per sample (Supplemental Table 3 and Supplemental Figure 4C). The relatively low indel percentages may reflect the presence of normal cells mixed with tumor tissue. Beyond *Trp53*, other frequently mutated genes in the tumors included *Mllt3* (also known as *Af9*), *Fat1*, *Bap1*, and *Rb1* (Figure 2, A and B). Notably, only *Trp53* and *Bap1* were mutated in tumor S3. According to TCGA data, *BAP1* mutations or CNAs are mainly found in UPS among all STS subtypes (Figure 3A). Tumor S6 harbored mutations in *Trp53* and *Pask*. To further investigate the role of these candidate tumor suppressors, we individually evaluated their ability to cooperate with *Trp53* mutation in driving sarcoma development.

### Validation of tumor suppressor genes cooperating with Trp53 mutation to drive sarcoma development in mice.

To evaluate BAP1 expression in human sarcomas, we performed IHC on normal skeletal muscle and a panel of sarcoma specimens, including undifferentiated pleomorphic sarcoma (UPS), myxofibrosarcoma (MFS), fibrosarcoma, and dermatofibrosarcoma, using tissue microarrays stained with BAP1 antibody (Figure 3B). BAP1 protein expression was significantly reduced in UPS, MFS, fibrosarcoma, and dermatofibrosarcoma compared with normal skeletal muscle. To validate candidate tumor suppressor genes identified from the CRISPR screen, we performed intramuscular injections of plasmids expressing sgRNAs targeting individual genes along with *Trp53* sgRNA into Rosa26^loxP-Cas9/loxP-Cas9^ mice. Tumor formation was observed in mice electroporated with plasmids targeting *Trp53* and *Bap1*, as well as those targeting *Trp53* and *Rb1* (Figure 3C). In contrast, no tumors were observed in mice injected with plasmids targeting *Trp53* in combination with *Cysltr2*, *Mst1r*, *Mllt3*, *Crebbp*, *Pbrm1*, or *Fat1* (Supplemental Figure 5). We next compared tumor growth rates between different genotypes. Tumors generated by deletion of *Trp53* and *Rb1* (referred to as PR CRISPR tumors) and those generated by deletion of *Trp53* and *Bap1* (PB CRISPR tumors) showed no significant difference in time to quadrupling of tumor volume (Figure 3, D and E).

To further explore genetic cooperation, we investigated whether additional mutations could accelerate tumorigenesis in the context of *Trp53* and *Rb1* loss. Previous studies in small cell lung cancer models have shown that *Crebbp* deletion accelerates tumor formation when combined with *Trp53* and *Rb1* loss (44). TCGA data also indicate that *BAP1* copy number loss frequently co-occurs with *RB1* loss in human UPS (Figure 3A). Therefore, we generated a transgenic mouse model of *Trp53^fl/fl^* *Rb1^fl/fl^* Rosa26^loxP-Cas9/loxP-Cas9^ (PR-loxP-Cas9 mice) and performed IVE to deliver plasmids expressing Cre recombinase and sgRNAs targeting either no gene, *Bap1*, or *Crebbp*. Targeting *Crebbp* did not affect tumor development initiated by Cre-mediated deletion of *Trp53* and *Rb1* (Figure 3F). In contrast, additional mutation of *Bap1* significantly accelerated tumor onset and increased tumor penetrance in PR-loxP-Cas9 mice (Figure 3F). We then compared tumor growth rates between PR Cre tumors (deletion of *Trp53* and *Rb1*) and PRB Cre/CRISPR tumors (deletion of *Trp53*, *Rb1*, and *Bap1*). PRB Cre/CRISPR tumors exhibited significantly faster growth, with shorter times to quadrupling of tumor volume (Figure 3, G and H). These findings suggest that *Bap1* loss enhances sarcoma aggressiveness in the context of *Trp53* and *Rb1* deficiency.

Additionally, we observed lung metastases in mice bearing PR CRISPR tumors (1/11), PR Cre tumors (1/2), PB CRISPR tumors (4/16), and PRB Cre/CRISPR tumors (1/6). Multiple metastatic nodules were detected in the lungs of mice with PB CRISPR tumors (Supplemental Table 4). Histological analysis via H&E staining confirmed that all tumors were high-grade poorly differentiated sarcomas resembling UPS (Supplemental Figure 6).

Taken together, these results demonstrate that *Bap1* functions as a potent tumor suppressor in UPS, both in the context of *Trp53* loss alone and in combination with *Rb1* loss.

### High-throughput RNA sequencing reveals immune suppression in Bap1-loss mouse sarcomas.

**Figure 4:**
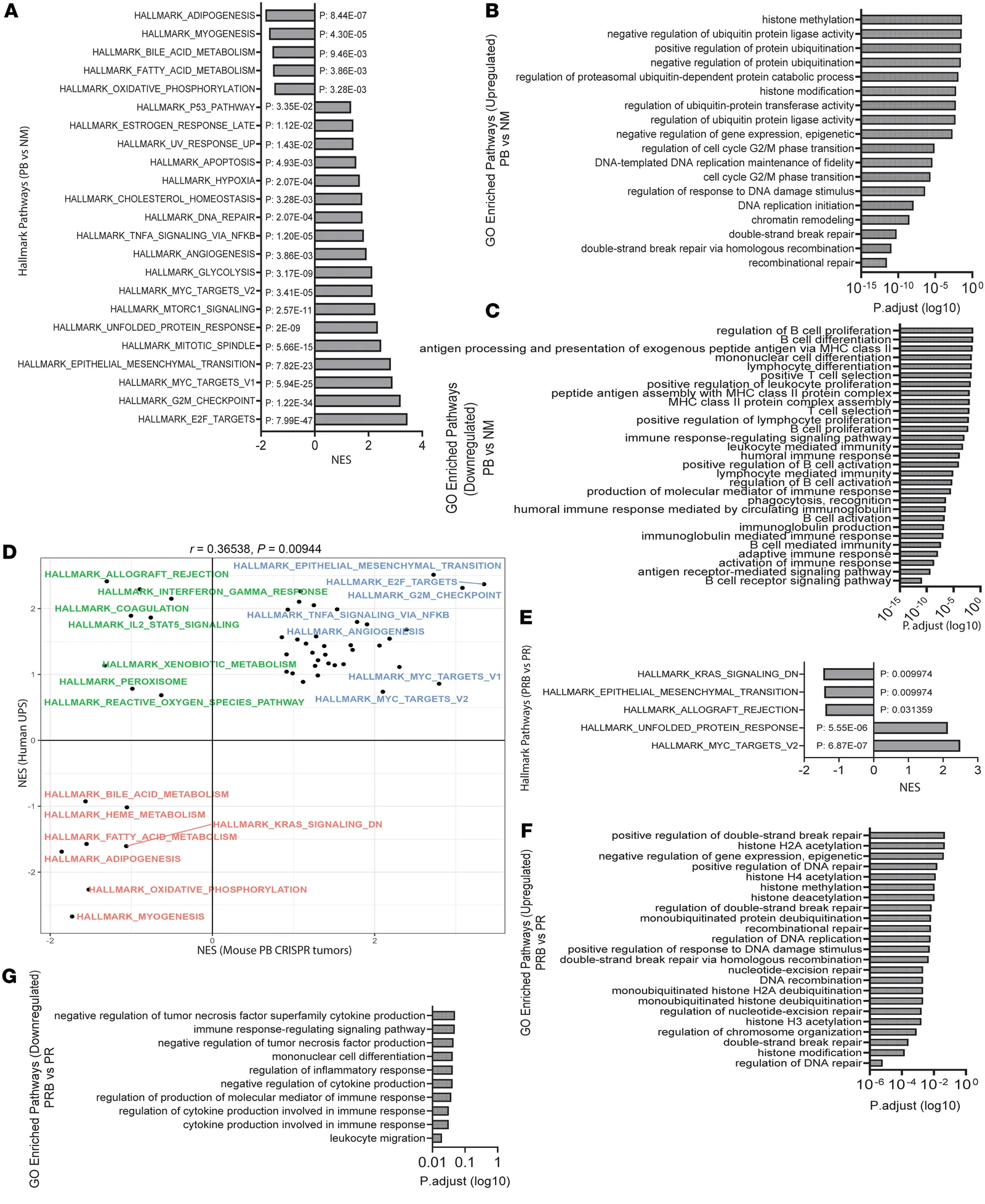
RNA sequencing reveals immune suppression in *Bap1*-deficient sarcomas. (**A**–**C**) PB CRISPR tumors show upregulation of E2F targets, G_2_/M checkpoint, MYC targets, DNA repair, and histone modification pathways and downregulation of immune response pathways versus normal muscle. (**D**) Comparison of hallmark pathways significantly dysregulated in human UPS (TCGA) versus normal human muscle and mouse PB CRISPR tumors versus normal mouse muscle revealed a significant correlation (*R* = 0.36538, *P* = 0.00944). Statistical significance was assessed using Pearson’s correlation analysis. (**E**–**G**) PRB Cre/CRISPR tumors exhibit upregulation of KRAS and MYC pathways and downregulation of B cell signaling and immune activation pathways versus PR Cre tumors. RNA-sequencing analyses were performed on biologically independent samples (PB CRISPR, *n* = 10; PR Cre, *n* = 5; PRB Cre/CRISPR, *n* = 7; normal muscle, *n* = 5). For GSEA, normalized enrichment scores and adjusted *P* values (FDR) were calculated as implemented in the fgsea package.

To systematically investigate the signaling pathways contributing to sarcoma development, we performed total RNA sequencing on tumors from 3 genotypes: PB CRISPR (*n* = 10), PR Cre (*n* = 5), and PRB Cre/CRISPR (*n* = 7), along with normal skeletal muscle controls. Bioinformatic analysis and gene set enrichment analysis (GSEA) revealed that PB CRISPR tumors were significantly enriched for oncogenic pathways, including the E2F targets, G_2_/M checkpoint, and MYC targets hallmarks, compared with normal muscle tissue (Figure 4, A and B) (10). Notably, multiple immune-related pathways were significantly downregulated in PB CRISPR tumors relative to normal muscle, including B cell receptor signaling pathways, B cell activation pathways, activation of immune response pathways, and major histocompatibility complex (MHC) class II protein complex assembly pathways (Figure 4, B and C). These findings suggest that *Bap1* loss is associated with an immunosuppressive tumor microenvironment, consistent with previous observations in *BAP1*-driven uveal melanoma (45). Additionally, recent studies have shown that *BAP1* loss leads to downregulation of MHC class II expression in B cell lymphoma (28). To further assess the similarity between PB CRISPR tumors and human UPS, we compared the hallmark pathways significantly regulated in PB CRISPR tumors versus normal muscle with those significantly regulated in human UPS versus normal muscle. This analysis revealed a significant correlation between PB CRISPR tumors and human UPS (Figure 4D; *R* = 0.36538, *P* = 0.00944), further supporting that PB CRISPR tumors recapitulate key molecular features of human UPS.

Given that *Bap1* deletion increased sarcoma aggressiveness in the context of *Trp53* and *Rb1* loss (Figure 3D), we next compared PR Cre tumors with PRB Cre/CRISPR tumors. PRB Cre/CRISPR tumors exhibited significant upregulation of oncogenic pathways, including the hallmarks of KRAS signaling, unfolded protein response, and MYC targets (Figure 4E). These dysregulated pathways may contribute to the enhanced tumor penetrance and aggressiveness observed in PRB Cre/CRISPR tumors (46, 47). Furthermore, PRB Cre/CRISPR tumors showed significant downregulation of immune-related pathways compared with PR Cre tumors, including regulation of tumor necrosis factor (TNF) production, regulation of inflammatory response, and cytokine production in immune response (Figure 4, F and G). Collectively, these results indicate that *Bap1* loss contributes to immune suppression in STSs, potentially facilitating tumor progression and resistance to immune surveillance.

### Immune cell profiling suggests an immunosuppressive microenvironment in Bap1-loss mouse sarcomas.

**Figure 5:**
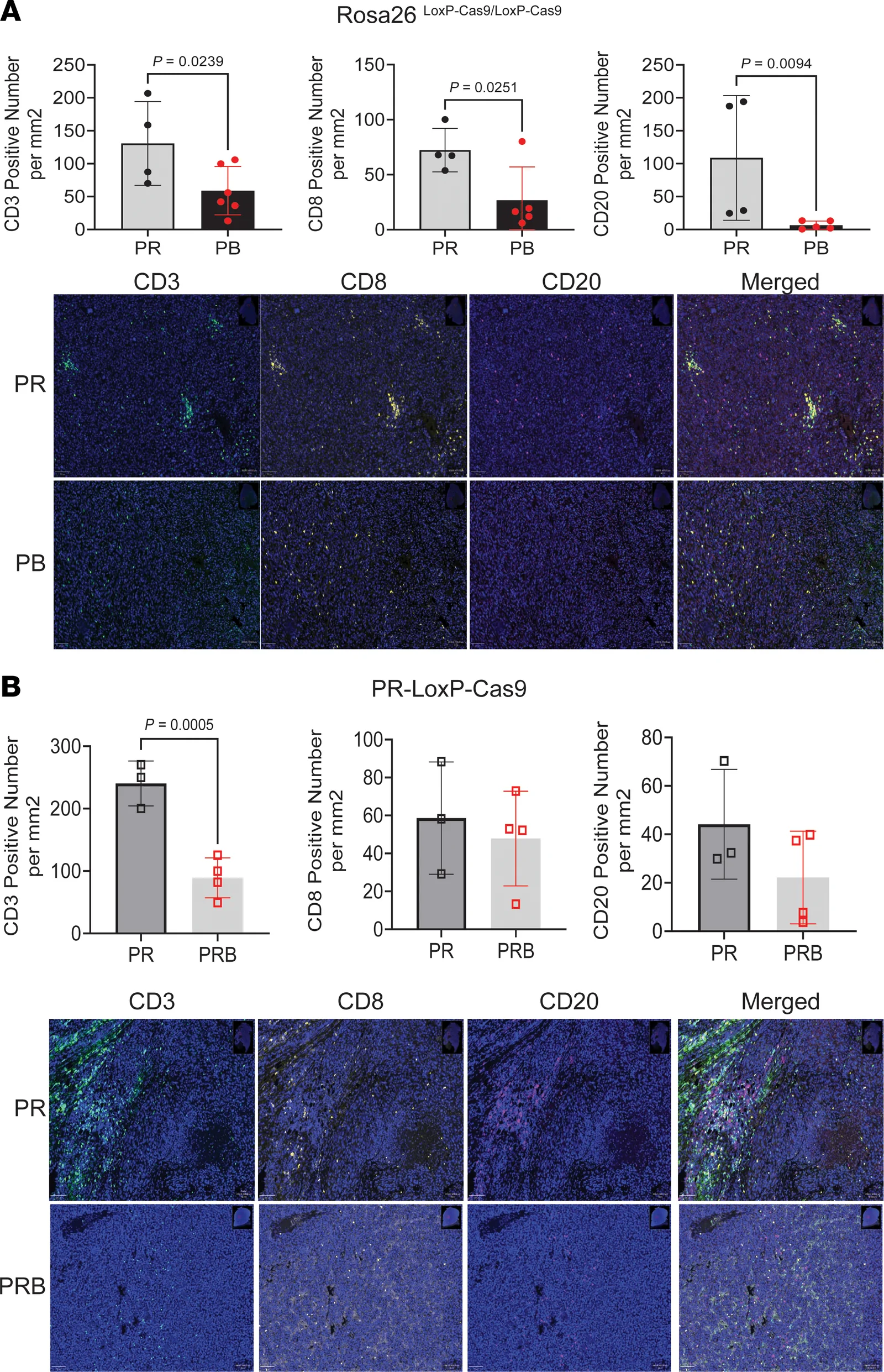
Multiplex IHC reveals reduced immune cell infiltration in *Bap1*-deficient sarcomas. (**A**) PR CRISPR tumors (*n* = 4) show significantly more CD3^+^ T cells than PB CRISPR tumors (*n* = 6). (**B**) PR Cre tumors (*n* = 3) show significantly more CD3^+^ T cells than PRB Cre/CRISPR tumors (*n* = 4). Each point represents an individual tumor. Scale bars: 100 μm. Statistical significance was assessed using a 2-tailed Student’s *t* test. For all experiments, *n* denotes biologically independent tumors. Data are presented as mean ± SEM. Representative images are from experiments performed at least twice with similar results.

To examine the tumor immune microenvironment in mouse sarcomas driven by different genetic mutations, we performed multiplex IHC (48) and flow cytometry on primary tumors (49). Using a multiplex IHC panel including CD3, CD8, CD68, PD-1, CD20, and DAPI, we observed minimal infiltration of CD3^+^ T cells, CD8^+^ T cells, and CD20^+^ B cells in KP mouse sarcomas (driven by *Kras^G12D^* activation and *Trp53* deletion in *Kras^LSL-G12D/+^ Trp53^fl/fl^* mice), consistent with previous reports (*n* = 1, data not shown) (50, 51). In contrast, PR CRISPR tumors (*n* = 4) exhibited significantly higher infiltration of CD3^+^ T cells, CD8^+^ T cells, and CD20^+^ B cells compared with PB CRISPR tumors (*n* = 5) (Figure 5A). To further validate the impact of *Bap1* loss on immune cell infiltration, we compared PR Cre tumors (*n* = 3) with PRB Cre/CRISPR tumors (*n* = 4). PR Cre tumors showed significantly more CD3^+^ T cells, while CD8^+^ T cell and CD20^+^ B cell levels were comparable between groups (Figure 5B). CD68^+^ macrophages were abundant across all tumor types, consistent with findings in human UPS (Supplemental Figure 7) (52, 53).

**Figure 6:**
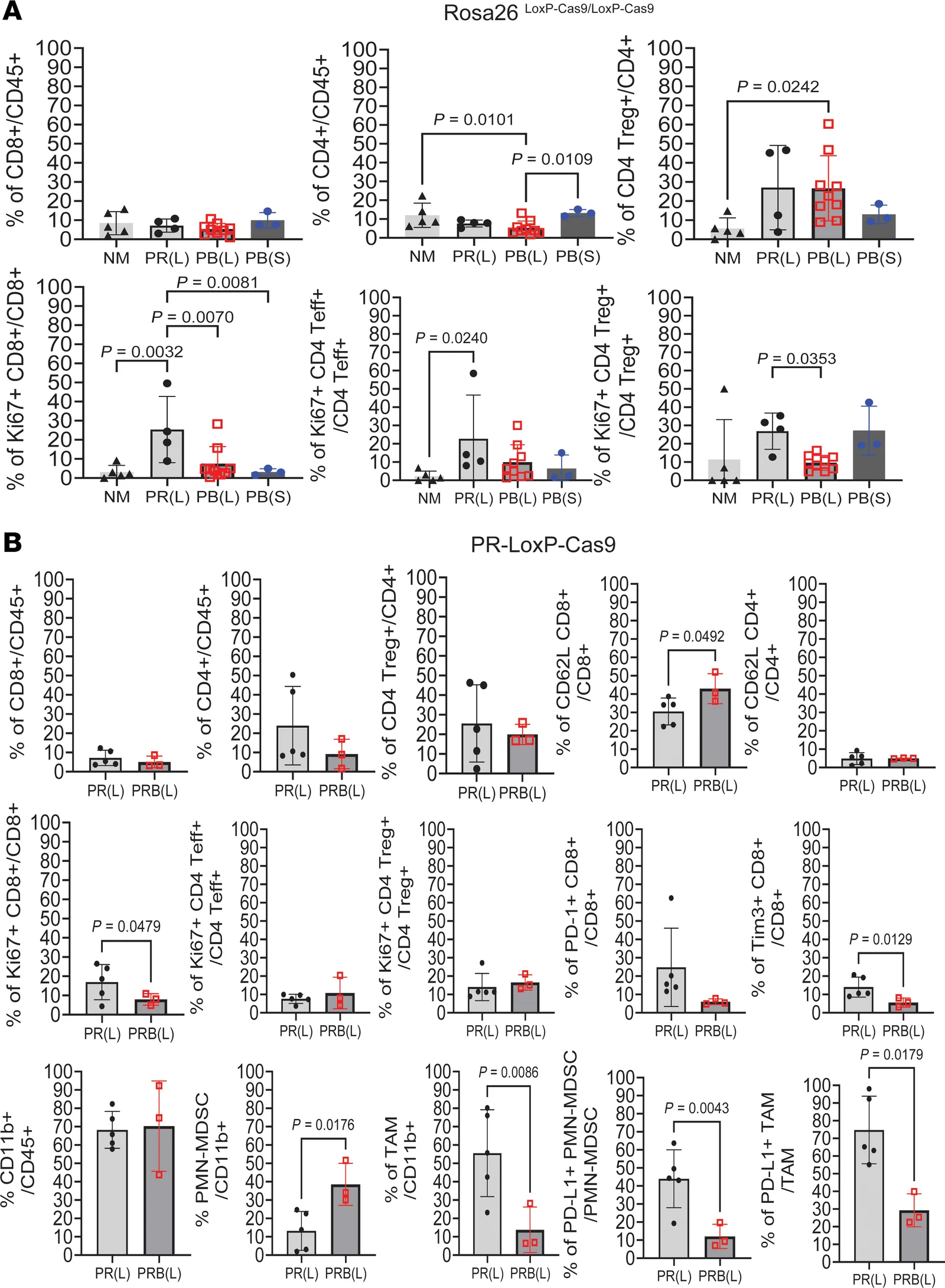
Flow cytometry analysis reveals immunosuppressive features in *Bap1*-deficient sarcomas. (**A**) No differences in CD8^+^ T cell percentages among CD45^+^ cells across NM (*n* = 5), PR CRISPR large (*n* = 5), PB CRISPR large (*n* = 9), and PB CRISPR small (*n* = 3) tumors. CD4^+^ T cell percentages were lower in PB CRISPR large tumors, with higher Treg/CD4^+^ ratios. PR CRISPR large tumors had more Ki-67^+^ CD8^+^ T cells and FoxP3^–^ CD4^+^ effector T (Teff) cells. (**B**) No differences in CD8^+^ T cell, CD4^+^ T cell, or Treg percentages between PR Cre (*n* = 5) and PRB Cre/CRISPR (*n* = 3) tumors. PR Cre tumors had more Ki-67^+^ CD8^+^ T cells, Tim3^+^ CD8^+^ T cells, and CD11b^+^ cells. PRB Cre/CRISPR tumors had higher PMN-MDSC percentages and lower TAM percentages. PD-L1^+^ PMN-MDSCs were more abundant in PR Cre tumors. Each point represents an individual tumor or muscle. Statistical comparisons among multiple groups in **A** were performed using 1-way ANOVA followed by appropriate post hoc tests. Comparisons between 2 groups in **B** were performed using a 2-tailed Student’s *t* test. For all experiments, *n* denotes biologically independent samples. Data are presented as mean ± SEM.

We next used flow cytometry to analyze immune cell populations in tumors of varying sizes. We compared normal muscle (NM; *n* = 5), PR CRISPR large tumors (*n* = 4), PB CRISPR small tumors (<250 mm^3^; *n* = 3), and PB CRISPR large tumors (>1,000 mm^3^; *n* = 9) (Figure 6A). No significant differences were observed in CD8^+^ T cells among CD45^+^ cells. However, CD4^+^ T cell percentages were significantly lower in PB CRISPR large tumors compared with NM and PB early tumors. Notably, the ratio of CD4^+^ regulatory T cells (Tregs) to CD4^+^ T cells was significantly elevated in PB CRISPR large tumors. Further analysis revealed a higher frequency of proliferating (Ki-67^+^) CD8^+^ T cells in PR CRISPR large tumors compared with NM, PB large, and PB small tumors. Additionally, Ki-67^+^ FoxP3^+^ CD4^+^ Tregs were more abundant in PR CRISPR large tumors than in PB CRISPR large tumors. Although Ki-67^+^ Treg levels were lower in PB CRISPR large tumors than in PB small tumors, the difference was not statistically significant. PD-1 expression on CD8^+^ T cells did not differ significantly across tumor types (Supplemental Figure 8). These findings suggest that CD8^+^ T cells are more proliferative in PR CRISPR tumors than in PB CRISPR tumors.

We also profiled immune cells in PR Cre tumors (*n* = 5) and PRB Cre/CRISPR tumors (*n* = 3) (Figure 6B). PR Cre tumors exhibited significantly higher frequencies of proliferating (Ki-67^+^) CD8^+^ T cells and reduced expression of CD62L, a marker of T cell activation (54). Tim3^+^ CD8^+^ T cells were significantly lower in PR Cre tumors, while PD-1^+^ CD8^+^ T cell levels were similar between groups. These results suggest that CD8^+^ T cells in *Bap1*-wild-type tumors (PR Cre) are more proliferative and activated than those in *Bap1*-deficient tumors (PRB Cre/CRISPR).

We further examined myeloid-derived suppressor cells (MDSCs) and tumor-associated macrophages (TAMs). CD11b^+^ cell percentages among CD45^+^ cells were significantly higher in PB CRISPR large tumors compared with PB early tumors (Supplemental Figure 9). No differences were observed in CD11b^+^ cell percentages between PR Cre and PRB Cre/CRISPR tumors (Figure 6B). Additionally, there were no significant differences in polymorphonuclear MDSCs (PMN-MDSCs; CD11b^+^Ly6C^int^Ly6G^hi^), monocytic MDSCs (M-MDSCs; CD11b^+^Ly6C^hi^Ly6G^lo^), or TAMs (CD11b^+^Ly6C^lo^Ly6G^lo^F4/80^+^) between PB CRISPR large and small tumors (Supplemental Figure 9). Interestingly, PMN-MDSC percentages were significantly higher in PRB Cre/CRISPR tumors, while TAM percentages were higher in PR Cre tumors. M-MDSC levels were similar between groups. PD-L1, an immunosuppressive ligand that binds PD-1 (55), was significantly lower on PMN-MDSCs, M-MDSCs, and TAMs in PRB Cre/CRISPR tumors compared with PR Cre tumors (Figure 6B and Supplemental Figure 9). Although PD-L1 expression is generally associated with poor prognosis in STS, its role in *Bap1*-deficient sarcomas warrants further investigation (56). Together, these findings suggest that *Bap1* deficiency is associated with features of an immunosuppressive tumor immune microenvironment in STS, including reduced T cell activation and proliferation, increased Treg ratios, and altered myeloid cell composition.

### Plk1 knockdown inhibits growth of Bap1-loss mouse sarcomas.

We established multiple mouse sarcoma cell lines derived from PR and PB CRISPR tumors and confirmed knockout of the targeted genes via Western blotting (Supplemental Figure 10). As expected, RB1 protein was absent in both tested PR sarcoma cell lines, and BAP1 protein was absent in both tested PB sarcoma cell lines. In 3T3 cells, wild-type p53 was robustly induced by the DNA-damaging agent doxorubicin; however, p53 in all tested PR and PB sarcoma cell lines remained unresponsive to doxorubicin treatment, consistent with loss of functional p53 signaling (11). Given prior evidence that PARP inhibitors (PARPis) are effective in *Bap1*-mutant RCC (57), and that double-strand DNA repair pathways are upregulated in PB CRISPR tumors, we tested whether PARPis could eliminate PR and PB sarcoma cells. Additionally, PARPis have shown efficacy in *Rb1*-mutant osteosarcoma (58). Therefore, we tested whether PARPis could eliminate PR and PB sarcoma cells. In vitro live-cell imaging assays revealed that both PR and PB sarcoma cells were sensitive to two PARPis: niraparib and talazoparib (Supplemental Figure 11). However, in vivo treatment of nude mice bearing PR or PB tumors with niraparib (50 mg/kg daily via oral gavage for 28 days) showed significant antitumor effects only in PR tumors, not in PB tumors (Supplemental Figure 11). These results suggest that PR tumors are sensitive to PARP inhibition in vivo, while PB tumors are resistant.

**Figure 7:**
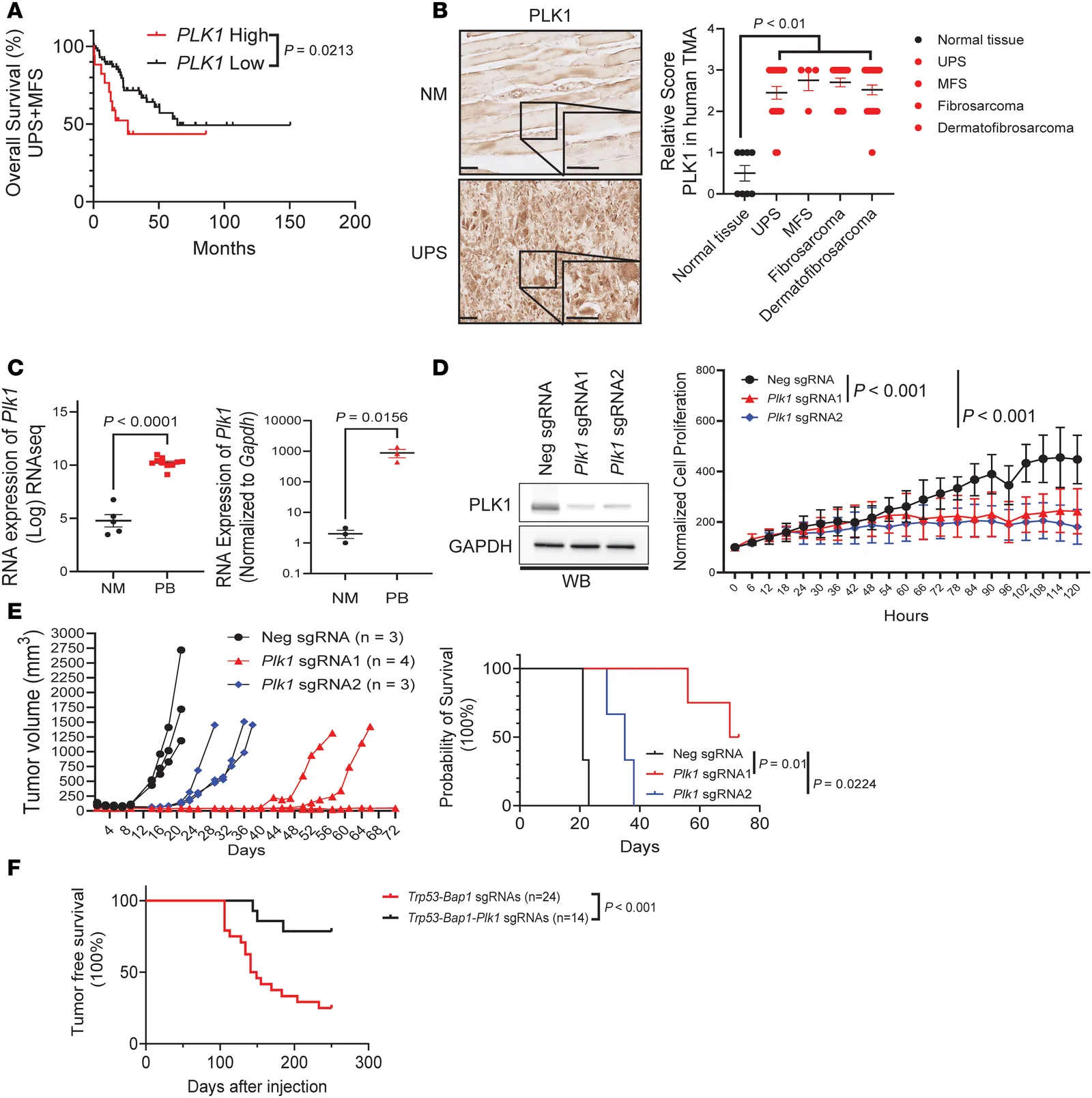
*Plk1* knockdown inhibits growth of *Bap1*-deficient sarcomas. (**A**) High *PLK1* RNA expression correlates with poor prognosis in UPS (TCGA). Survival differences were analyzed using Kaplan-Meier curves and compared using the log-rank test. (**B**) IHC of a human sarcoma tissue array shows that PLK1 protein expression is significantly increased in UPS, MFS, fibrosarcoma, and dermatofibrosarcoma. Scale bars: 50 μm. Quantification was performed on independent tissue cores, and statistical significance was assessed using a 2-tailed Student’s *t* test. (**C**) RNA sequencing and quantitative reverse transcription PCR confirm *Plk1* upregulation in PB sarcomas. Statistical comparisons were performed using a 2-tailed Student’s *t* test. (**D**) Western blot confirms PLK1 knockdown; cell proliferation assays (CELLCYTE) demonstrate reduced growth following *Plk1* knockdown. Statistical comparisons were performed using a 2-tailed Student’s *t* test. (**E**) *Plk1* knockdown inhibits tumor growth and prolongs survival in a syngeneic mouse model. Survival was analyzed using Kaplan-Meier curves with significance assessed by the log-rank test. (**F**) CRISPR/Cas9–mediated mutation of *Plk1* reduces the penetrance of *Trp53*/*Bap1* loss–driven sarcomagenesis. Survival was analyzed using Kaplan-Meier curves with significance assessed by the log-rank test.

Polo-like kinase 1 (*PLK1*), a serine/threonine protein kinase, regulates key mitotic processes, including G_2_/M transition, centrosome maturation, and sister-chromatid segregation (31, 32). *PLK1* also plays important non-mitotic roles, such as coordinating DNA replication and the DNA damage response (31, 32). Consistent with these functions, pathways governed by *PLK1* are significantly dysregulated in PB CRISPR tumors (Figure 4, A and B). Using *Plk1*-transgenic mice, Gheghiani et al. demonstrated that *Plk1* overexpression drives the development of multiple tumor types, including sarcoma, providing strong rationale for investigating the role of *Plk1* in *Bap1*-driven sarcomagenesis (35). Using BAP1 chromatin immunoprecipitation followed by sequencing (ChIP-seq), we identified BAP1 binding at the *PLK1* genomic locus (28). BAP1, a deubiquitylase, forms the PR-DUB complex to reverse H2AK119ub1 modifications catalyzed by PRC1 (26, 59). *Bap1* knockout increased H2AK119ub1 levels upstream of the *PLK1* transcription start site, suggesting epigenetic regulation of *PLK1* by BAP1 (28, 37). Analysis of TCGA data revealed that high *PLK1* expression correlated with poor prognosis in UPS patients (Figure 7A). To evaluate PLK1 expression in human sarcomas, we performed IHC on normal skeletal muscle and a panel of sarcoma specimens, including undifferentiated pleomorphic sarcoma (UPS), myxofibrosarcoma (MFS), fibrosarcoma, and dermatofibrosarcoma, using tissue microarrays stained with PLK1 antibody (Figure 7B). PLK1 protein expression was significantly upregulated in UPS, MFS, fibrosarcoma, and dermatofibrosarcoma relative to normal skeletal muscle.

Total RNA sequencing and reverse transcription PCR confirmed that *Plk1* was significantly upregulated in PB sarcomas compared with normal muscle (Figure 7C). To investigate the oncogenic role of *PLK1*, we stably transduced Cas9-expressing PB sarcoma cell lines with lentiviruses expressing either a nontargeting control sgRNA (neg sgRNA) or sgRNAs targeting *Plk1*. We confirmed reduced PLK1 expression via Western blot (Figure 7D). In vitro proliferation assays showed that *Plk1* knockdown significantly inhibited PB sarcoma cell growth (Figure 7D). To assess the effect of *Plk1* knockdown in vivo, we injected Rosa26^loxP-Cas9/loxP-Cas9^ mice intramuscularly with PB sarcoma cells stably expressing neg sgRNA (*n* = 3), *Plk1* sgRNA1 (*n* = 4), or *Plk1* sgRNA2 (*n* = 3). *Plk1* knockdown significantly suppressed tumor growth (Figure 7E). Notably, 2 of 4 mice injected with *Plk1* sgRNA1–transduced cells did not develop tumors over a 72-day period. Moreover, *Plk1* knockdown significantly prolonged mouse survival (Figure 7E). Next, we examined whether *Plk1* upregulation is required for *Trp53*/*Bap1* loss–driven sarcomagenesis. We generated a plasmid expressing 3 sgRNAs targeting *Trp53*, *Bap1* (sgRNA4), and *Plk1* (sgRNA1) and delivered it via IVE into the gastrocnemius muscles of Rosa26^loxP-Cas9/loxP-Cas9^ mice (*n* = 14). For comparison, a plasmid expressing sgRNAs targeting *Trp53* and *Bap1* alone was delivered into a separate cohort of mice (*n* = 24). Additional CRISPR/Cas9–mediated mutation of *Plk1* significantly reduced the penetrance of *Trp53/Bap1* loss–driven sarcomagenesis (Figure 7F). Collectively, these findings demonstrate that *PLK1* is essential for the development and maintenance of *Bap1*-deficient sarcomas and identify PLK1 as a promising therapeutic target.

### Pharmaceutical inhibition of PLK1 as a targeted therapy for Bap1-deficient sarcomas.

**Figure 8:**
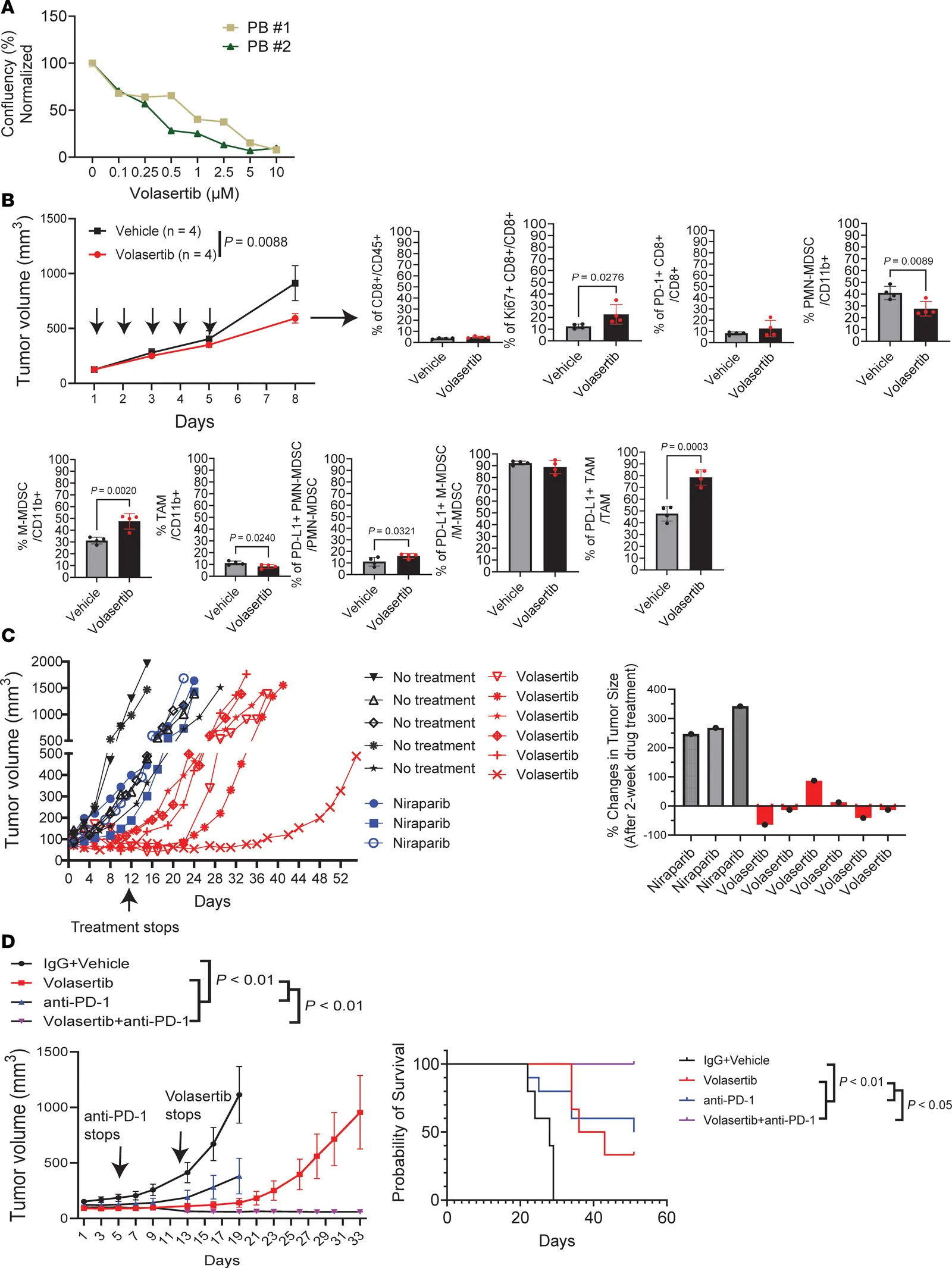
Pharmacologic inhibition of PLK1 suppresses *Bap1*-deficient sarcomas. (**A**) Volasertib inhibits PB sarcoma cell proliferation in vitro in a dose-dependent manner. (**B**) In Rosa26^loxP-Cas9/loxP-Cas9^ mice, volasertib increases Ki-67^+^ CD8^+^ T cells, reduces TAMs, and alters MDSC populations. PD-L1^+^ PMN-MDSCs and TAMs are elevated after treatment. Statistical comparisons were performed using a 2-tailed Student’s *t* test. (**C**) In an autochthonous UPS model, volasertib induces tumor regression in 4 of 6 mice, while niraparib shows no effect. Left: Tumor growth curves. Right: Waterfall plot of tumor size changes after 2-week treatment. (**D**) Therapeutic efficacy of PLK1 inhibition combined with immune checkpoint blockade in a syngeneic PB sarcoma model. C57BL/6J mice bearing intramuscularly transplanted PB sarcomas were treated with vehicle control (IgG), volasertib alone, anti–PD-1 alone, or the combination. Volasertib was administered by oral gavage (10 mg/kg, 5 days per week), and anti–PD-1 antibody was delivered via intraperitoneal injection (100 μg per dose, days 1, 3, and 5). Combination treatment resulted in greater suppression of tumor growth compared with either monotherapy. Tumor growth curves were compared using 1-way ANOVA followed by appropriate post hoc tests. Kaplan-Meier analysis shows that both monotherapies improve survival relative to control, whereas combination therapy provides the most significant survival benefit. For all experiments, *n* denotes biologically independent samples or mice as indicated. Data are presented as mean ± SEM unless otherwise specified.

To evaluate the therapeutic potential of PLK1 inhibition in *Bap1*-deficient sarcomas, we tested the sensitivity of PB mouse sarcoma cells to volasertib, a selective PLK1 inhibitor (60). In vitro assays demonstrated that PB sarcoma cells were sensitive to volasertib in a dose-dependent manner (Figure 8A). To investigate volasertib’s impact on tumor-infiltrating immune cells, we used Rosa26^loxP-Cas9/loxP-Cas9^ mice injected intramuscularly with PB sarcoma cells. Treatment began when tumors reached ≥100 mm^3^, with mice randomized to receive volasertib or vehicle (10 mg/kg, 5 d/wk for 1 week) (Figure 8B). Flow cytometry performed on day 4 after treatment revealed significantly increased frequencies of proliferating (Ki-67^+^) CD8^+^ T cells in the volasertib group; no difference in CD11b^+^ cell percentages among CD45^+^ cells; significantly lower percentages of PMN-MDSCs and TAMs but higher percentages of M-MDSCs in the volasertib group; and higher expression of PD-L1 on PMN-MDSCs and TAMs in volasertib-treated tumors (Figure 8B and Supplemental Figure 12). These findings indicate that volasertib suppresses tumor growth and modulates the tumor immune microenvironment, enhancing CD8^+^ T cell proliferation and altering myeloid cell composition.

Next, we tested volasertib in spontaneous tumors initiated by IVE delivery of *Trp53* and *Bap1* sgRNAs into the gastrocnemius muscle of Rosa26^loxP-Cas9/loxP-Cas9^ mice (Figure 3A). When tumors reached 50–100 mm^3^ (≥100 days after injection), mice were treated with either 50 mg/kg niraparib (*n* = 3) or 10 mg/kg volasertib (*n* = 6) for 2 weeks. Niraparib showed no therapeutic effect, consistent with transplantation model results. In contrast, volasertib induced tumor regression in 4 of 6 mice, with one tumor remaining stable and one showing slow growth (Figure 8C, waterfall plot). Overall, these results demonstrate that pharmaceutical inhibition of PLK1 via volasertib is a promising targeted therapy for *Bap1*-loss sarcomas, with both direct antitumor effects and beneficial modulation of the tumor immune microenvironment.

Finally, we evaluated whether PLK1 inhibition could enhance the therapeutic efficacy of immune checkpoint blockade in vivo using immunocompetent C57BL/6J mice bearing intramuscularly transplanted PB sarcoma cells. When tumors reached approximately 100 mm^3^ (day 14 after injection), mice were randomized into 4 treatment groups: vehicle plus control IgG (*n* = 5), volasertib alone (oral gavage, 10 mg/kg per dose, 5 days per week for up to 2 weeks; *n* = 6), anti–PD-1 alone (intraperitoneal injection, 100 μg per dose on days 1, 3, and 5; *n* = 10), and the combination of volasertib and anti–PD-1 using the same dosing regimens (*n* = 7) (Figure 8D). Relative to the control group, both volasertib and anti–PD-1 monotherapy significantly delayed tumor growth. Notably, the combination treatment resulted in greater tumor growth inhibition compared with either monotherapy. At the end of the treatment period (day 13), tumors were not detectable in mice treated with volasertib; however, tumor regrowth was observed in 4 mice following cessation of volasertib monotherapy. In contrast, mice receiving the combination therapy exhibited more durable tumor control, with tumors remaining undetectable for up to 51 days after treatment initiation. Consistent with these observations, both volasertib and anti–PD-1 monotherapy significantly improved survival compared with control treatment, whereas combination therapy produced the greatest survival benefit (Figure 8D). Collectively, these findings indicate that PLK1 inhibition can enhance the antitumor activity of immune checkpoint blockade in this PB sarcoma model.

## Discussion

Undifferentiated pleomorphic sarcoma (UPS), a major subtype of adult soft-tissue sarcoma (STS), carries a poor prognosis due to frequent recurrence and metastasis, with a median survival often below 15 months (2, 3, 7). Despite its clinical aggressiveness, treatment options for UPS have remained largely unchanged for over four decades. The genetic heterogeneity and absence of recurrent oncogenic driver mutations in UPS make a universal therapeutic approach impractical. Instead, UPS frequently exhibits copy number alterations in tumor suppressor genes, suggesting that identifying actionable targets based on specific tumor suppressor mutations may offer a more effective strategy for therapy development.

Advancing our understanding of UPS biology and immune evasion mechanisms is critical. However, progress has been limited by the absence of spatially and temporally controlled autochthonous models that faithfully replicate human disease. Our earlier genome-scale CRISPR/Cas9 screen in vitro did not reproduce sarcomagenesis in vivo (41). To address this gap, we implemented a customized in vivo CRISPR/Cas9 screen to identify tumor suppressor genes that cooperate with *Trp53* loss to drive UPS formation. This approach revealed that additional genetic alterations beyond *Trp53* are necessary for tumor initiation.

Through targeted validation, we demonstrated that combined loss of *Bap1* and *Trp53* is sufficient to induce sarcomagenesis. Although *BAP1* mutations occur in a minority of STS cases, they are enriched in UPS according to TCGA data (61). Moreover, *BAP1* mutations have been reported in 12% of recurrent pediatric embryonic rhabdomyosarcomas, which share a similar cellular origin with UPS (18, 62–64), and in patients with *BAP1* tumor predisposition syndrome (BAP1-TPDS) (20, 65, 66). Consistent with these findings, we observed significant downregulation of BAP1 protein expression in human UPS, myxofibrosarcoma (MFS), fibrosarcoma, and dermatofibrosarcoma using IHC on human sarcoma tissue arrays. To our knowledge, this study provides the first direct evidence that *Bap1* loss contributes to sarcoma initiation (25). Our UPS model driven by somatic deletion of *Trp53* and *Bap1* offers a unique platform to investigate *BAP1*’s role in tumorigenesis and therapeutic targeting.

*BAP1* functions as a deubiquitylase within the polycomb repressive–deubiquitylase (PR-DUB) complex, which includes host cell factor 1 (*HCFC1*), O-linked *N*-acetylglucosamine transferase (*OGT*), forkhead box K1/2 (*FOXK1/2*), and additional sex combs like 1/2/3 (*ASXL1/2/3*) (30). Mutations in *ASXL1* are common in hematologic malignancies, while *ASXL2* and *ASXL3* are implicated in other cancers (67). *FOXK1* and *FOXK2* also play critical roles in cancer development (68). Notably, the TCGA database shows that *FOXK1*, *FOXK2*, *ASXL2*, *ASXL3*, and *BAP1* are exclusively mutated or copy number–altered in UPS (Supplemental Figure 13). A total of 16% of human UPSs bear mutations or deletions in at least one gene from the PR-DUB complex. A recent study using CRISPR/Cas9–mediated saturation genome editing screening determined that the *ASXL1/2/3*-interacting domain in the BAP1 protein may be critical for its tumor-suppressive function (69). These findings suggest that disruption of the PR-DUB complex by a single gene mutation may promote sarcoma development, an observation that warrants further study.

Beyond tumor initiation, BAP1 influences the immune microenvironment. In uveal melanoma, *BAP1* loss correlates with immune suppression, and similar resistance to PD-1 blockade has been observed in *Bap1*-deficient pancreatic cancer models (45, 70). In our study, RNA sequencing revealed significant downregulation of B cell and immune response pathways in *Bap1*-deficient sarcomas compared with controls. Additionally, multiplex IHC and flow cytometry confirmed poor immune cell infiltration, particularly of proliferating CD8^+^ T cells. Prior research has shown that ablation of BAP1 reduces transcription of MHC class II in B cell lymphomas, contributing to immune suppression (28). Our RNA sequencing results similarly show MHC class II downregulation in *Bap1*-deficient tumors (data not shown). However, the limitation of our present study is that these data are primarily descriptive and do not fully elucidate the underlying molecular mechanisms by which *BAP1* regulates sarcoma-immune interactions. Given the established role of *BAP1* as an epigenetic regulator within the PR-DUB complex, future studies will be required to define how *BAP1* loss reshapes the tumor epigenome to influence immune-related gene expression programs, including antigen presentation, cytokine/chemokine production, and immune checkpoint regulation. Mechanistic interrogation of how *BAP1* loss modulates crosstalk between sarcoma cells and specific immune subsets, such as CD8^+^ T cells, Tregs, and myeloid populations, will help determine whether these phenotypes arise from altered immune recruitment, differentiation, or functional suppression. These studies will be critical to establish a causal framework linking *BAP1*-dependent chromatin regulation to immune evasion and to identify actionable nodes for therapeutic intervention.

Targeted therapy holds promise for genetically diverse UPS. For instance, Li et al. showed that *RB1*- and *TP53*-deficient UPS and MFS are sensitive to SKP2 inhibition (71). Studies showed that *RB1*-mutant osteosarcomas and *BAP1*-mutant RCCs are sensitive to PARPis (57, 58). We investigated the effect of PARPis on PR and PB mouse sarcomas. We found that all tested mouse sarcoma cells showed similar sensitivity to two PARPis, niraparib and talazoparib. However, only PR sarcomas showed significant in vivo sensitivity to niraparib, while PB sarcomas were resistant. These findings prompted us to explore alternative therapeutic vulnerabilities, including targeting PLK1, a serine/threonine kinase implicated in tumor progression, survival, and immune regulation (33–35, 72, 73). We observed that PLK1 protein expression is elevated in multiple human sarcoma subtypes relative to normal tissue. Notably, our data do not establish whether PLK1 upregulation is specifically associated with *BAP1* loss versus a more general feature of sarcoma biology. Consistent with this, while our functional studies demonstrate that PLK1 is required for the growth of *Bap1*-deficient sarcoma models, we did not directly compare PLK1 dependence between *Bap1*-deficient and *Bap1*-intact tumors across multiple systems. Therefore, the extent to which PLK1 represents a selective vulnerability in *BAP1*-deficient sarcomas remains to be clarified.

Using both transplantation and spontaneous mouse models, we further found that *Bap1*-deficient sarcomas are sensitive to pharmacologic PLK1 inhibition with volasertib, and that treatment is associated with changes in the tumor immune microenvironment. Combination therapy with anti–PD-1 enhanced tumor control compared with monotherapy in the model tested. These findings suggest that PLK1 inhibition represents a promising therapeutic strategy for *BAP1*-deficient UPS and may be particularly effective when combined with immunotherapy. Given the broad upregulation of PLK1 across sarcoma subtypes and the established clinical safety of both PLK1 inhibitors and PD-1 blockade, this combination strategy may have wider applicability and warrants further investigation in clinical trials (60, 74). However, the limitation of our present study is that these findings are derived primarily from a limited number of mouse models and do not establish whether the interaction between PLK1 inhibition and immune checkpoint blockade is specific to *BAP1* deficiency or applicable more broadly to sarcomas with high PLK1 expression. Moreover, the biological basis of the observed therapeutic interaction remains incompletely defined, and it is unclear which patient subsets, such as those defined by *BAP1* status, PLK1 expression, or other molecular features, may derive benefit from this approach.

In summary, we present an autochthonous STS model driven by combined *Trp53* and *Bap1* loss that provides a platform to investigate the genetic and immunologic drivers of sarcomagenesis. Our findings support a role for *BAP1* loss in sarcoma initiation and immune modulation. The identification of PLK1 as a potential vulnerability in *Bap1*-deficient sarcomas provides a rationale for exploring targeted therapeutic strategies, including in combination with immune checkpoint blockade, and highlights the interplay between tumor suppressor alterations and the tumor immune microenvironment. The direct mechanistic relationship between *BAP1* and *PLK1* remains incompletely defined. In particular, whether *BAP1* loss confers dependence on *PLK1* through specific epigenetic or post-transcriptional mechanisms requires further investigation. Future studies integrating genetic, epigenomic, and functional approaches across diverse human and mouse sarcoma models will be necessary to define the context-specific role of *PLK1* in *BAP1*-altered and *BAP1*-intact sarcomas, and to evaluate the translational potential of PLK1-targeted strategies, alone or in combination with immune checkpoint blockade.

## Methods

### Sex as a biological variable.

Both male and female mice were used in this study, as noted below. The experiments were not specifically designed or statistically powered to detect sex-dependent differences, and the data were not analyzed separately by sex. Nevertheless, because the tumor models and therapeutic interventions used here are not restricted to one sex, we expect the findings to be broadly relevant to both male and female mice.

### Mice.

Rosa26^loxP-Cas9^ mice were obtained from F. Zhang (Massachusetts Institute of Technology, Cambridge, Massachusetts, USA) (42). Trp53^flox^ mice were obtained from A. Berns (University of Amsterdam, Amsterdam, the Netherlands) (75). Rb1^flox^ mice were provided by I. Gelman (Roswell Park Cancer Institute, Buffalo, New York, USA). For the transplantation study, athymic nude (nu/nu) and C57BL/6 mice at least 6 weeks old were provided by The Jackson Laboratory. The nude mice were maintained in the Earle A. Chiles Research Institute’s accredited animal facility. Both male and female mice were used in experiments.

### Cell culture.

Mouse embryonic fibroblasts were generated from E13.5 to E14.5 embryos using standard procedures (11). NIH 3T3 cell lines (CRL-1658), provided by ATCC, were maintained in DMEM (Cytiva, SH30243.LS) supplemented with 10% fetal bovine serum and 1% penicillin–streptomycin–l-glutamine (Corning, 30-009-CI). Cells were cultured at 37°C with 5% CO_2_ in a cell culture incubator. Mouse sarcoma cells were derived and maintained in cell culture from primary tumors following standard methods (3). For instance, tumor tissues were cut into small pieces, then digested by dissociation buffer containing collagenase type IV (Thermo Fisher Scientific, 17104-019), dispase (Thermo Fisher Scientific, 17105-041), and trypsin (Thermo Fisher Scientific, 25200056) for 1 hour in a shaker at 37°C (3). Cells were washed with 1× PBS (Thermo Fisher Scientific, SH30256LS) and filtered with a 40 µm sieve (Corning, 431750) (3).

### Plasmids.

To clone pX334–Negative sgRNA, pX334–Trp53 sgRNA, and pX334–Cdkn2a sgRNA, the pX334 vector was digested with BsaI enzyme and ligated to annealed sgRNA oligonucleotides (Supplemental Table 2) (11). For cloning of customized sgRNA libraries into these 3 vectors, a 35-gene targeting sgRNA library plus control sgRNAs were taken from the mouse Brie library (40). Oligonucleotide pools (Supplemental Table 5) were ordered from Twist Bioscience and cloned into the three pX334 vectors by Gibson assembly (New England Biolabs, E2611), as previously described (41). In brief, transformations of the assembled library were electroporated into Endura competent cells (Lucigen, 60242-1), and sgRNA representation was verified by high-throughput sequencing (11). Three additional genes (*Ano7*, *Csmd1*, and *Myt1l*) that are frequently mutated in myxofibrosarcoma were not included in oligonucleotide pools. Therefore, gene-targeting sgRNAs were cloned into pX334 vector, and those vectors were proportionally mixed with the library plasmids before injection into the mice (Supplemental Table 4). For cloning of individual candidate gene-targeting sgRNAs into the pX334 or pX333-Cre vectors, the vector was linearized by BbsI enzyme and ligated to annealed sgRNA oligonucleotides (Supplemental Table 4) (11).

### Tumor analysis.

IHC was performed on 5 μm sections of paraffin-embedded mouse tissues with the ABC kit (Vector Laboratories, PK-7200) with antibodies against MyoD (Agilent Technologies, M351201-2), myogenin (Agilent Technologies, IR06761-2), cytokeratin (Agilent Technologies, GA0536-2), smooth muscle actin (Agilent Technologies, GA61161-2), desmin (Agilent Technologies, GA63061-2), S100 (Agilent Technologies, GA50461-2), and Yap1 (Cell Signaling Technology, 4912S) (11). Tissue sections were stained with 3,30-diaminobenzidine and counterstained with hematoxylin (Sigma-Aldrich, H3136). H&E staining was performed using standard methods (11). All tissue sections were examined by a sarcoma pathologist following a blinded protocol (11).

### Genomic DNA isolation and indel analysis.

Genomic DNA was isolated by Zymo Quick-DNA Miniprep kit (Zymo Research, D3024) (11). PCR amplification of mouse targeted genes by specific sgRNAs was performed with specific primer (Supplemental Table 4) using Q5 HiFi DNA polymerase (New England Biolabs, M0494) (11). PCR was sent to Azenta for Sanger sequencing or next-generation sequencing.

### Western blot analysis.

NIH 3T3 and mouse sarcoma cell lines were treated with 0.5 μg/mL doxorubicin (Sigma-Aldrich, D1515-10MG) for 16 hours before sample harvest (11). Samples were lysed in RIPA buffer containing protease inhibitor cocktails (Sigma-Aldrich, R0278) for 30 minutes on ice, then centrifuged at 15,000*g* for 2 minutes (11). Samples were electrophoresed at 100 V for 60 minutes before transfer to PVDF (Thermo Fisher Scientific, IB24002) using an Iblot 2 Dry Blotting System (Thermo Fisher Scientific, IB21001) (11). The membrane was blocked in 5% nonfat dry milk in Tris-buffered saline (TBS; Corning, 46-012-CM) for 1 hour (11). Next, membranes were incubated with primary antibodies diluted in TBS-T (0.1% Tween 20) in a shaker overnight at 4°C: p53, 1:1,000 dilution (Cell Signaling Technology, 2524s); Rb1, 1:500 dilution (Santa Cruz Biotechnology, sc-102); Bap1, 1:1,000 dilution (Cell Signaling Technology, 13271s); and Gapdh, 1:100,000 dilution (Proteintech, 60004-1-lg) (11). Membranes were washed at least 3 times in TBS-T before incubation with Pierce HRP goat anti-rabbit secondary antibody (Fisher Scientific, PI31460) and Pierce HRP goat anti-mouse secondary antibody (Fisher Scientific, PI31430), both at 1:10,000 dilutions, in TBS-T for 1 hour at room temperature (11). The membranes were imaged using a Bio-Rad ChemiDoc imaging system (11). Full blots can be found in Supplemental Figure 14.

### Multiplex IHC and tissue array.

The staining procedure on mouse tissue slides was performed as per the protocol published from Earle A. Chiles Research Institute Immuno-Histology Core facility (48). We used 3% H_2_O_2_ to kill exogenous HRP when the antibody against a followed biomarker was raised from a different species of animal. Tissue sections were blocked with blocking/antibody diluent (ARD1001EA, Akoya) for 10 minutes at room temperature (48). Anti-CD68 (1:1,200; Cell Signaling Technology, E3O7V), anti–PD-1 (1:200; Cell Signaling Technology, D7D5W), anti-CD8 (1:400; eBioscience, 4SM15), anti-CD3 (1:100; Genetex, SP7), and anti-CD20 (1:6,400; Cell Signaling Technology, E3N7O) were sequentially stained (48). After a brief wash, tissue sections were incubated with MACH 2 Rb HRP-Polymer (Biocare Medical, RHRP520H) or rat HRP-Polymer (Biocare Medical, BRR4016 H) for 10 minutes at room temperature. After a quick wash, Opal690 (1:200; Akoya), Opal620 (1:200; Akoya), Opal570 (1:400; Akoya), and Opal520 (1:200; Akoya) were each applied for 10 minutes at room temperature, followed by Opal780 (1:25, Akoya) for 60 minutes at room temperature. DAPI staining (1 drop of DAPI solution into 0.5 mL of TBS-T, Akoya) was done for 5 minutes at room temperature (48). Then the slides were mounted with Prolong Diamond Antifade Mountant (Thermo Fisher Scientific, p36970). Imaging was performed on PhenoCycler-Fusion (48) (Akoya). For IHC in tissue array, anti-BAP1 (1:100; Santa Cruz Biotechnology, sc-28383) and anti-PLK1 (1:400; Proteintech, 10305-1-AP) antibodies were used on human sarcoma tissue array (Tissue Array Inc., SO2084 and SO2082b) using standard IHC protocol as previously described (76). Whole-slide imaging was performed using a ×20 objective lens, while high-power multispectral imaging was done using a ×40 objective lens. Images were analyzed using QuPath software (77).

### Flow cytometry.

Mouse sarcoma tumors and normal muscle were harvested, chopped finely, and collected directly into 2 mL digest buffer for 20–45 minutes on a rocker/shaker. The digestion buffer was prepared with 0.25 mg/mL Collagenase H (Sigma-Aldrich, catalog C8051) and 30 U/mL DNase I (Roche, catalog 04536282001) in complete DMEM (cDMEM). Single-cell suspension was collected after filtering and washing of the tumor digestions in 70 μm tube-top strainers (CellTreat, catalog 229483) with cDMEM. The single-cell suspensions were then stained with combinations of surface markers and intracellular targets (Supplemental Table 6) following the method of Rolig et al. (49). Flow cytometry data were acquired on an Aurora flow cytometer (Cytek Biosciences), and data were processed and analyzed with OMIQ software (Dotmatics) (49).

### Total RNA sequencing and analysis.

Human UPS RNA sequencing data are from the TCGA Sarcoma (TCGA-SARC) dataset, and human esophagus muscularis tissue and cultured fibroblasts from the Genotype-Tissue Expression (GTEx) project were used as controls (39, 78). Mouse sarcoma and normal muscle samples were preserved in RNAlater (Sigma-Aldrich, R0901-100ML). Then total RNA was isolated and prepared with an Illumina TruSeq Stranded Total RNA Library Prep kit. Analysis of RNA-seq data was performed using the workflow previously described (3). Log_2_ fold change values were shrunk using the approximate posterior estimation for generalized linear model (apeglm) method (3). Gene set enrichment analysis was performed on mouse hallmark gene sets from the Molecular Signatures Database (MSigDB) using the R package fgsea. Gene Ontology (GO) term enrichment was performed on biological process GO terms using the R package clusterProfiler. The raw data were deposited in the NCBI’s Sequence Read Archive (PRJNA1212052).

### Real-time qPCR.

cDNA was prepared using a LunaScript RT SuperMix kit purchased from New England Biolabs (E3010L). Quantitative reverse transcription PCR was performed using a QuantStudio 6 System (Applied Biosystems). RNA levels of Plk1 were quantified using a primer set (forward: CAGCAGCAGGAAACCTCTCA; reverse: CAGGATCCTCAGCCTCCTCT).

### In vitro CELLCYTE assay.

Mouse sarcoma cells were cultured in cDMEM as described above. Cells were inoculated into 96-well microtiter plates with 2,000 to 4,000 cells per well. The plates were incubated at 37°C for 24 hours before addition of experimental drugs. The experimental drugs were dissolved in DMSO and then further diluted with the volume in the desired maximum test concentration in cDMEM. Upon drug treatment, the plates were loaded in the CELLCYTE X live-cell imaging system (Cytena) and incubated at 37°C, 5% CO_2_, 95% air, and 100% relative humidity. The cells were monitored for 120 hours, and 2 images per well were acquired every 6 hours using the ×10 objective of the CELLCYTE X. A total of 21 scans were performed. The images were analyzed using the CELLCYTE Analysis software to generate cell confluence data. The experiments were performed in duplicate.

### Chemical compounds and antibodies used in in vitro and in vivo study.

Niraparib was purchased from TargetMol (T3231). Talazoparib was purchased from MedChemExpress (HY-16106). Volasertib was purchased from AdooQ Bioscience (A10135) or TargetMol (T6019). All compounds were dissolved in DMSO for in vitro study. For in vivo study, volasertib was resuspended in 0.5% methylcellulose. Anti-trinitrophenol isotype control rat monoclonal antibody (BE0089) and anti–mouse PD-1 (CD279) rat monoclonal antibody (RMP1-14) was purchased from Bio X Cell. Antibodies were freshly diluted in InVivoPure dilution buffer (IP0065), purchased from Bio X Cell.

### Targeted-capture sequencing.

The amplicon targeted-capture sequencing probes were synthesized by Azenta using the method previously described (38). The capture sequencing was done by Azenta. The raw data were deposited in the Sequence Read Archive (PRJNA1212052).

### Significantly mutated genes.

Mutect2 and VarScan were used to call mutations (79, 80). Mutect2 mutations must have at least 1,000 reads and a variant frequency of at least 5%. VarScan mutations must have a total of 100 reads and a variant frequency of at least 10%. Filtered mutations used in downstream analyses were Mutect2 mutations that either were flagged as “PASS” or were VarScan mutations as well. Furthermore, these filtered mutations must be located within the probe-targeted regions. Annotations of Mutect2 mutations were done via SnpEff (81).

### In vivo electroporation.

All in vivo electroporation was performed by a method previously described (11). Mouse fur was shaved at least 1 day before the experiment. Then, 50 μg of endotoxin-free DNA plasmids in 50 μL sterile saline was intramuscularly injected into the mice using a 28-gauge syringe (11). Next, a pair of needle electrodes was inserted into the muscle at the injection site, and electric pulses were delivered using a BTX ECM830 device (Harvard Apparatus, 45-2052) (11). sgRNAs used in the individual gene validation experiments were all from the customized sgRNA library listed in Supplemental Table 2, except Trp53 sgRNA (GTGTAATAGCTCCTGCATGG), negative control sgRNA (GCGAGGTATTCGGCTCCGCG), Cdkn2a sgRNA (GGGCCGCCCACTCCAAGAGA), and Pten sgRNA (GCTAACGATCTCTTTGATGA). To mutate Trp53 and Rb1 in the Rosa26^loxP-Cas9^ mice, we delivered a mixed plasmid pool of pX333-Trp53-Rb1 sgRNA1 (AAATGATACGAGGATTATCG), pX333-Trp53-Rb1 sgRNA2 (AGAGAAGTTTGCTAACGCTG), pX333-Trp53-Rb1 sgRNA3 (TAAGTACGTTCAGAATCCAC), and pX333-Trp53-Rb1 sgRNA4 (GCAGTATGGTTACCCTGGAG). We also generated tumors in mice with similar tumor onset by delivering a single plasmid of pX333-Trp53-Rb1 sgRNA4. To mutate Trp53 and Pten in the Rosa26^loxP-Cas9^ mice, we delivered a plasmid of pX333-Trp53-Pten sgRNA. To mutate Trp53 and Bap1 in the Rosa26^loxP-Cas9^ mice, we delivered a mixed plasmid pool of pX333-Trp53-Bap1 sgRNA1 (CCACCAACGTAGAAACCTTG) and pX333-Trp53-Bap1 sgRNA4 (TCAGCTATGTGCCTATCACA). We also generated tumors in mice with similar tumor onset by delivering a single plasmid of pX333-Trp53-Bap1 sgRNA4. To mutate Trp53 and Crebbp in the mice, we delivered a mixed plasmid pool of pX333-Trp53-Crebbp sgRNA3 (TAATGAATCAGGCTCAACAA) and pX333-Trp53-Crebbp sgRNA4 (TGAACCTACTGAATCCAAGG). To mutate Trp53 and Mst1r in the mice, we delivered a mixed plasmid pool of pX333-Trp53-Mst1r sgRNA1 (AAGTATCAGACTTTAGACGA) and pX333-Trp53-Mst1r sgRNA2 (GGGAACACACCAGATCACCG). To mutate Trp53 and Pbrm1 in the mice, we delivered a mixed plasmid pool of pX333-Trp53-Pbrm1 sgRNA1 (AAAACACTTGCATAACGATG) and pX333-Trp53-Pbrm1 sgRNA3 (AATAAAAGAGCAGTCCAAGG). To mutate Trp53 and Cysltr2 in the mice, we delivered a mixed plasmid pool of pX333-Trp53-Cylstr2 sgRNA1 (AAACCCATATGATCCCACAG) and pX333-Trp53-Cysltr2 sgRNA4 (GATGAATAGAAAATCGGAAG). To mutate Trp53 and Mllt3 in the mice, we delivered a mixed plasmid pool of pX333-Trp53-Mllt3 sgRNA3 (TCCACGATGTCATCAAACGG) and pX333-Trp53-Mllt3 sgRNA4 (ACTTACTCACCGTCACCAGT). To delete Trp53 and Rb1 in the Trp53^fl/fl^ Rb1^fl/fl^ Rosa26^loxP-Cas9/loxP-Cas9^ mice, we delivered the pX333 plasmid to the mice. To delete Trp53, Rb1, and Bap1 in the Trp53^fl/fl^ Rb1^fl/fl^ Rosa26^loxP-Cas9/loxP-Cas9^ mice, we delivered a mixed plasmid pool of pX333-Trp53-Bap1 sgRNA1 and pX333-Trp53-Bap1 sgRNA4.

### Statistics.

Data are expressed as mean ± SEM unless noted otherwise. Before tests were performed, datasets were visualized to assess distribution and determine whether parametric or non-parametric methods were appropriate. Comparisons between 2 groups were evaluated using a 2-tailed Student’s *t* test. For unpaired samples requiring non-parametric analysis, a 1-sided Wilcoxon’s rank-sum test was applied. Tumor-free survival was analyzed using Kaplan-Meier curves, and statistical significance was assessed with the log-rank test. *P* values less than 0.05 were considered significant. All analyses were conducted using GraphPad Prism version 10.

### Study approval.

All animal studies were performed in accordance with protocols approved by the Animal Care and Use Committee of the Earle A. Chiles Research Institute.

### Data availability.

Supporting data values underlying the figures are provided in the Supporting Data Values XLS file. Bulk RNA sequencing data and targeted-capture sequencing raw data generated in this study were deposited in the NCBI’s BioProject under accession number PRJNA1212052. All other data supporting the findings of this study are available within the article and its supplemental materials or upon reasonable request.

## Author contributions

JH and DGK designed experiments. JH, XL, WH, ZS, MJK, WF, RP, SYK, ESX, LL, YM, and ZZ performed experiments. WH and YM performed IHC. ZS performed multiplex IHC. JH, XL, and MJK performed flow cytometry. YW examined histology of mouse sarcomas. BP, JTW, VR, WKR, and BB performed and analyzed deep sequencing. RBB, WLR, and WJU provided critical advice for the manuscript. JH, WJU, and DGK drafted the first version of the manuscript. All authors edited the manuscript.

## Conflict of interest

WLR has received research support from Inhibrx, Shimadzu, AstraZeneca, and ImmunAI. WLR receives licensing fees from Galectin Therapeutics and is a coinventor on patent US9872909B2. WLR is on the Scientific Advisory Board at Medicenna and Vesselon. DGK is a coinventor on a patent for a handheld imaging device and is a coinventor on a patent for radiosensitizers (patent name: System and method for large field of view, single cell analysis; US patent number: US 11730371; patent name: Dual ATM and DNA-PK inhibitors for use in anti-tumor therapy; US Patent Number: US 12187742). Merck provided research support to DGK in the past.

## Funding support

This work is the result of NIH funding, in whole or in part, and is subject to the NIH Public Access Policy. Through acceptance of this federal funding, the NIH has been given a right to make the work publicly available in PubMed Central.

- National Cancer Institute of the NIH under award K22CA248849 (to JH).
- Sarcoma Foundation of America grant (to JH).
- Northwestern Sarcoma Foundation (to JH).
- NIH R35CA197616 (to DGK).
- Providence Portland Medical Foundation (to JH).

## Supplementary Material

Supplemental data

Unedited blot and gel images

Supporting data values

## Version 1 06/30/2026

In-Press Preview

## Version 2 08/24/2026

Electronic publication
