# Supplementary material for: A tailored in vivo CRISPR screen identifies BAP1 as a potent tumor suppressor of sarcoma
Source: JCI Insight. 2026 Jun 30;11(16):e192686. doi: 10.1172/jci.insight.192686 (PMC13502177; doi:10.1172/jci.insight.192686)
Supplement: Supplementary file 1 [file jciinsight-11-192686-s120.pdf]

Title: A tailored *in vivo* CRISPR screen identifies *BAP1* as potent tumor suppressors of sarcoma

Authorship: Jianguo Huang [1*], Xingliang Liu [1], Warren Floyd [2#], William Haugh [1], Zhaoyu Sun [1], Melissa J. Kasiewicz [1], Yaping Wu [1], Brian Piening [1], John T. Welle [1], Wesley K. Rosales [1], Venkatesh Rajamanickam [1], So Young Kim [3], Eric Xu [2], Lixia Luo [2], Yan Ma [2], Rutulkumar Patel [2$], Ziqiang Zhang [4], Brady Bernard [1], William L. Redmond [1], Walter J. Urba [1], R Bryan Bell, David G. Kirsch [2,5,6,7,8*]

[*] Equal contribution, co-corresponding authors

[1] Earle A. Chiles Research Institute, Providence Cancer Institute, Portland, OR 97213

[2] Department of Radiation Oncology, Duke University Medical Center, Durham, NC 27710

[3] Department of Molecular Genetics and Microbiology, Duke University, Durham, NC 27710

[4] Department of Respiratory and Critical Care Medicine, Shanghai Pudong Hospital, Fudan University Pudong Medical Center, Pudong Hospital of Fudan University, Shanghai, China

[5] Department of Pharmacology and Cancer Biology, Duke University Medical Center, Durham, NC 27710

[6] Radiation Medicine Program, Princess Margaret Cancer Center, University Health Network, Toronto, ON, Canada

[7] Department of Radiation Oncology, University of Toronto, Toronto, ON, Canada

[8] Department of Medical Biophysics, University of Toronto, Toronto, ON, Canada

[#] Current position: Division of Radiation Oncology, University of Texas MD Anderson Cancer Center, Houston, TX 77030

[$] Department of Radiation Oncology, Baylor College of Medicine, Houston, TX 77030

[*] Corresponding authors: Jianguo Huang, Earle A. Chiles Research Institute, Providence Cancer Institute, Portland, OR 97213 (Email: Jianguo.huang@providence.org), and David G. Kirsch, Department of Radiation Oncology, Radiation Medicine Program, University of Toronto, Princess Margaret Cancer Center, Toronto, ON, Canada (Email: david.kirsch@uhn.ca)

Supplementary Figure 1. Copy numbers of each sgRNA in the three libraries by sequencing.

A

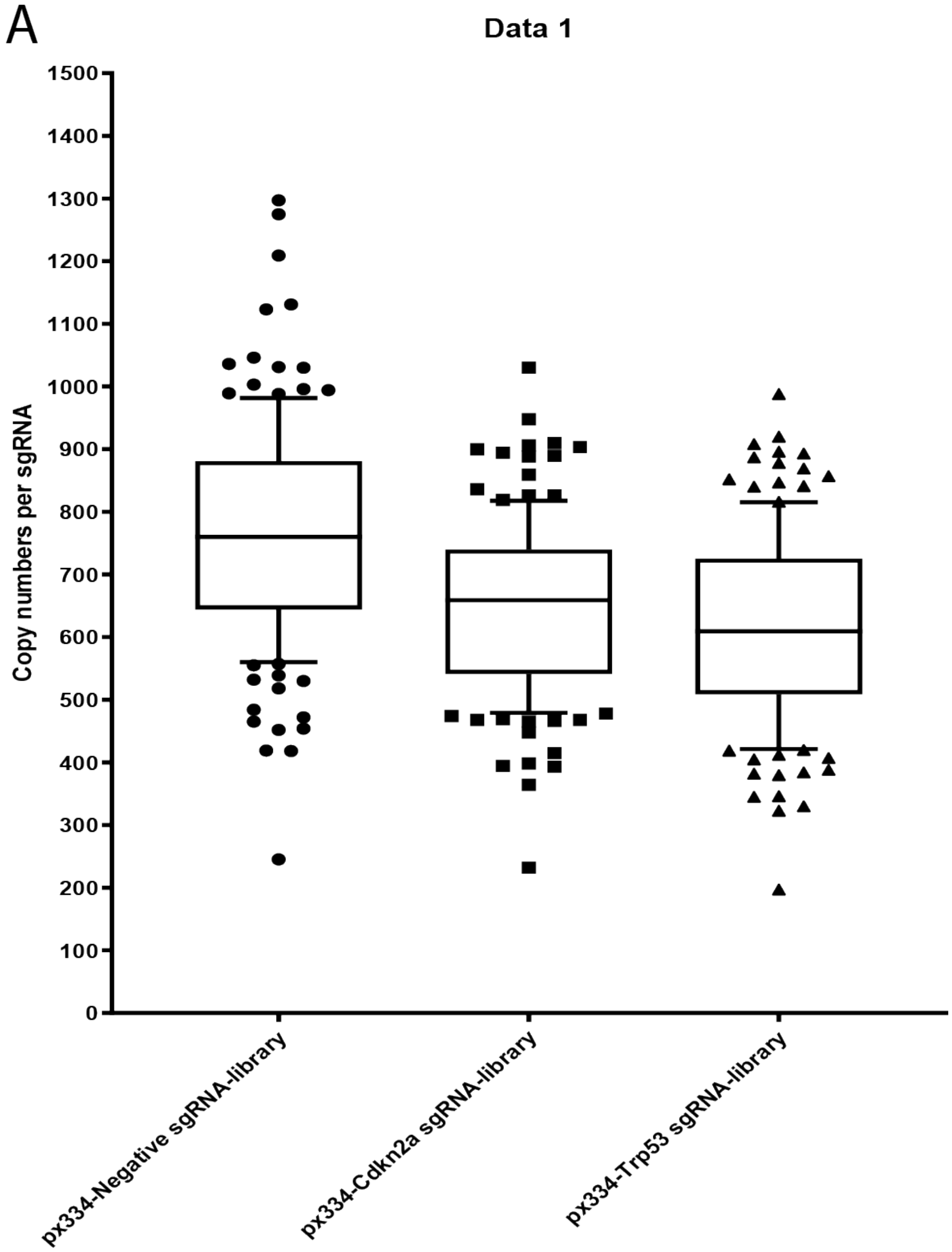

Supplementary Figure 2. Tumor free survival in Rosa26 $^{LoxP\text{-}Cas9/+}$ mice following IVE delivery of three different libraries. 5 mice per group.

A

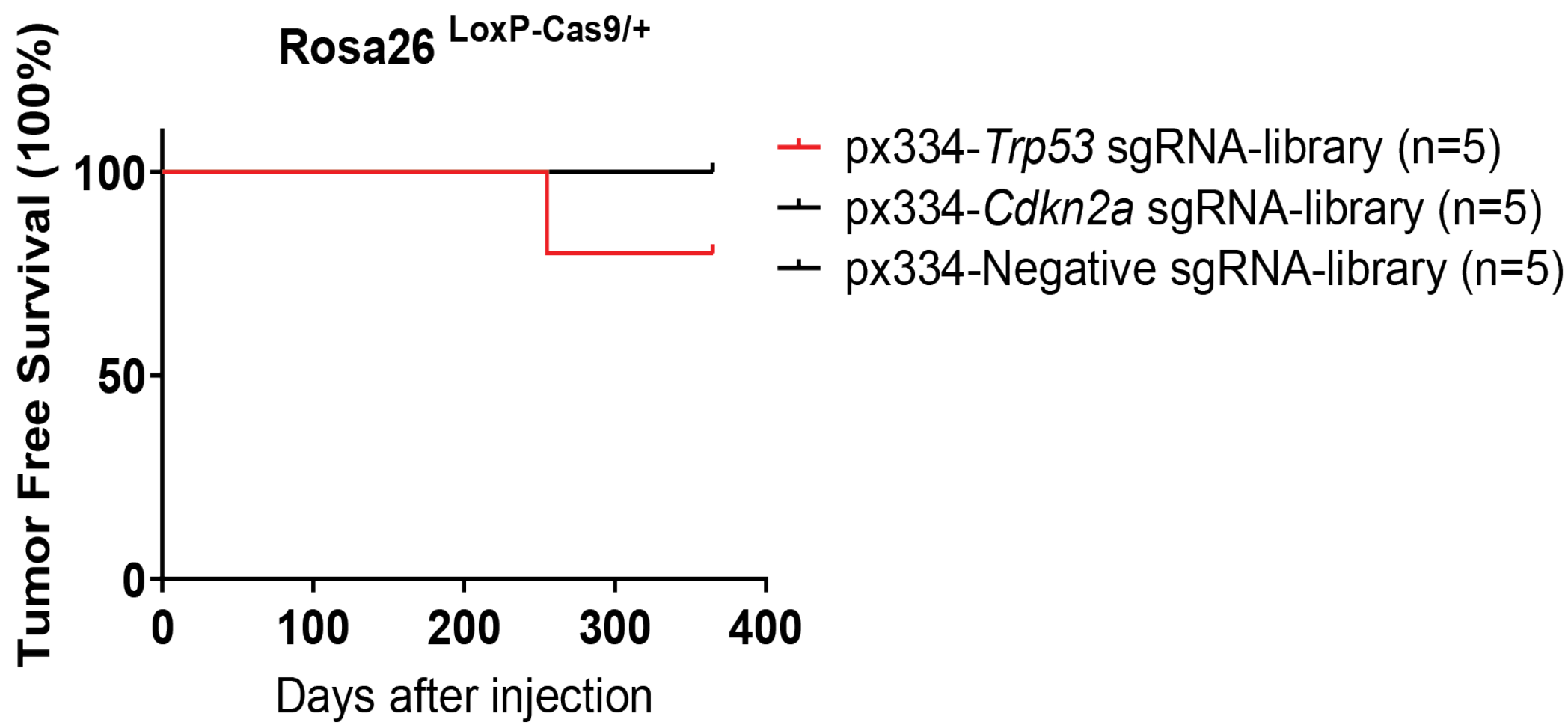

Supplementary Figure 3. (A) The Rosa26 $^{LoxP\text{-}Cas9/LoxP\text{-}Cas9}$ mice were intramuscularly injected (IVE) with 50 µg of mixed plasmids containing px333-Trp53-Pten sgRNAs and the px334-Trp53 sgRNA-library at a 1-1 ratio.

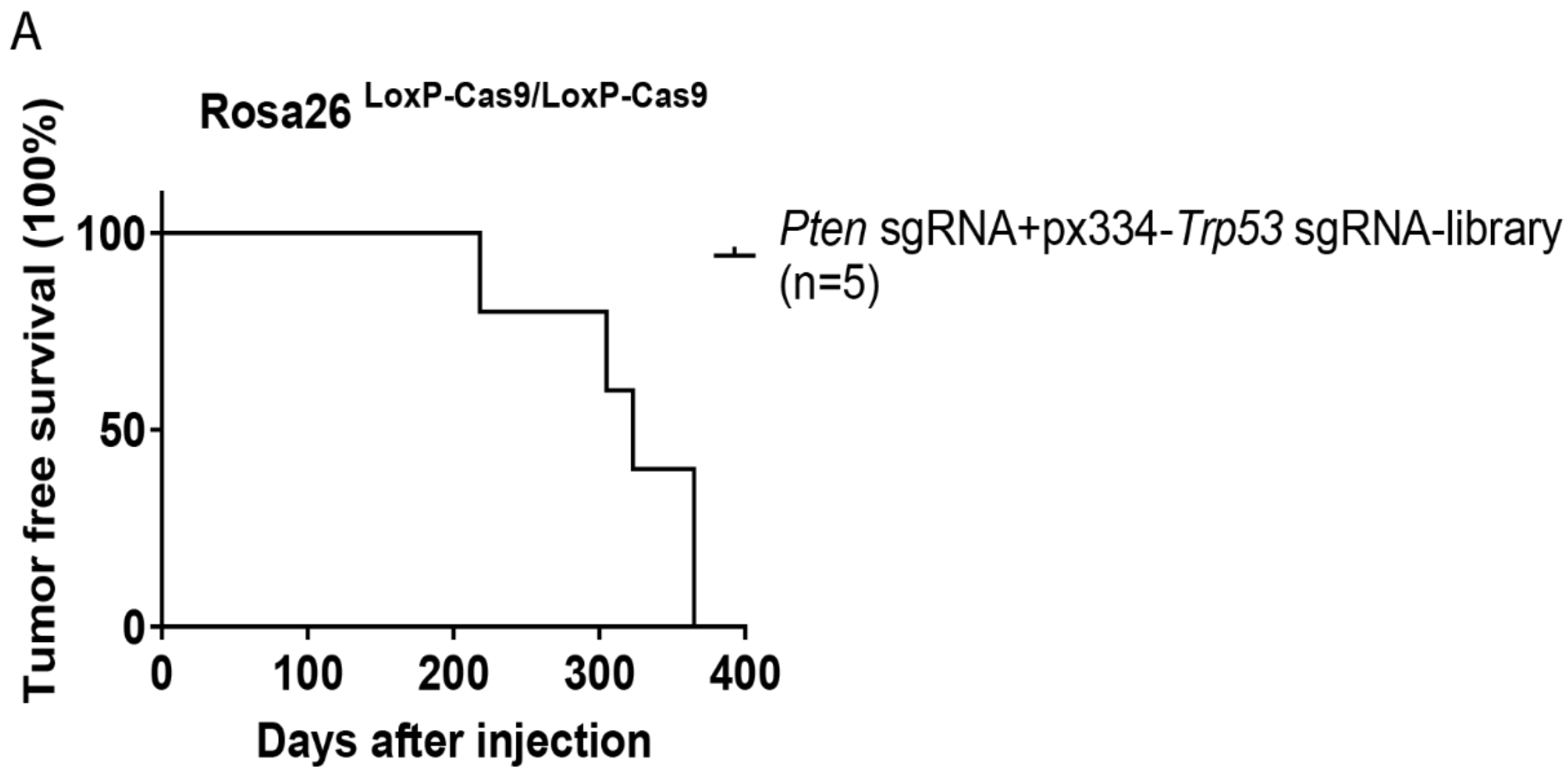

Supplementary Figure 4. (A) T7EI assay was performed to detect Indels in tumor DNAs induced by the px334-Trp53 sgRNA-library. (B) Sanger sequencing with Inference of CRISPR Edits (ICE) analysis was used to calculate the percentage of Indels in tumor S1 induced by the pX334-Trp53 sgRNA-library. (C) Next generation sequencing analysis was used to calculate the percentage of Indels in tumor S7, S11, and S13 induced by the pX334-Trp53 sgRNA-library.

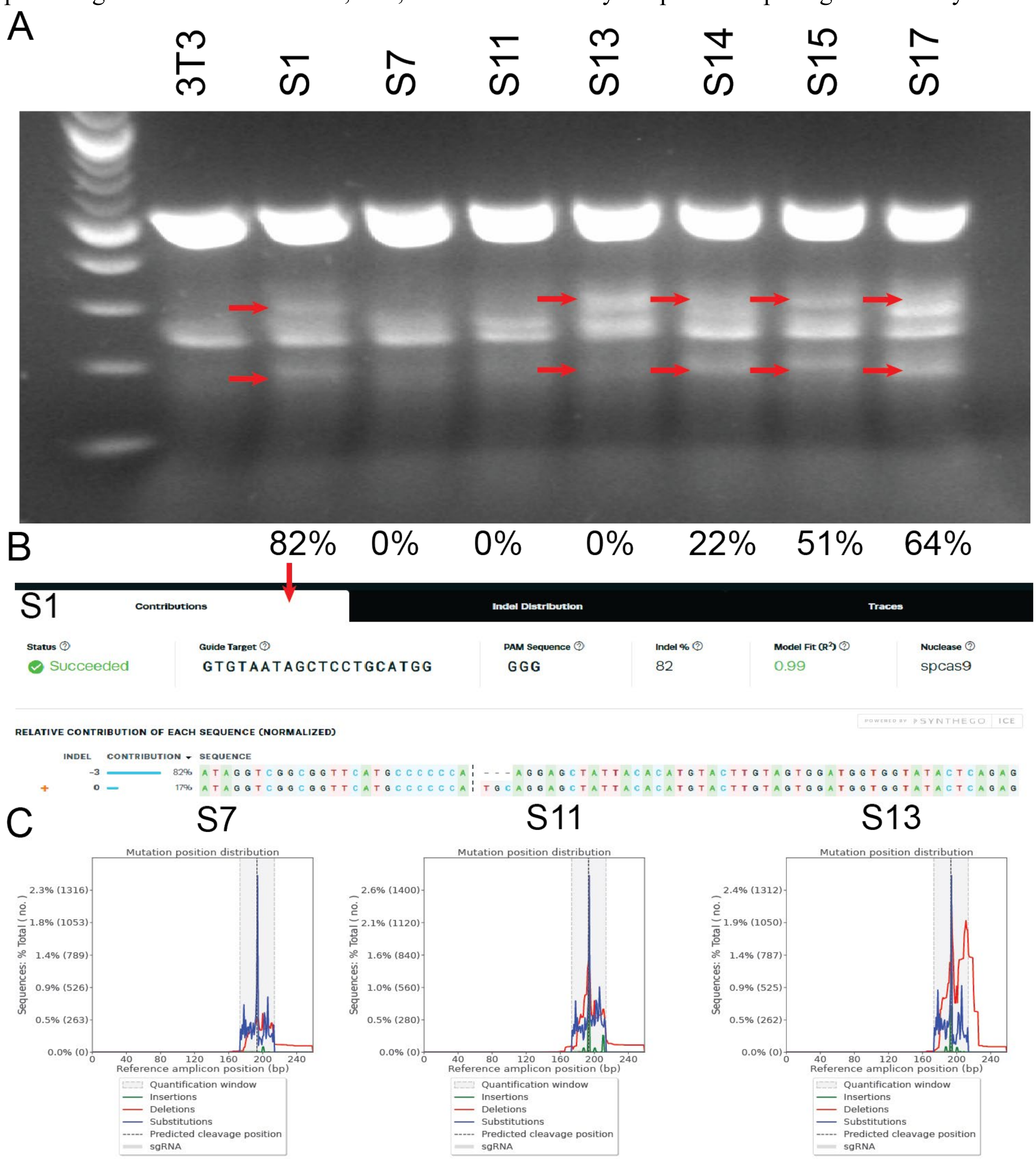

Supplementary Figure 5. No tumors were detected in mice IVE of plasmids containing either Trp53-Mllt3 sgRNAs, Trp53-Crebbp sgRNAs, Trp53-Pbrm1 sgRNAs, Trp53-Cysltr2, Trp53-Mst1r, or Trp53-Fat1 sgRNAs.

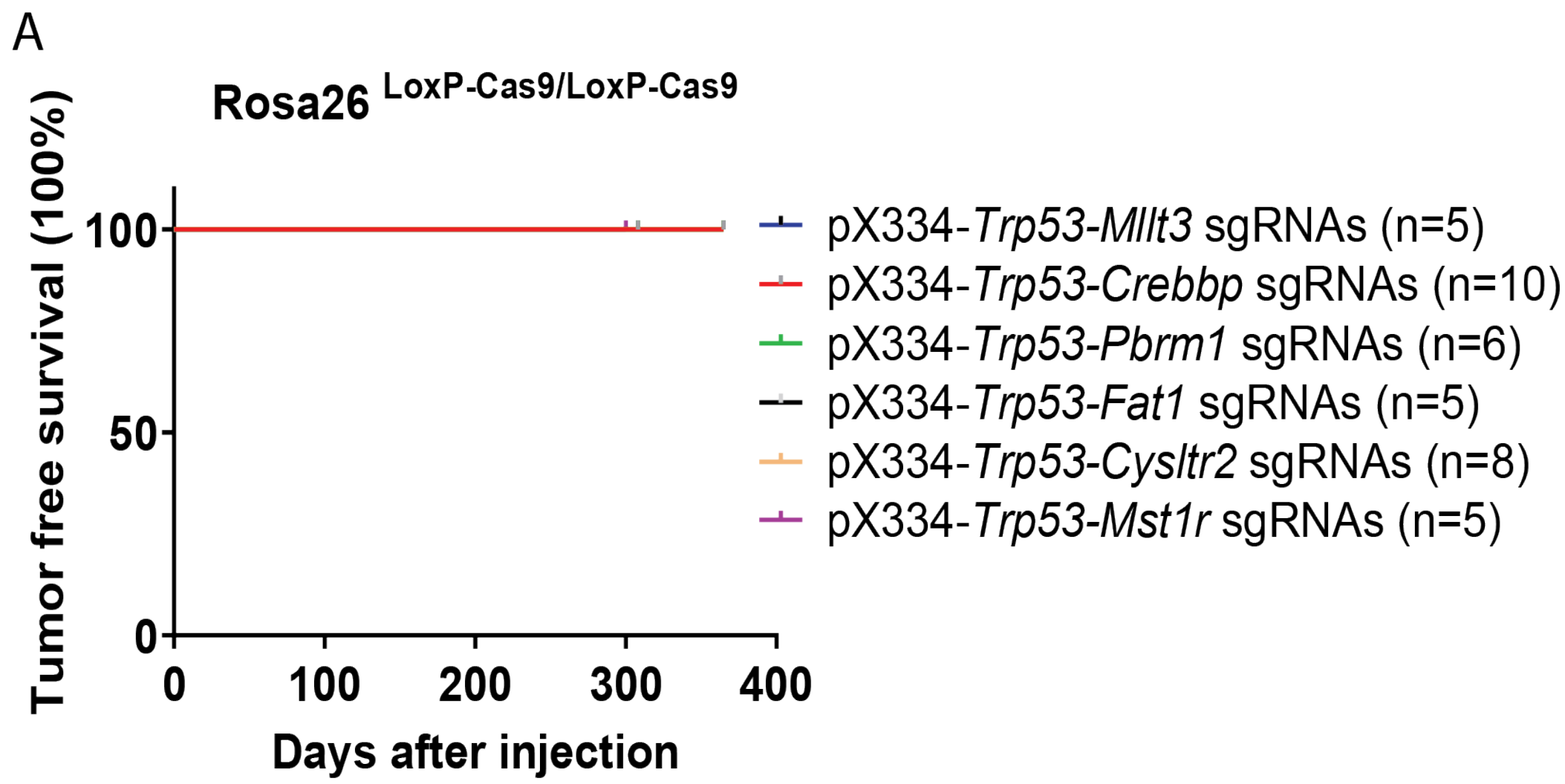

Supplementary Figure 6. H&E staining of mouse tumors driven by various gene mutations shows that these tumors are similar to each other. Scale bar = 50 µm.

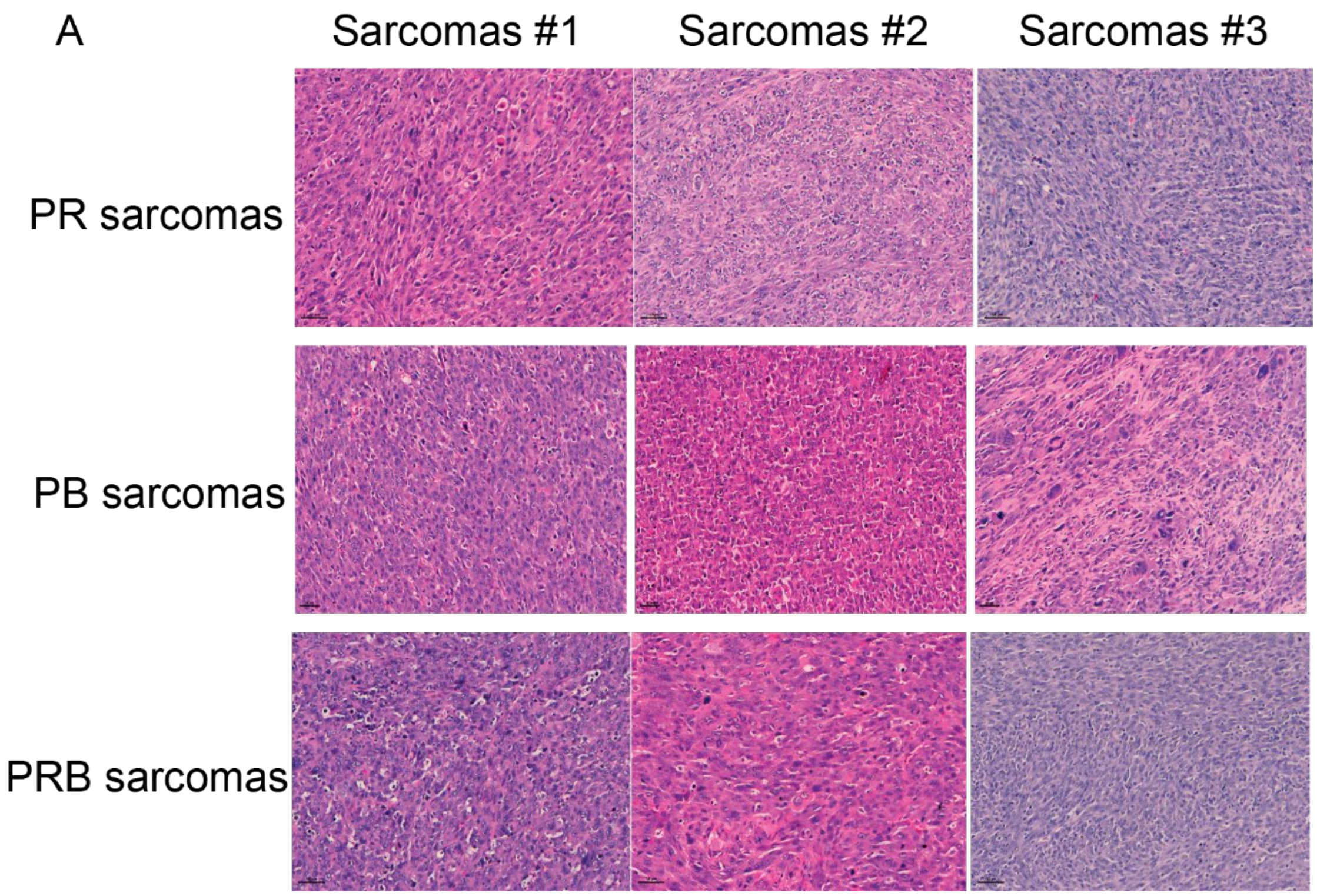

Supplementary Figure 7. Immunohistochemistry with CD68 in various mouse sarcoma tissue sections showed abundant $CD68^+$ macrophages in the tumor. Scale bar = 100 μm.

A

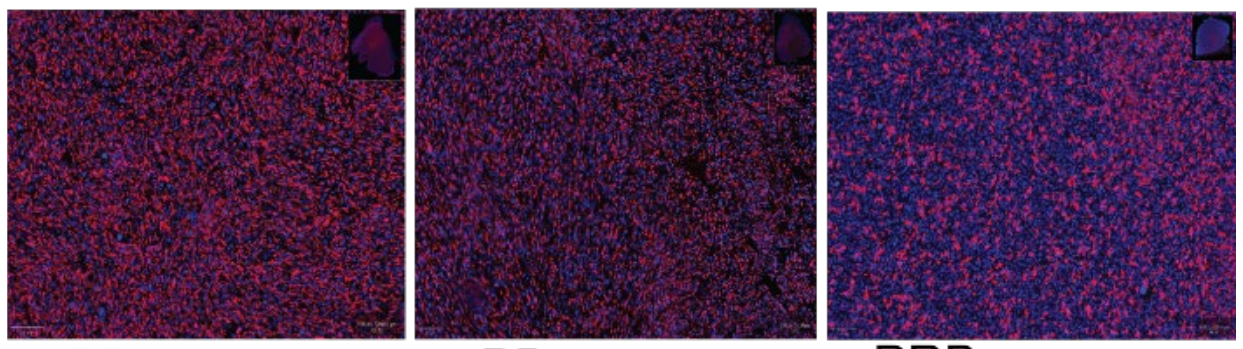

PR sarcoma PB sarcoma PRB sarcoma

Supplementary Figure 8. (A) FC showed that there are no differences in the percentage of PD-$1^{+}CD8^{+}$ T cells between PR CRISPR large, PB CRISPR large and small tumors.

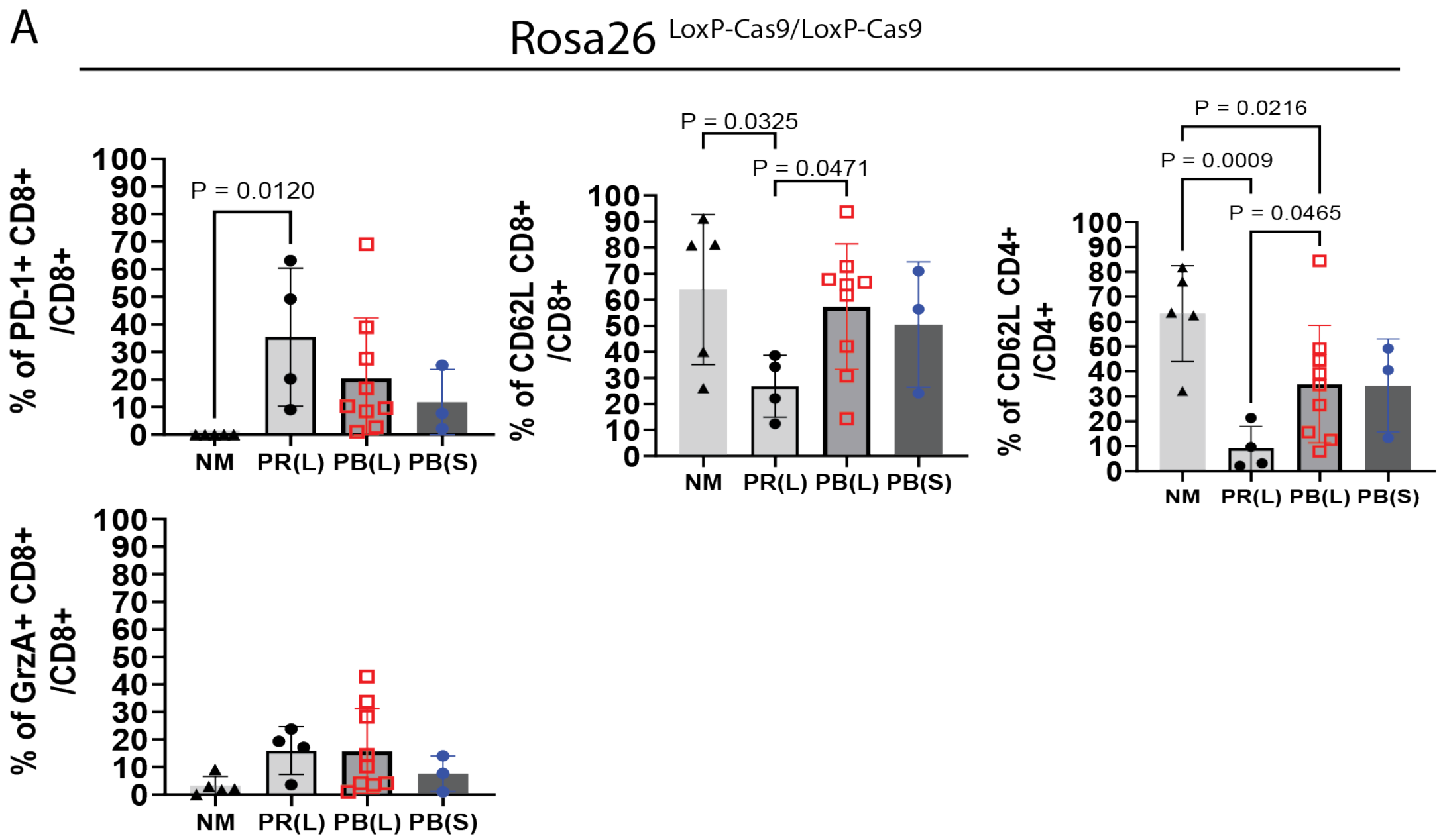

Supplementary Figure 9. (A) There are no differences in the percentage of CD11b$^+$ cells between PR CRISPR large, and PB CRISPR large tumors. However, the percentage of CD11b$^+$ cells is significantly lower in PB CRISPR early tumors compared to PB CRISPR large tumors. (B) Flow cytometry gating strategy to identify PMN-MDSCs, M-MDSCs, and TAMs. (C) There are no differences in the percentage of PMN-MDSCs, M-MDSCs, or TAMs between PB CRISPR large and small tumors. (D) There is a significantly low percentage of PD-L1$^+$ M-MDSCs in PRB Cre/CRISPR tumors compared to PR Cre tumors.

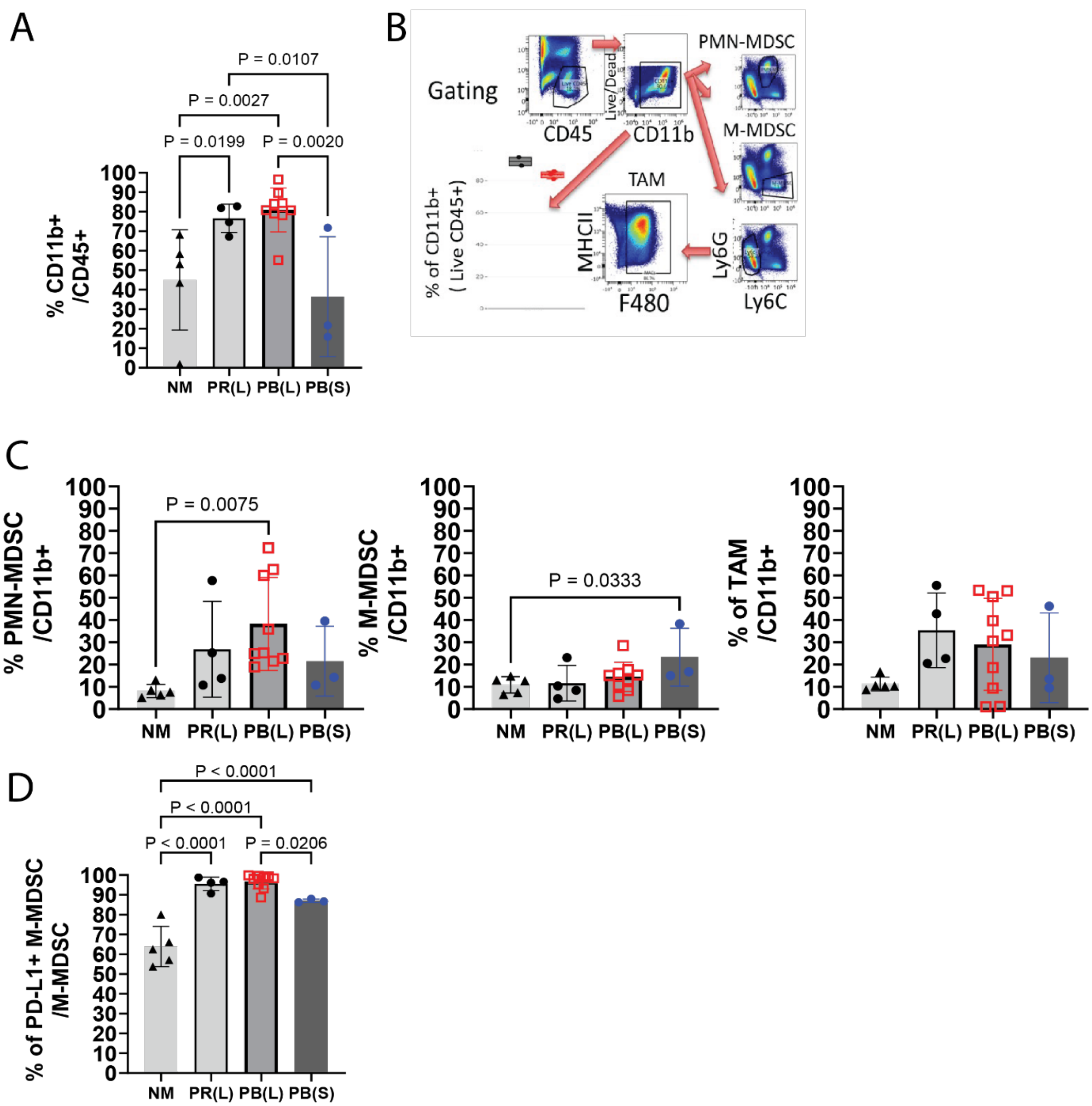

Supplementary Figure 10. (A) Western blot analysis to determine the knockout of Trp53 and Rb1 in PR cell lines. (B) Western blot analysis to determine the knockout of Trp53 and Bap1 in PB cell lines.

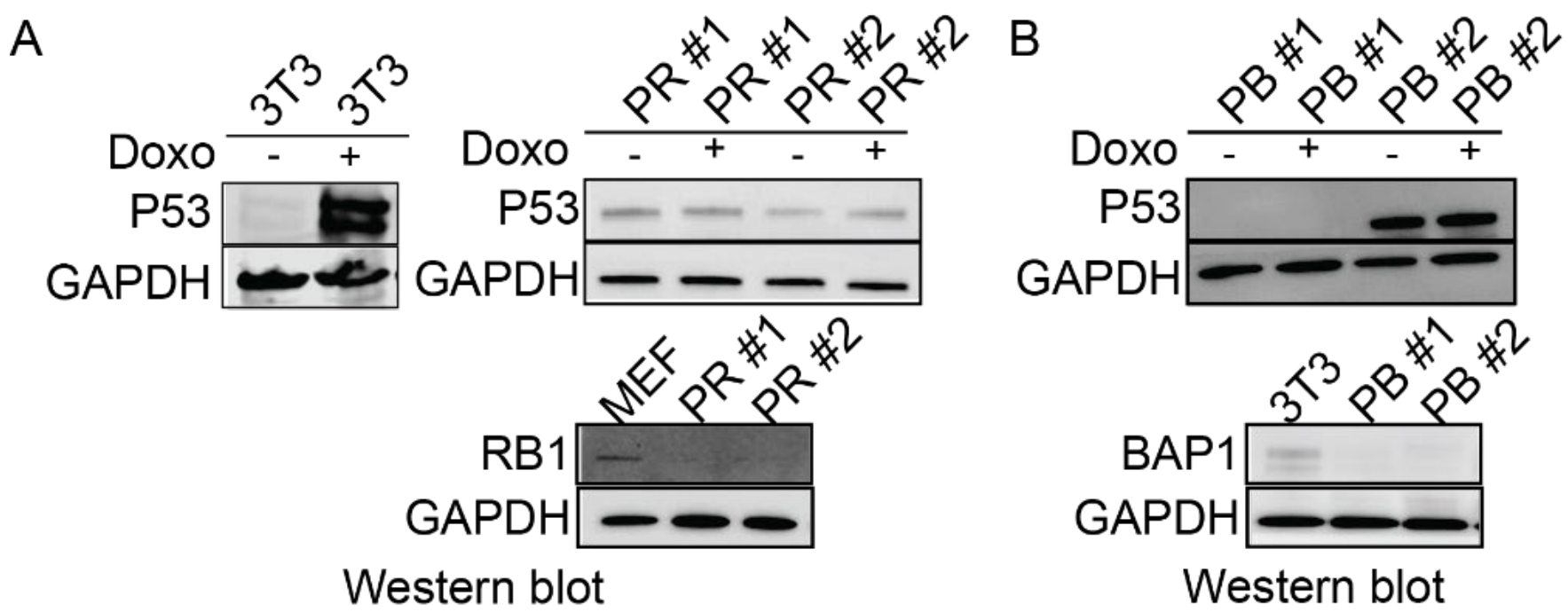

Supplementary Figure 11. (A) Both PR and PB mouse sarcoma cells are sensitive to two PARP inhibitors, niraparib and talazoparib, in a dose-dependent manner in vitro. (B) Niraparib significantly slows PR sarcoma growth in subcutaneous nude mice, while niraparib has no impact on PB sarcoma growth in subcutaneous nude mice. 5 mice in each group.

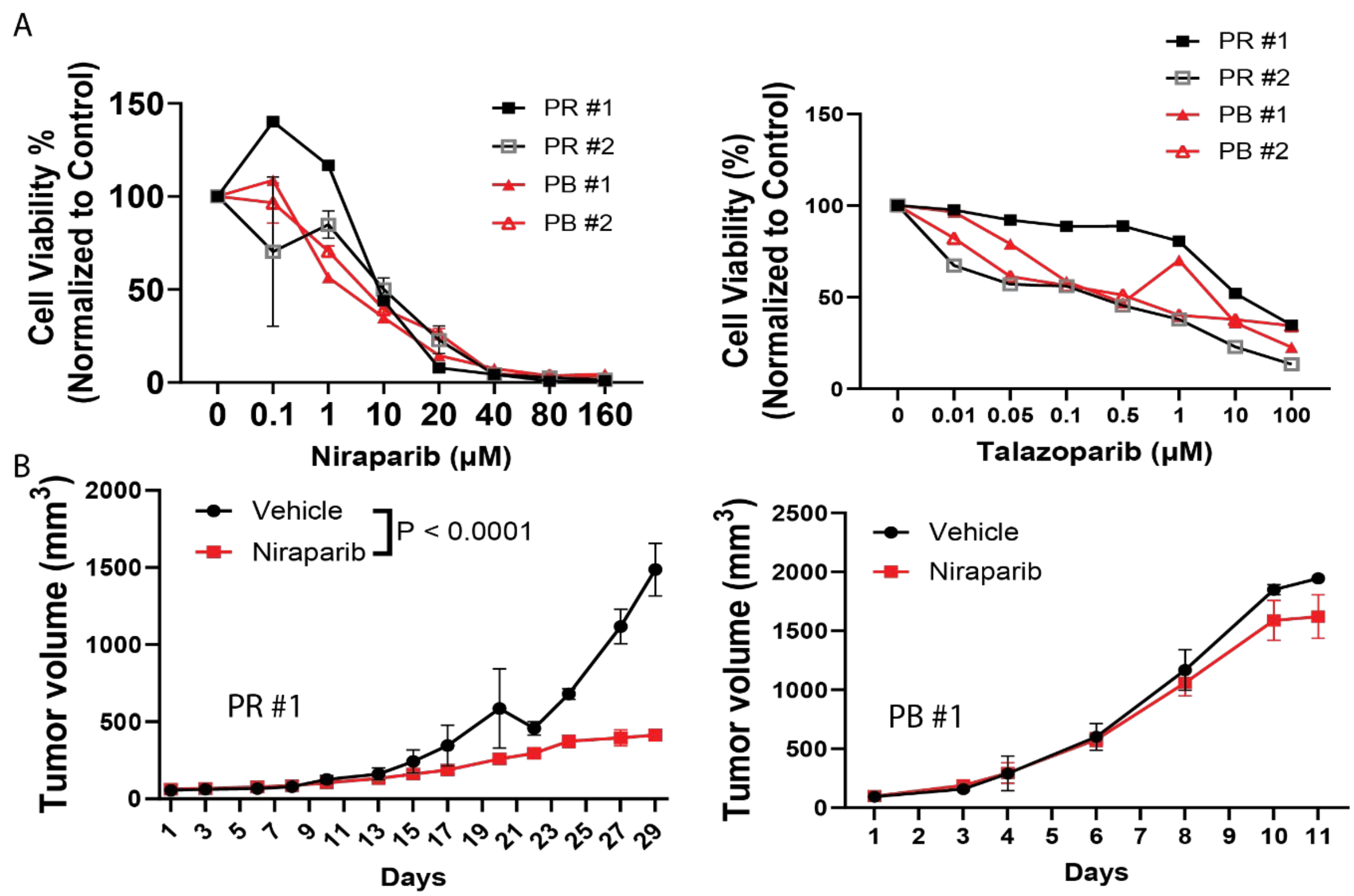

Supplementary Figure 12. There are no differences in the percentage of CD11b$^+$ cells between vehicle and volasertib treatment group.

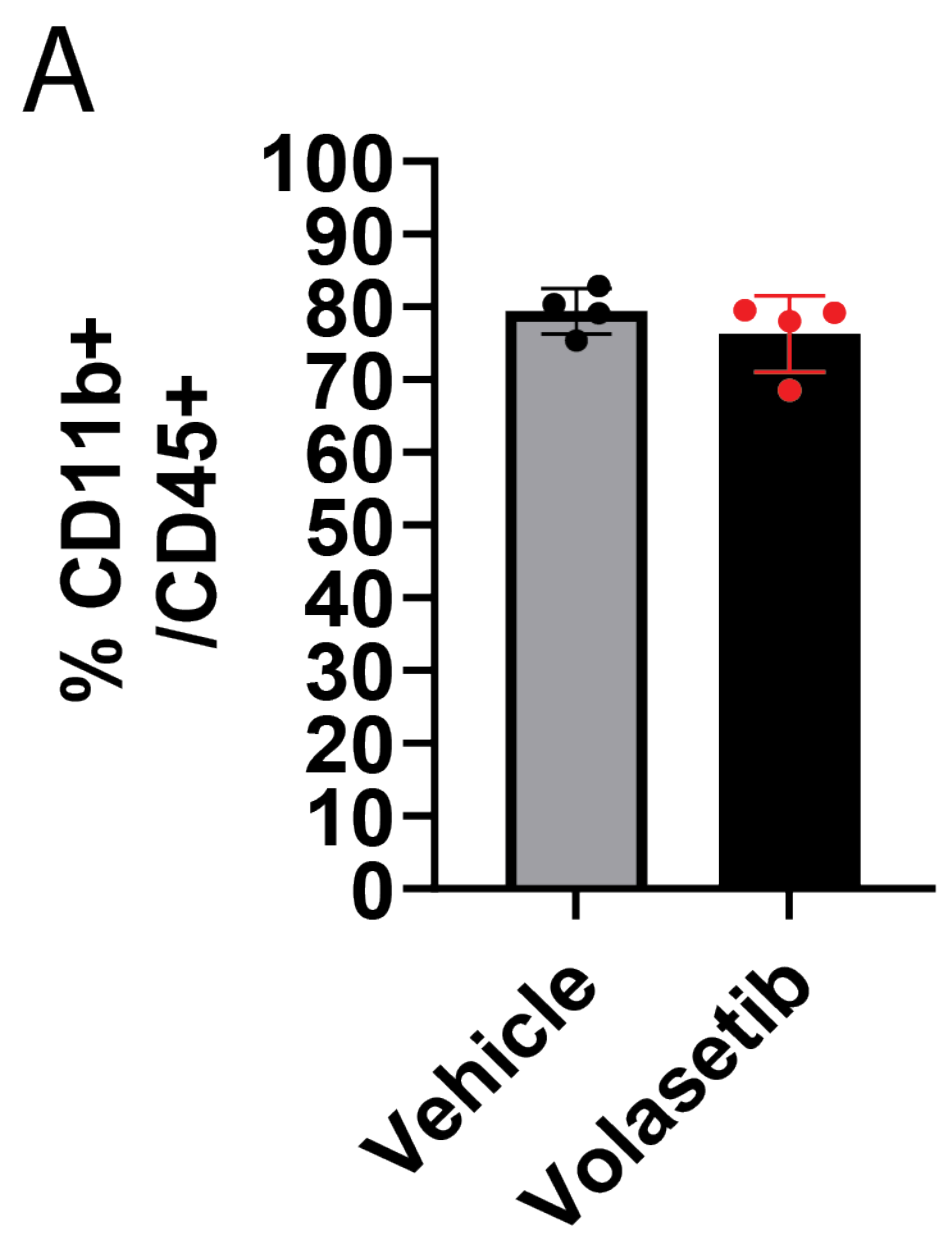

Supplementary Figure 13. Mutation profiling of PR-DUB complex genes in human UPS according to the TCGA database.

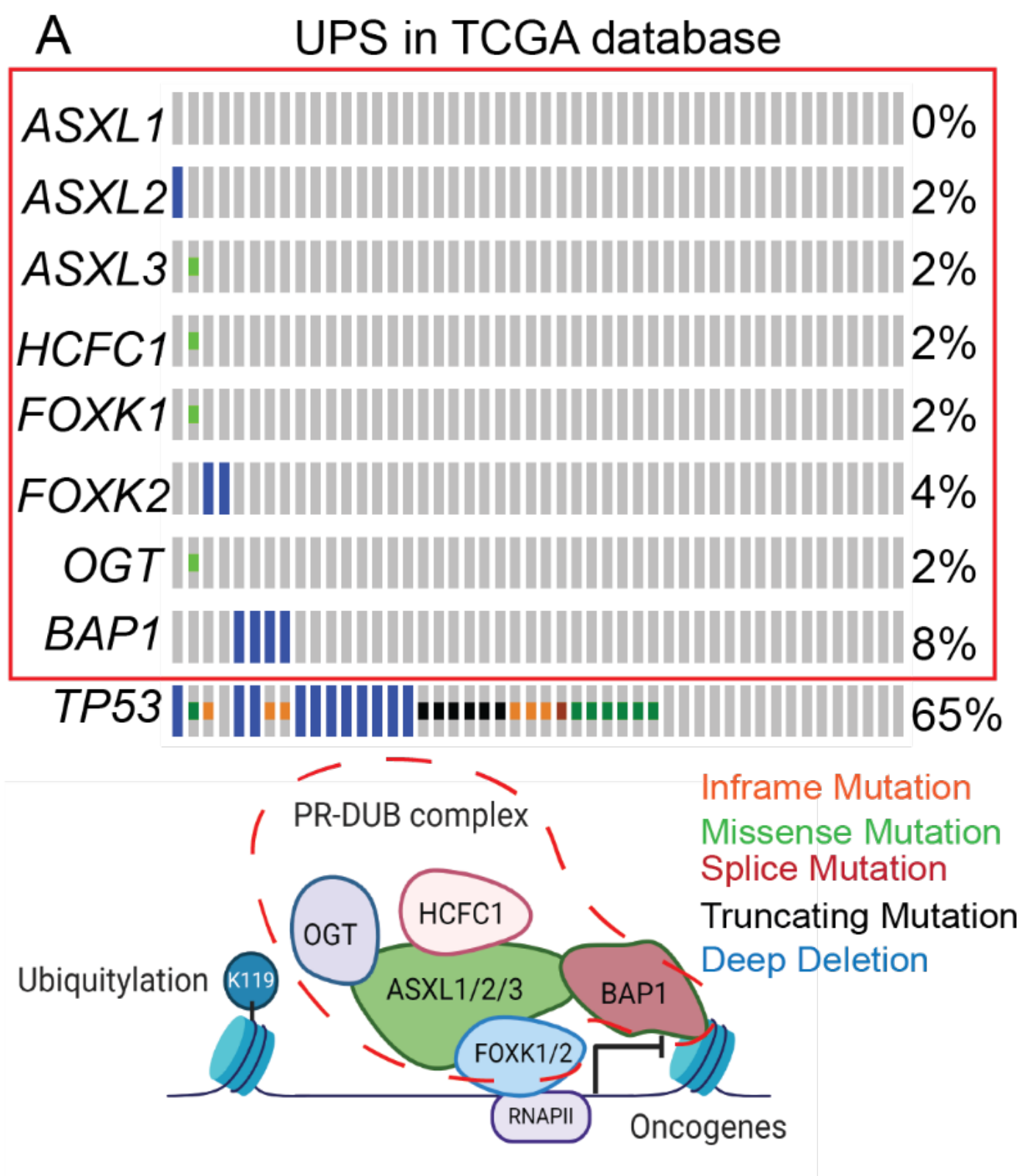

Supplementary Figure 14. Raw images of all western blotting are presented in this manuscript.

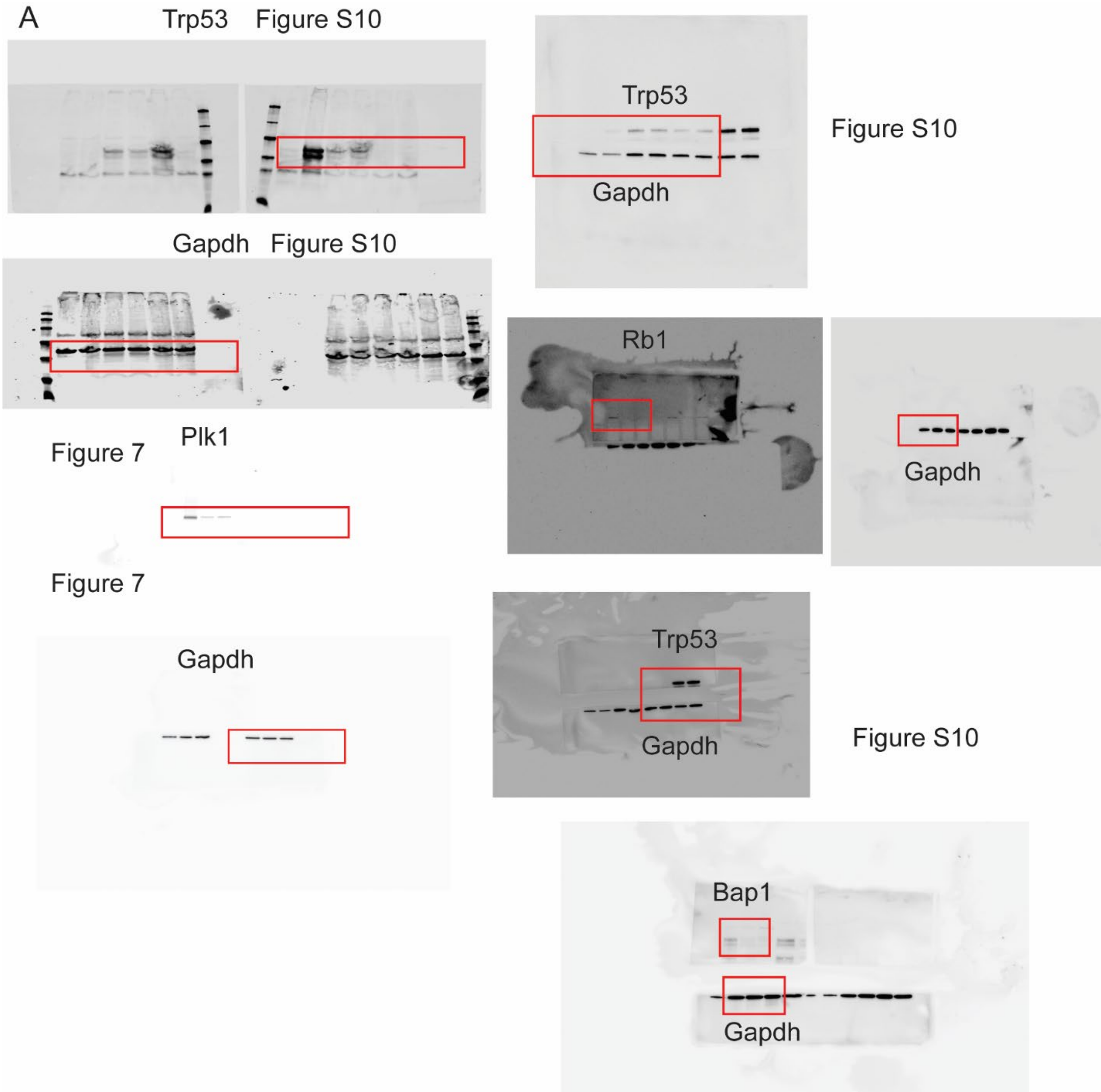

Supplementary Table 1. Top 35 genes plus TP53 and CDKN2A that are loss-of-function mutated or have copy number alterations in human UPSs and MFSs.

Gene		Copy Change	CN deletion TCGA	TCGA driver mut	CN deletion Genie	GENIE Frameshift mutations	GENIE other Driver mutations	Total # of samples with gene Alterations	Percentage of samples with Alterations	Total Number of UPS+MFS Samples
TP53	17p13.1	DEL	15	20	10	9	15	69	0.56097561	123
RB1	13q14.2	DEL	16	5	12	6	1	40	0.32520325	123
CYSLTR2	13q14.2	DEL	13					13	0.10569106	123
CDKN2A	9p21	DEL	14	1	10	3	0	28	0.22764228	123
ATRX	Xq21.1	DEL	3	16	1	7	0	27	0.2195122	123
D2HGDH	2q37.3	DEL	10					10	0.08130081	123
SNED1	2q37.3	DEL	10					10	0.08130081	123
RHOA	3p21.3	DEL	4	14	1	0	0	19	0.15447154	123
CDKN2B	9p21	DEL	14	0	9	0	0	23	0.18699187	123
MTAP	9p21	DEL	8					8	0.06504065	123
HDAC4	2q37.3	DEL	8					8	0.06504065	123
PASK	2q37.3	DEL	7					7	0.05691057	123
ACKR3	2q37.3	DEL	7					7	0.05691057	123
ANO7			7					7	0.04605263	152
FAT1	4q35	DEL	6	3	1	1	0	11	0.08943089	123
PDCD1	2q37.3	DEL	11					11	0.08943089	123
WWOX	16q23	DEL	5					5	0.04065041	123
DNAH12	3p14.3	DEL	4					4	0.03252033	123
FHIT	3p14.2	DEL	4					4	0.03252033	123
INPP5D	2q37.1	DEL	4					4	0.03252033	123
MLLT3	9p22	DEL	4					4	0.03252033	123
RYR1			0	4	0	0	0	4	0.03252033	123
NUMBL			1	3	0	0	0	4	0.03252033	123
PARP3	3p21.2	DEL	4					4	0.03252033	123
MST1R	3p21.3	DEL	4		2	0	0	6	0.04878049	123
CSDMD1			4					4	0.02631579	152
MST1	3p21	DEL	4		1	0	0	5	0.04065041	123
KDM4C	9p24.1	DEL	3					3	0.02439024	123
PSIP1	9p22.3	DEL	3					3	0.02439024	123
MYT1L			2	1				3	0.01973684	152
PBRM1	3p21	DEL	4		1	0	0	5	0.04065041	123
BAP1	3p21.1	DEL	4	0	1	0	0	5	0.04065041	123
PDCD1LG2	9p24.2	DEL	3					3	0.02439024	123
JAK2	9p24	DEL	3		0	0	1	4	0.03252033	123
IRS1	2q36	DEL	3					3	0.02439024	123
CD274	9p24	DEL	3					3	0.02439024	123
CREBBP			0	2	0	0	0	2	0.01626016	123

Supplementary Table 2. Next generation sequencing detected InDels in the Trp53 locus targeted by CRISPR/Cas9.

SAMPLE	READS.ALIGNED	Unmodified Reads	Unmodified (%)	Mutant Reads	InDel (%)
S7	57699	52529	91.04	5170	8.96
S11	53615	47621	88.82	5994	11.18
S13	55304	48583	87.85	6721	12.15
S14	65588	29444	44.89	36144	55.11
S15	64401	27771	43.12	36630	56.88
S17	94934	30627	32.26	64307	67.74

Supplementary Table 3. The rate of metastasis detected in the mice bearing different gene mutation driven tumors.

Genotype	Tumors	Mets (Lung, otherwise specified)	Lung Met Rate	Note
Trp53/Rb1 CRISPR	11	1	9.09	
Trp53/Rb1 Cre	2	1	50.00	
Trp53/Bap1 CRISPR	16	4	25.00	
Trp53/Rb1/Bap1 Cre/	6	1	16.67	

Supplementary Table 4. Oligonucleotides.

Number	Target Gene ID	Target Gene Symbol	sgRNA Target Sequence	FWD oligoes	REV oligoes
73664	404545	Ano7	ACTGGCAGGAGTAATACTCG	**CACCG**ACTGGCAGGAGTAATACTCG	**AAAC**CGAGTATTACTCCTGCCAGT**C**
73665	404545	Ano7	CCATGTGCGAAGGTACTTCG	**CACCG**CCATGTGCGAAGGTACTTCG	**AAAC**CGAAGTACCTTCGCACATGG**C**
73666	404545	Ano7	GAAGGCGATATACACGGGCG	**CACCG**GAAGGCGATATACACGGGCG	**AAAC**CGCCCGTGTATATCGCCTTC**C**
73667	404545	Ano7	ATAGATGGCTTACCAATTGG	**CACCG**ATAGATGGCTTACCAATTGG	**AAAC**CCAATTGGTAAGCCATCTAT**C**
46056	94109	Csmd1	GTACTTACCAATGCACAAAG	**CACCG**GTACTTACCAATGCACAAAG	**AAAC**CTTTGTGCATTGGTAAGTAC**C**
46057	94109	Csmd1	GTCACACACTCAATGGACGA	**CACCG**GTCACACACTCAATGGACGA	**AAAC**TCGTCCATTGAGTGTGTGAC**C**
46058	94109	Csmd1	CGAACTGAAACCCAATTCTG	**CACCG**CGAACTGAAACCCAATTCTG	**AAAC**CAGAATTGGGTTTCAGTTCG**C**
46059	94109	Csmd1	GTGGTACTCTCCTATGAGTG	**CACCG**GTGGTACTCTCCTATGAGTG	**AAAC**CACTCATAGGAGAGTACCAC**C**
11314	17933	Myt1l	TCCCCCAAAGGATATGACGA	**CACCG**TCCCCCAAAGGATATGACGA	**AAAC**TCGTCATATCCTTTGGGGGA**C**
11315	17933	Myt1l	CTCATAACATTCGTCTACTG	**CACCG**CTCATAACATTCGTCTACTG	**AAAC**CAGTAGACGAATGTTATGAG**C**
11316	17933	Myt1l	GTGACAGAAGTTATGCTGAG	**CACCG**GTGACAGAAGTTATGCTGAG	**AAAC**CTCAGCATAACTTCTGTCAC**C**
11317	17933	Myt1l	GCGGGTAAAGCCCAGTTACG	**CACCG**GCGGGTAAAGCCCAGTTACG	**AAAC**CGTAACTGGGCTTTACCCGC**C**

Supplementary Table 5. Oligonucleotide pools for synthesis.

Number	Target Ge	Target Gene Symb	Left arm sequence	sgRNA Target Sequence	Right arm sequence	Oligonucleotide pools for synthesis
14482	19645	Rb1	ATATATCTTGTGGAAAGGACGAAACACCG	AAATGATACGAGGATTATCG	GTTTTAGAGCTAGAAATAGCAAGTTAAAA	ATATATCTTGTGGAAAGGACGAAACACCGAAATGATACGAGGATTATCGGTTTTAGAGCTAGAAATAGCAAGTTAAAA
14483	19645	Rb1	ATATATCTTGTGGAAAGGACGAAACACCG	AGAGAAGTTTGCTAACGCTG	GTTTTAGAGCTAGAAATAGCAAGTTAAAA	ATATATCTTGTGGAAAGGACGAAACACCGAGAGAAGTTTGCTAACGCTGGTTTTAGAGCTAGAAATAGCAAGTTAAAA
14484	19645	Rb1	ATATATCTTGTGGAAAGGACGAAACACCG	TAAGTACGTTCAGAATCCAC	GTTTTAGAGCTAGAAATAGCAAGTTAAAA	ATATATCTTGTGGAAAGGACGAAACACCGTAAGTACGTTCAGAATCCACGTTTTAGAGCTAGAAATAGCAAGTTAAAA
14485	19645	Rb1	ATATATCTTGTGGAAAGGACGAAACACCG	GCAGTATGGTTACCCTGGAG	GTTTTAGAGCTAGAAATAGCAAGTTAAAA	ATATATCTTGTGGAAAGGACGAAACACCGGCAGTATGGTTACCCTGGAGGTTTTAGAGCTAGAAATAGCAAGTTAAAA
35366	70086	Cysltr2	ATATATCTTGTGGAAAGGACGAAACACCG	AAACCCATATGATCCCACAG	GTTTTAGAGCTAGAAATAGCAAGTTAAAA	ATATATCTTGTGGAAAGGACGAAACACCGAAACCCATATGATCCCACAGGTTTTAGAGCTAGAAATAGCAAGTTAAAA
35367	70086	Cysltr2	ATATATCTTGTGGAAAGGACGAAACACCG	AACCACATTGCAGTAGCGGT	GTTTTAGAGCTAGAAATAGCAAGTTAAAA	ATATATCTTGTGGAAAGGACGAAACACCGAACCACATTGCAGTAGCGGTGTTTTAGAGCTAGAAATAGCAAGTTAAAA
35368	70086	Cysltr2	ATATATCTTGTGGAAAGGACGAAACACCG	TATGGAAAAGCCATTTCCCA	GTTTTAGAGCTAGAAATAGCAAGTTAAAA	ATATATCTTGTGGAAAGGACGAAACACCGTATGGAAAAGCCATTTCCCAGTTTTAGAGCTAGAAATAGCAAGTTAAAA
35369	70086	Cysltr2	ATATATCTTGTGGAAAGGACGAAACACCG	GATGAATAGAAAATCGGAAG	GTTTTAGAGCTAGAAATAGCAAGTTAAAA	ATATATCTTGTGGAAAGGACGAAACACCGGATGAATAGAAAATCGGAAGGTTTTAGAGCTAGAAATAGCAAGTTAAAA
1049	11848	Rhoa	ATATATCTTGTGGAAAGGACGAAACACCG	AAAACACATCAATATAACAT	GTTTTAGAGCTAGAAATAGCAAGTTAAAA	ATATATCTTGTGGAAAGGACGAAACACCGAAAACACATCAATATAACATGTTTTAGAGCTAGAAATAGCAAGTTAAAA
1050	11848	Rhoa	ATATATCTTGTGGAAAGGACGAAACACCG	CTATGTGGCGGATATCGAGG	GTTTTAGAGCTAGAAATAGCAAGTTAAAA	ATATATCTTGTGGAAAGGACGAAACACCGCTATGTGGCGGATATCGAGGGTTTTAGAGCTAGAAATAGCAAGTTAAAA
1051	11848	Rhoa	ATATATCTTGTGGAAAGGACGAAACACCG	GCTACTCACCTAAACTATCA	GTTTTAGAGCTAGAAATAGCAAGTTAAAA	ATATATCTTGTGGAAAGGACGAAACACCGGCTACTCACCTAAACTATCAGTTTTAGAGCTAGAAATAGCAAGTTAAAA
1052	11848	Rhoa	ATATATCTTGTGGAAAGGACGAAACACCG	CAGCAAGGACCAGTTCCCAG	GTTTTAGAGCTAGAAATAGCAAGTTAAAA	ATATATCTTGTGGAAAGGACGAAACACCGCAGCAAGGACCAGTTCCCAGGTTTTAGAGCTAGAAATAGCAAGTTAAAA
46424	98314	D2hgdh	ATATATCTTGTGGAAAGGACGAAACACCG	GGCTCACAGAGCATACTCCG	GTTTTAGAGCTAGAAATAGCAAGTTAAAA	ATATATCTTGTGGAAAGGACGAAACACCGGGCTCACAGAGCATACTCCGGTTTTAGAGCTAGAAATAGCAAGTTAAAA
46425	98314	D2hgdh	ATATATCTTGTGGAAAGGACGAAACACCG	AGGCCTGGGTGGGCACACAA	GTTTTAGAGCTAGAAATAGCAAGTTAAAA	ATATATCTTGTGGAAAGGACGAAACACCGAGGCCTGGGTGGGCACACAAGTTTTAGAGCTAGAAATAGCAAGTTAAAA
46426	98314	D2hgdh	ATATATCTTGTGGAAAGGACGAAACACCG	GGATACTGAATGCATGCAGT	GTTTTAGAGCTAGAAATAGCAAGTTAAAA	ATATATCTTGTGGAAAGGACGAAACACCGGGATACTGAATGCATGCAGTGTTTTAGAGCTAGAAATAGCAAGTTAAAA
46427	98314	D2hgdh	ATATATCTTGTGGAAAGGACGAAACACCG	GACTGGCTGAAGACCGTCCG	GTTTTAGAGCTAGAAATAGCAAGTTAAAA	ATATATCTTGTGGAAAGGACGAAACACCGGACTGGCTGAAGACCGTCCGGTTTTAGAGCTAGAAATAGCAAGTTAAAA
29438	66902	Mtap	ATATATCTTGTGGAAAGGACGAAACACCG	TGTGGATACTCCATTCGGCA	GTTTTAGAGCTAGAAATAGCAAGTTAAAA	ATATATCTTGTGGAAAGGACGAAACACCGTGTGGATACTCCATTCGGCAGTTTTAGAGCTAGAAATAGCAAGTTAAAA
29439	66902	Mtap	ATATATCTTGTGGAAAGGACGAAACACCG	GGACAATAGTCACAATTGAG	GTTTTAGAGCTAGAAATAGCAAGTTAAAA	ATATATCTTGTGGAAAGGACGAAACACCGGGACAATAGTCACAATTGAGGTTTTAGAGCTAGAAATAGCAAGTTAAAA
29440	66902	Mtap	ATATATCTTGTGGAAAGGACGAAACACCG	GGCAAAACGGTTCAGCCATG	GTTTTAGAGCTAGAAATAGCAAGTTAAAA	ATATATCTTGTGGAAAGGACGAAACACCGGGCAAAACGGTTCAGCCATGGTTTTAGAGCTAGAAATAGCAAGTTAAAA
29441	66902	Mtap	ATATATCTTGTGGAAAGGACGAAACACCG	GCCTTCAAAAGTCAACTACC	GTTTTAGAGCTAGAAATAGCAAGTTAAAA	ATATATCTTGTGGAAAGGACGAAACACCGGCCTTCAAAAGTCAACTACCGTTTTAGAGCTAGAAATAGCAAGTTAAAA
2669	12579	Cdkn2b	ATATATCTTGTGGAAAGGACGAAACACCG	GTGCACGACGCAGCTCGGGA	GTTTTAGAGCTAGAAATAGCAAGTTAAAA	ATATATCTTGTGGAAAGGACGAAACACCGGTGCACGACGCAGCTCGGGAGTTTTAGAGCTAGAAATAGCAAGTTAAAA
2670	12579	Cdkn2b	ATATATCTTGTGGAAAGGACGAAACACCG	GTCGTGCACAGGTCTGGTAA	GTTTTAGAGCTAGAAATAGCAAGTTAAAA	ATATATCTTGTGGAAAGGACGAAACACCGGTCGTGCACAGGTCTGGTAAGTTTTAGAGCTAGAAATAGCAAGTTAAAA
2671	12579	Cdkn2b	ATATATCTTGTGGAAAGGACGAAACACCG	CAGGGCGTTGGGATCTGCGC	GTTTTAGAGCTAGAAATAGCAAGTTAAAA	ATATATCTTGTGGAAAGGACGAAACACCGCAGGGCGTTGGGATCTGCGCGTTTTAGAGCTAGAAATAGCAAGTTAAAA
2672	12579	Cdkn2b	ATATATCTTGTGGAAAGGACGAAACACCG	GGATTGGGCGCCTCCCGAAG	GTTTTAGAGCTAGAAATAGCAAGTTAAAA	ATATATCTTGTGGAAAGGACGAAACACCGGGATTGGGCGCCTCCCGAAGGTTTTAGAGCTAGAAATAGCAAGTTAAAA
68212	269224	Pask	ATATATCTTGTGGAAAGGACGAAACACCG	GTACCGAACTATCATATGTG	GTTTTAGAGCTAGAAATAGCAAGTTAAAA	ATATATCTTGTGGAAAGGACGAAACACCGGTACCGAACTATCATATGTGGTTTTAGAGCTAGAAATAGCAAGTTAAAA
68213	269224	Pask	ATATATCTTGTGGAAAGGACGAAACACCG	CTGTCCTTAACTGAAACTAG	GTTTTAGAGCTAGAAATAGCAAGTTAAAA	ATATATCTTGTGGAAAGGACGAAACACCGCTGTCCTTAACTGAAACTAGGTTTTAGAGCTAGAAATAGCAAGTTAAAA
68214	269224	Pask	ATATATCTTGTGGAAAGGACGAAACACCG	TACCACATCTGAACTCAGCG	GTTTTAGAGCTAGAAATAGCAAGTTAAAA	ATATATCTTGTGGAAAGGACGAAACACCGTACCACATCTGAACTCAGCGGTTTTAGAGCTAGAAATAGCAAGTTAAAA
68215	269224	Pask	ATATATCTTGTGGAAAGGACGAAACACCG	TGCAGTGGTGTTCGGCACAG	GTTTTAGAGCTAGAAATAGCAAGTTAAAA	ATATATCTTGTGGAAAGGACGAAACACCGTGCAGTGGTGTTCGGCACAGGTTTTAGAGCTAGAAATAGCAAGTTAAAA
52568	208777	Sned1	ATATATCTTGTGGAAAGGACGAAACACCG	CATCGATTCTAAACGCCCAG	GTTTTAGAGCTAGAAATAGCAAGTTAAAA	ATATATCTTGTGGAAAGGACGAAACACCGCATCGATTCTAAACGCCCAGGTTTTAGAGCTAGAAATAGCAAGTTAAAA
52569	208777	Sned1	ATATATCTTGTGGAAAGGACGAAACACCG	GGTGGACTGTGGTCACCCTG	GTTTTAGAGCTAGAAATAGCAAGTTAAAA	ATATATCTTGTGGAAAGGACGAAACACCGGGTGGACTGTGGTCACCCTGGTTTTAGAGCTAGAAATAGCAAGTTAAAA
52570	208777	Sned1	ATATATCTTGTGGAAAGGACGAAACACCG	AGGTGATGTCTACTACCGGG	GTTTTAGAGCTAGAAATAGCAAGTTAAAA	ATATATCTTGTGGAAAGGACGAAACACCGAGGTGATGTCTACTACCGGGGTTTTAGAGCTAGAAATAGCAAGTTAAAA
52571	208777	Sned1	ATATATCTTGTGGAAAGGACGAAACACCG	CGCACGTGTAGGAGTCCTCG	GTTTTAGAGCTAGAAATAGCAAGTTAAAA	ATATATCTTGTGGAAAGGACGAAACACCGCGCACGTGTAGGAGTCCTCGGTTTTAGAGCTAGAAATAGCAAGTTAAAA
5389	14107	Fat1	ATATATCTTGTGGAAAGGACGAAACACCG	GTGCGCGCCCATCATAACCG	GTTTTAGAGCTAGAAATAGCAAGTTAAAA	ATATATCTTGTGGAAAGGACGAAACACCGGTGCGCGCCCATCATAACCGGTTTTAGAGCTAGAAATAGCAAGTTAAAA
5390	14107	Fat1	ATATATCTTGTGGAAAGGACGAAACACCG	ACGACCTCGGTATACCCCAG	GTTTTAGAGCTAGAAATAGCAAGTTAAAA	ATATATCTTGTGGAAAGGACGAAACACCGACGACCTCGGTATACCCCAGGTTTTAGAGCTAGAAATAGCAAGTTAAAA
5391	14107	Fat1	ATATATCTTGTGGAAAGGACGAAACACCG	ACGGTCAAACGGTATCCCGG	GTTTTAGAGCTAGAAATAGCAAGTTAAAA	ATATATCTTGTGGAAAGGACGAAACACCGACGGTCAAACGGTATCCCGGGTTTTAGAGCTAGAAATAGCAAGTTAAAA
5392	14107	Fat1	ATATATCTTGTGGAAAGGACGAAACACCG	TCCGTCGCTCGAATCACCAG	GTTTTAGAGCTAGAAATAGCAAGTTAAAA	ATATATCTTGTGGAAAGGACGAAACACCGTCCGTCGCTCGAATCACCAGGTTTTAGAGCTAGAAATAGCAAGTTAAAA
52552	208727	Hdac4	ATATATCTTGTGGAAAGGACGAAACACCG	TGTGGCTGCCAAATGACACA	GTTTTAGAGCTAGAAATAGCAAGTTAAAA	ATATATCTTGTGGAAAGGACGAAACACCGTGTGGCTGCCAAATGACACAGTTTTAGAGCTAGAAATAGCAAGTTAAAA
52553	208727	Hdac4	ATATATCTTGTGGAAAGGACGAAACACCG	CCTACCTAGATCGGCTACCT	GTTTTAGAGCTAGAAATAGCAAGTTAAAA	ATATATCTTGTGGAAAGGACGAAACACCGCCTACCTAGATCGGCTACCTGTTTTAGAGCTAGAAATAGCAAGTTAAAA
52554	208727	Hdac4	ATATATCTTGTGGAAAGGACGAAACACCG	CTTCTGTCTACAGACGAGCT	GTTTTAGAGCTAGAAATAGCAAGTTAAAA	ATATATCTTGTGGAAAGGACGAAACACCGCTTCTGTCTACAGACGAGCTGTTTTAGAGCTAGAAATAGCAAGTTAAAA
52555	208727	Hdac4	ATATATCTTGTGGAAAGGACGAAACACCG	TCACTTACCCATACCAGTAG	GTTTTAGAGCTAGAAATAGCAAGTTAAAA	ATATATCTTGTGGAAAGGACGAAACACCGTCACTTACCCATACCAGTAGGTTTTAGAGCTAGAAATAGCAAGTTAAAA
44764	80707	Wwox	ATATATCTTGTGGAAAGGACGAAACACCG	CTTCGTCGGATTATCGTCCA	GTTTTAGAGCTAGAAATAGCAAGTTAAAA	ATATATCTTGTGGAAAGGACGAAACACCGCTTCGTCGGATTATCGTCCAGTTTTAGAGCTAGAAATAGCAAGTTAAAA
44765	80707	Wwox	ATATATCTTGTGGAAAGGACGAAACACCG	CTTGGCAGTCTCGAACCCTG	GTTTTAGAGCTAGAAATAGCAAGTTAAAA	ATATATCTTGTGGAAAGGACGAAACACCGCTTGGCAGTCTCGAACCCTGGTTTTAGAGCTAGAAATAGCAAGTTAAAA
44766	80707	Wwox	ATATATCTTGTGGAAAGGACGAAACACCG	AAGAGGAAACGGGTCGCAGG	GTTTTAGAGCTAGAAATAGCAAGTTAAAA	ATATATCTTGTGGAAAGGACGAAACACCGAAGAGGAAACGGGTCGCAGGGTTTTAGAGCTAGAAATAGCAAGTTAAAA
44767	80707	Wwox	ATATATCTTGTGGAAAGGACGAAACACCG	ATTGCAGCACAAAGCCAAGG	GTTTTAGAGCTAGAAATAGCAAGTTAAAA	ATATATCTTGTGGAAAGGACGAAACACCGATTGCAGCACAAAGCCAAGGGTTTTAGAGCTAGAAATAGCAAGTTAAAA
18966	22589	Atrx	ATATATCTTGTGGAAAGGACGAAACACCG	GAATGGCCGTAAAAGTTCTG	GTTTTAGAGCTAGAAATAGCAAGTTAAAA	ATATATCTTGTGGAAAGGACGAAACACCGGAATGGCCGTAAAAGTTCTGGTTTTAGAGCTAGAAATAGCAAGTTAAAA
18967	22589	Atrx	ATATATCTTGTGGAAAGGACGAAACACCG	GACAATCTCAAAAAACGCGG	GTTTTAGAGCTAGAAATAGCAAGTTAAAA	ATATATCTTGTGGAAAGGACGAAACACCGGACAATCTCAAAAAACGCGGGTTTTAGAGCTAGAAATAGCAAGTTAAAA
18968	22589	Atrx	ATATATCTTGTGGAAAGGACGAAACACCG	GGGAGTTGCTCCATAGCAGG	GTTTTAGAGCTAGAAATAGCAAGTTAAAA	ATATATCTTGTGGAAAGGACGAAACACCGGGGAGTTGCTCCATAGCAGGGTTTTAGAGCTAGAAATAGCAAGTTAAAA
18969	22589	Atrx	ATATATCTTGTGGAAAGGACGAAACACCG	ACATACCTAACTGCTCTCTG	GTTTTAGAGCTAGAAATAGCAAGTTAAAA	ATATATCTTGTGGAAAGGACGAAACACCGACATACCTAACTGCTCTCTGGTTTTAGAGCTAGAAATAGCAAGTTAAAA
12666	18566	Pdcd1	ATATATCTTGTGGAAAGGACGAAACACCG	CAATACAGGGATACCCACTA	GTTTTAGAGCTAGAAATAGCAAGTTAAAA	ATATATCTTGTGGAAAGGACGAAACACCGCAATACAGGGATACCCACTAGTTTTAGAGCTAGAAATAGCAAGTTAAAA
12667	18566	Pdcd1	ATATATCTTGTGGAAAGGACGAAACACCG	GACACACGGCGCAATGACAG	GTTTTAGAGCTAGAAATAGCAAGTTAAAA	ATATATCTTGTGGAAAGGACGAAACACCGGACACACGGCGCAATGACAGGTTTTAGAGCTAGAAATAGCAAGTTAAAA
12668	18566	Pdcd1	ATATATCTTGTGGAAAGGACGAAACACCG	CAGCTTGTCCAACTGGTCGG	GTTTTAGAGCTAGAAATAGCAAGTTAAAA	ATATATCTTGTGGAAAGGACGAAACACCGCAGCTTGTCCAACTGGTCGGGTTTTAGAGCTAGAAATAGCAAGTTAAAA
12669	18566	Pdcd1	ATATATCTTGTGGAAAGGACGAAACACCG	GCTCAAACCATTACAGAAGG	GTTTTAGAGCTAGAAATAGCAAGTTAAAA	ATATATCTTGTGGAAAGGACGAAACACCGGCTCAAACCATTACAGAAGGGTTTTAGAGCTAGAAATAGCAAGTTAAAA
3065	12778	Ackr3	ATATATCTTGTGGAAAGGACGAAACACCG	ACACCAAGATGCATACAACA	GTTTTAGAGCTAGAAATAGCAAGTTAAAA	ATATATCTTGTGGAAAGGACGAAACACCGACACCAAGATGCATACAACAGTTTTAGAGCTAGAAATAGCAAGTTAAAA
3066	12778	Ackr3	ATATATCTTGTGGAAAGGACGAAACACCG	GCATAACCAGTGGCCCATGG	GTTTTAGAGCTAGAAATAGCAAGTTAAAA	ATATATCTTGTGGAAAGGACGAAACACCGGCATAACCAGTGGCCCATGGGTTTTAGAGCTAGAAATAGCAAGTTAAAA
3067	12778	Ackr3	ATATATCTTGTGGAAAGGACGAAACACCG	GGTCCACGCTCATGCAGGCG	GTTTTAGAGCTAGAAATAGCAAGTTAAAA	ATATATCTTGTGGAAAGGACGAAACACCGGGTCCACGCTCATGCAGGCGGTTTTAGAGCTAGAAATAGCAAGTTAAAA
3068	12778	Ackr3	ATATATCTTGTGGAAAGGACGAAACACCG	CCAACAATGAGACCTACTGC	GTTTTAGAGCTAGAAATAGCAAGTTAAAA	ATATATCTTGTGGAAAGGACGAAACACCGCCAACAATGAGACCTACTGCGTTTTAGAGCTAGAAATAGCAAGTTAAAA
	110083	DNAH12-1	ATATATCTTGTGGAAAGGACGAAACACCG	AAAAGAATCACGTCGCATGG	GTTTTAGAGCTAGAAATAGCAAGTTAAAA	ATATATCTTGTGGAAAGGACGAAACACCGAAAAGAATCACGTCGCATGGGTTTTAGAGCTAGAAATAGCAAGTTAAAA
	110083	DNAH12-1	ATATATCTTGTGGAAAGGACGAAACACCG	GAAATGATGATTGTTCGACA	GTTTTAGAGCTAGAAATAGCAAGTTAAAA	ATATATCTTGTGGAAAGGACGAAACACCGGAAATGATGATTGTTCGACAGTTTTAGAGCTAGAAATAGCAAGTTAAAA
	110083	DNAH12-2	ATATATCTTGTGGAAAGGACGAAACACCG	CTGTAACATCTGTCAGTTAA	GTTTTAGAGCTAGAAATAGCAAGTTAAAA	ATATATCTTGTGGAAAGGACGAAACACCGCTGTAACATCTGTCAGTTAAGTTTTAGAGCTAGAAATAGCAAGTTAAAA
	110083	DNAH12-2	ATATATCTTGTGGAAAGGACGAAACACCG	GGGCTAGACTATCTAGCAAT	GTTTTAGAGCTAGAAATAGCAAGTTAAAA	ATATATCTTGTGGAAAGGACGAAACACCGGGGCTAGACTATCTAGCAATGTTTTAGAGCTAGAAATAGCAAGTTAAAA
5609	14198	Fhit	ATATATCTTGTGGAAAGGACGAAACACCG	AACGGGTTTCCTATTCACCA	GTTTTAGAGCTAGAAATAGCAAGTTAAAA	ATATATCTTGTGGAAAGGACGAAACACCGAACGGGTTTCCTATTCACCAGTTTTAGAGCTAGAAATAGCAAGTTAAAA
5610	14198	Fhit	ATATATCTTGTGGAAAGGACGAAACACCG	CTTCATCAGGATGTAGGTCA	GTTTTAGAGCTAGAAATAGCAAGTTAAAA	ATATATCTTGTGGAAAGGACGAAACACCGCTTCATCAGGATGTAGGTCAGTTTTAGAGCTAGAAATAGCAAGTTAAAA
5611	14198	Fhit	ATATATCTTGTGGAAAGGACGAAACACCG	AAGCGCTCTACTGGCCTCAG	GTTTTAGAGCTAGAAATAGCAAGTTAAAA	ATATATCTTGTGGAAAGGACGAAACACCGAAGCGCTCTACTGGCCTCAGGTTTTAGAGCTAGAAATAGCAAGTTAAAA
5612	14198	Fhit	ATATATCTTGTGGAAAGGACGAAACACCG	CCAGAGAGTTGGGACAGTGG	GTTTTAGAGCTAGAAATAGCAAGTTAAAA	ATATATCTTGTGGAAAGGACGAAACACCGCCAGAGAGTTGGGACAGTGGGTTTTAGAGCTAGAAATAGCAAGTTAAAA
8685	16331	Inpp5d	ATATATCTTGTGGAAAGGACGAAACACCG	TGGGGATCTCAACTACCGCG	GTTTTAGAGCTAGAAATAGCAAGTTAAAA	ATATATCTTGTGGAAAGGACGAAACACCGTGGGGATCTCAACTACCGCGGTTTTAGAGCTAGAAATAGCAAGTTAAAA
8686	16331	Inpp5d	ATATATCTTGTGGAAAGGACGAAACACCG	CCACTATGCGAATGTTCCAG	GTTTTAGAGCTAGAAATAGCAAGTTAAAA	ATATATCTTGTGGAAAGGACGAAACACCGCCACTATGCGAATGTTCCAGGTTTTAGAGCTAGAAATAGCAAGTTAAAA
8687	16331	Inpp5d	ATATATCTTGTGGAAAGGACGAAACACCG	AGAGGCCAGTCCCATCACCA	GTTTTAGAGCTAGAAATAGCAAGTTAAAA	ATATATCTTGTGGAAAGGACGAAACACCGAGAGGCCAGTCCCATCACCAGTTTTAGAGCTAGAAATAGCAAGTTAAAA
8688	16331	Inpp5d	ATATATCTTGTGGAAAGGACGAAACACCG	CATCCGAAGGTGTCCCCATG	GTTTTAGAGCTAGAAATAGCAAGTTAAAA	ATATATCTTGTGGAAAGGACGAAACACCGCATCCGAAGGTGTCCCCATGGTTTTAGAGCTAGAAATAGCAAGTTAAAA
35406	70122	Mllt3	ATATATCTTGTGGAAAGGACGAAACACCG	AACTTTCAATAACCCCACGG	GTTTTAGAGCTAGAAATAGCAAGTTAAAA	ATATATCTTGTGGAAAGGACGAAACACCGAACTTTCAATAACCCCACGGGTTTTAGAGCTAGAAATAGCAAGTTAAAA
35407	70122	Mllt3	ATATATCTTGTGGAAAGGACGAAACACCG	AAGGAAAAAGAGTAGCTCGG	GTTTTAGAGCTAGAAATAGCAAGTTAAAA	ATATATCTTGTGGAAAGGACGAAACACCGAAGGAAAAAGAGTAGCTCGGGTTTTAGAGCTAGAAATAGCAAGTTAAAA
35408	70122	Mllt3	ATATATCTTGTGGAAAGGACGAAACACCG	TCCACGATGTCATCAAACGG	GTTTTAGAGCTAGAAATAGCAAGTTAAAA	ATATATCTTGTGGAAAGGACGAAACACCGTCCACGATGTCATCAAACGGGTTTTAGAGCTAGAAATAGCAAGTTAAAA
35409	70122	Mllt3	ATATATCTTGTGGAAAGGACGAAACACCG	ACTTACTCACCGTCACCAGT	GTTTTAGAGCTAGAAATAGCAAGTTAAAA	ATATATCTTGTGGAAAGGACGAAACACCGACTTACTCACCGTCACCAGTGTTTTAGAGCTAGAAATAGCAAGTTAAAA
15134	20190	Ryr1	ATATATCTTGTGGAAAGGACGAAACACCG	CTTCAAACTCGAAGTACCAG	GTTTTAGAGCTAGAAATAGCAAGTTAAAA	ATATATCTTGTGGAAAGGACGAAACACCGCTTCAAACTCGAAGTACCAGGTTTTAGAGCTAGAAATAGCAAGTTAAAA
15135	20190	Ryr1	ATATATCTTGTGGAAAGGACGAAACACCG	GGGACAACGTAACCACGCTG	GTTTTAGAGCTAGAAATAGCAAGTTAAAA	ATATATCTTGTGGAAAGGACGAAACACCGGGGACAACGTAACCACGCTGGTTTTAGAGCTAGAAATAGCAAGTTAAAA
15136	20190	Ryr1	ATATATCTTGTGGAAAGGACGAAACACCG	GCGATTGCCACGGATCAGAG	GTTTTAGAGCTAGAAATAGCAAGTTAAAA	ATATATCTTGTGGAAAGGACGAAACACCGGCGATTGCCACGGATCAGAGGTTTTAGAGCTAGAAATAGCAAGTTAAAA
15137	20190	Ryr1	ATATATCTTGTGGAAAGGACGAAACACCG	CATCAAAGAGTATCGCAGGG	GTTTTAGAGCTAGAAATAGCAAGTTAAAA	ATATATCTTGTGGAAAGGACGAAACACCGCATCAAAGAGTATCGCAGGGGTTTTAGAGCTAGAAATAGCAAGTTAAAA
60580	235587	Parp3	ATATATCTTGTGGAAAGGACGAAACACCG	TGATAATCGGGTCATCCGTG	GTTTTAGAGCTAGAAATAGCAAGTTAAAA	ATATATCTTGTGGAAAGGACGAAACACCGTGATAATCGGGTCATCCGTGGTTTTAGAGCTAGAAATAGCAAGTTAAAA
60581	235587	Parp3	ATATATCTTGTGGAAAGGACGAAACACCG	GCTGGTAGTCTCGATCCAGT	GTTTTAGAGCTAGAAATAGCAAGTTAAAA	ATATATCTTGTGGAAAGGACGAAACACCGGCTGGTAGTCTCGATCCAGTGTTTTAGAGCTAGAAATAGCAAGTTAAAA
60582	235587	Parp3	ATATATCTTGTGGAAAGGACGAAACACCG	GAAGACTAAAAACAAATGGG	GTTTTAGAGCTAGAAATAGCAAGTTAAAA	ATATATCTTGTGGAAAGGACGAAACACCGGAAGACTAAAAACAAATGGGGTTTTAGAGCTAGAAATAGCAAGTTAAAA
60583	235587	Parp3	ATATATCTTGTGGAAAGGACGAAACACCG	ACCCTGAACCAGACCAACAT	GTTTTAGAGCTAGAAATAGCAAGTTAAAA	ATATATCTTGTGGAAAGGACGAAACACCGACCCTGAACCAGACCAACATGTTTTAGAGCTAGAAATAGCAAGTTAAAA
14830	19882	Mst1r	ATATATCTTGTGGAAAGGACGAAACACCG	AAGTATCAGACTTTAGACGA	GTTTTAGAGCTAGAAATAGCAAGTTAAAA	ATATATCTTGTGGAAAGGACGAAACACCGAAGTATCAGACTTTAGACGAGTTTTAGAGCTAGAAATAGCAAGTTAAAA
14831	19882	Mst1r	ATATATCTTGTGGAAAGGACGAAACACCG	GGGAACACACCAGATCACCG	GTTTTAGAGCTAGAAATAGCAAGTTAAAA	ATATATCTTGTGGAAAGGACGAAACACCGGGGAACACACCAGATCACCGGTTTTAGAGCTAGAAATAGCAAGTTAAAA
14832	19882	Mst1r	ATATATCTTGTGGAAAGGACGAAACACCG	ATGTGGTCCGAAACCCTCAG	GTTTTAGAGCTAGAAATAGCAAGTTAAAA	ATATATCTTGTGGAAAGGACGAAACACCGATGTGGTCCGAAACCCTCAGGTTTTAGAGCTAGAAATAGCAAGTTAAAA
14833	19882	Mst1r	ATATATCTTGTGGAAAGGACGAAACACCG	AAGACGTGTCACATGCAGTG	GTTTTAGAGCTAGAAATAGCAAGTTAAAA	ATATATCTTGTGGAAAGGACGAAACACCGAAGACGTGTCACATGCAGTGGTTTTAGAGCTAGAAATAGCAAGTTAAAA
7561	15235	Mst1	ATATATCTTGTGGAAAGGACGAAACACCG	CGGTGAGGATTACCGTGGCG	GTTTTAGAGCTAGAAATAGCAAGTTAAAA	ATATATCTTGTGGAAAGGACGAAACACCGCGGTGAGGATTACCGTGGCGGTTTTAGAGCTAGAAATAGCAAGTTAAAA
7562	15235	Mst1	ATATATCTTGTGGAAAGGACGAAACACCG	GGCTCAGGTGAACAGTATCG	GTTTTAGAGCTAGAAATAGCAAGTTAAAA	ATATATCTTGTGGAAAGGACGAAACACCGGGCTCAGGTGAACAGTATCGGTTTTAGAGCTAGAAATAGCAAGTTAAAA
7563	15235	Mst1	ATATATCTTGTGGAAAGGACGAAACACCG	AAGTCACAGCGGCGCAACAA	GTTTTAGAGCTAGAAATAGCAAGTTAAAA	ATATATCTTGTGGAAAGGACGAAACACCGAAGTCACAGCGGCGCAACAAGTTTTAGAGCTAGAAATAGCAAGTTAAAA
7564	15235	Mst1	ATATATCTTGTGGAAAGGACGAAACACCG	GTAATCCGGACGGATCTGAG	GTTTTAGAGCTAGAAATAGCAAGTTAAAA	ATATATCTTGTGGAAAGGACGAAACACCGGTAATCCGGACGGATCTGAGGTTTTAGAGCTAGAAATAGCAAGTTAAAA
43128	76804	Kdm4c	ATATATCTTGTGGAAAGGACGAAACACCG	GTGCACTTGTCGGAATGACA	GTTTTAGAGCTAGAAATAGCAAGTTAAAA	ATATATCTTGTGGAAAGGACGAAACACCGGTGCACTTGTCGGAATGACAGTTTTAGAGCTAGAAATAGCAAGTTAAAA
43129	76804	Kdm4c	ATATATCTTGTGGAAAGGACGAAACACCG	CTGGCCGGAGGCTTACCAAG	GTTTTAGAGCTAGAAATAGCAAGTTAAAA	ATATATCTTGTGGAAAGGACGAAACACCGCTGGCCGGAGGCTTACCAAGGTTTTAGAGCTAGAAATAGCAAGTTAAAA
43130	76804	Kdm4c	ATATATCTTGTGGAAAGGACGAAACACCG	TTTGGATACCAGGATACAAG	GTTTTAGAGCTAGAAATAGCAAGTTAAAA	ATATATCTTGTGGAAAGGACGAAACACCGTTTGGATACCAGGATACAAGGTTTTAGAGCTAGAAATAGCAAGTTAAAA
43131	76804	Kdm4c	ATATATCTTGTGGAAAGGACGAAACACCG	TGTGGAAAACAACATTTGCG	GTTTTAGAGCTAGAAATAGCAAGTTAAAA	ATATATCTTGTGGAAAGGACGAAACACCGTGTGGAAAACAACATTTGCGGTTTTAGAGCTAGAAATAGCAAGTTAAAA
47196	101739	Psip1	ATATATCTTGTGGAAAGGACGAAACACCG	AGAGAGGACGACCTGCAGGT	GTTTTAGAGCTAGAAATAGCAAGTTAAAA	ATATATCTTGTGGAAAGGACGAAACACCGAGAGAGGACGACCTGCAGGTGTTTTAGAGCTAGAAATAGCAAGTTAAAA
47197	101739	Psip1	ATATATCTTGTGGAAAGGACGAAACACCG	GAAAAATTTAGCTAAACCAG	GTTTTAGAGCTAGAAATAGCAAGTTAAAA	ATATATCTTGTGGAAAGGACGAAACACCGGAAAAATTTAGCTAAACCAGGTTTTAGAGCTAGAAATAGCAAGTTAAAA
47198	101739	Psip1	ATATATCTTGTGGAAAGGACGAAACACCG	ACTCCAAAAGCTGCCAGGCG	GTTTTAGAGCTAGAAATAGCAAGTTAAAA	ATATATCTTGTGGAAAGGACGAAACACCGACTCCAAAAGCTGCCAGGCGGTTTTAGAGCTAGAAATAGCAAGTTAAAA
47199	101739	Psip1	ATATATCTTGTGGAAAGGACGAAACACCG	AAAAGAGACTAACGTTTCAA	GTTTTAGAGCTAGAAATAGCAAGTTAAAA	ATATATCTTGTGGAAAGGACGAAACACCGAAAAGAGACTAACGTTTCAAGTTTTAGAGCTAGAAATAGCAAGTTAAAA
29486	66923	Pbrm1	ATATATCTTGTGGAAAGGACGAAACACCG	AAAACACTTGCATAACGATG	GTTTTAGAGCTAGAAATAGCAAGTTAAAA	ATATATCTTGTGGAAAGGACGAAACACCGAAAACACTTGCATAACGATGGTTTTAGAGCTAGAAATAGCAAGTTAAAA
29487	66923	Pbrm1	ATATATCTTGTGGAAAGGACGAAACACCG	CAATGCCAGGCACTACAATG	GTTTTAGAGCTAGAAATAGCAAGTTAAAA	ATATATCTTGTGGAAAGGACGAAACACCGCAATGCCAGGCACTACAATGGTTTTAGAGCTAGAAATAGCAAGTTAAAA
29488	66923	Pbrm1	ATATATCTTGTGGAAAGGACGAAACACCG	AATAAAAGAGCAGTCCAAGG	GTTTTAGAGCTAGAAATAGCAAGTTAAAA	ATATATCTTGTGGAAAGGACGAAACACCGAATAAAAGAGCAGTCCAAGGGTTTTAGAGCTAGAAATAGCAAGTTAAAA
29489	66923	Pbrm1	ATATATCTTGTGGAAAGGACGAAACACCG	ACAAGCAGCTTTATACTCAG	GTTTTAGAGCTAGAAATAGCAAGTTAAAA	ATATATCTTGTGGAAAGGACGAAACACCGACAAGCAGCTTTATACTCAGGTTTTAGAGCTAGAAATAGCAAGTTAAAA
47816	104416	Bap1	ATATATCTTGTGGAAAGGACGAAACACCG	CCACCAACGTAGAAACCTTG	GTTTTAGAGCTAGAAATAGCAAGTTAAAA	ATATATCTTGTGGAAAGGACGAAACACCGCCACCAACGTAGAAACCTTGGTTTTAGAGCTAGAAATAGCAAGTTAAAA
47817	104416	Bap1	ATATATCTTGTGGAAAGGACGAAACACCG	GCAGGATTAAGTATGAGACC	GTTTTAGAGCTAGAAATAGCAAGTTAAAA	ATATATCTTGTGGAAAGGACGAAACACCGGCAGGATTAAGTATGAGACCGTTTTAGAGCTAGAAATAGCAAGTTAAAA
47818	104416	Bap1	ATATATCTTGTGGAAAGGACGAAACACCG	ACCTGTCTGAGTGCACTCAG	GTTTTAGAGCTAGAAATAGCAAGTTAAAA	ATATATCTTGTGGAAAGGACGAAACACCGACCTGTCTGAGTGCACTCAGGTTTTAGAGCTAGAAATAGCAAGTTAAAA
47819	104416	Bap1	ATATATCTTGTGGAAAGGACGAAACACCG	TCAGCTATGTGCCTATCACA	GTTTTAGAGCTAGAAATAGCAAGTTAAAA	ATATATCTTGTGGAAAGGACGAAACACCGTCAGCTATGTGCCTATCACAGTTTTAGAGCTAGAAATAGCAAGTTAAAA
26086	58205	Pdcd1lg2	ATATATCTTGTGGAAAGGACGAAACACCG	CCCCGCAGATGACCAGGCAA	GTTTTAGAGCTAGAAATAGCAAGTTAAAA	ATATATCTTGTGGAAAGGACGAAACACCGCCCCGCAGATGACCAGGCAAGTTTTAGAGCTAGAAATAGCAAGTTAAAA
26087	58205	Pdcd1lg2	ATATATCTTGTGGAAAGGACGAAACACCG	GATAGACACTAGGATCCTGG	GTTTTAGAGCTAGAAATAGCAAGTTAAAA	ATATATCTTGTGGAAAGGACGAAACACCGGATAGACACTAGGATCCTGGGTTTTAGAGCTAGAAATAGCAAGTTAAAA
26088	58205	Pdcd1lg2	ATATATCTTGTGGAAAGGACGAAACACCG	CCAGTTCAGTGCATTCTCTG	GTTTTAGAGCTAGAAATAGCAAGTTAAAA	ATATATCTTGTGGAAAGGACGAAACACCGCCAGTTCAGTGCATTCTCTGGTTTTAGAGCTAGAAATAGCAAGTTAAAA
26089	58205	Pdcd1lg2	ATATATCTTGTGGAAAGGACGAAACACCG	CTAGGGGATAACCTCTAGCC	GTTTTAGAGCTAGAAATAGCAAGTTAAAA	ATATATCTTGTGGAAAGGACGAAACACCGCTAGGGGATAACCTCTAGCCGTTTTAGAGCTAGAAATAGCAAGTTAAAA
8933	16452	Jak2	ATATATCTTGTGGAAAGGACGAAACACCG	CGGGTATTACAGACTAACTG	GTTTTAGAGCTAGAAATAGCAAGTTAAAA	ATATATCTTGTGGAAAGGACGAAACACCGCGGGTATTACAGACTAACTGGTTTTAGAGCTAGAAATAGCAAGTTAAAA
8934	16452	Jak2	ATATATCTTGTGGAAAGGACGAAACACCG	AAGTCCTAGATAAAGCACAT	GTTTTAGAGCTAGAAATAGCAAGTTAAAA	ATATATCTTGTGGAAAGGACGAAACACCGAAGTCCTAGATAAAGCACATGTTTTAGAGCTAGAAATAGCAAGTTAAAA
8935	16452	Jak2	ATATATCTTGTGGAAAGGACGAAACACCG	ACAGATACGGAGTGTCCCGT	GTTTTAGAGCTAGAAATAGCAAGTTAAAA	ATATATCTTGTGGAAAGGACGAAACACCGACAGATACGGAGTGTCCCGTGTTTTAGAGCTAGAAATAGCAAGTTAAAA
8936	16452	Jak2	ATATATCTTGTGGAAAGGACGAAACACCG	TCTTCAGGAGAGAATACCAT	GTTTTAGAGCTAGAAATAGCAAGTTAAAA	ATATATCTTGTGGAAAGGACGAAACACCGTCTTCAGGAGAGAATACCATGTTTTAGAGCTAGAAATAGCAAGTTAAAA
8737	16367	Irs1	ATATATCTTGTGGAAAGGACGAAACACCG	ACCCACGCCCATCGGCATCG	GTTTTAGAGCTAGAAATAGCAAGTTAAAA	ATATATCTTGTGGAAAGGACGAAACACCGACCCACGCCCATCGGCATCGGTTTTAGAGCTAGAAATAGCAAGTTAAAA
8738	16367	Irs1	ATATATCTTGTGGAAAGGACGAAACACCG	ATGCACCACTTGGAACGTCG	GTTTTAGAGCTAGAAATAGCAAGTTAAAA	ATATATCTTGTGGAAAGGACGAAACACCGATGCACCACTTGGAACGTCGGTTTTAGAGCTAGAAATAGCAAGTTAAAA
8739	16367	Irs1	ATATATCTTGTGGAAAGGACGAAACACCG	GGGAATCTGGGGTGACACTG	GTTTTAGAGCTAGAAATAGCAAGTTAAAA	ATATATCTTGTGGAAAGGACGAAACACCGGGGAATCTGGGGTGACACTGGTTTTAGAGCTAGAAATAGCAAGTTAAAA
8740	16367	Irs1	ATATATCTTGTGGAAAGGACGAAACACCG	GGAGGACTTGAGCTATGACA	GTTTTAGAGCTAGAAATAGCAAGTTAAAA	ATATATCTTGTGGAAAGGACGAAACACCGGGAGGACTTGAGCTATGACAGTTTTAGAGCTAGAAATAGCAAGTTAAAA
26694	60533	Cd274	ATATATCTTGTGGAAAGGACGAAACACCG	TGCTGCATAATCAGCTACGG	GTTTTAGAGCTAGAAATAGCAAGTTAAAA	ATATATCTTGTGGAAAGGACGAAACACCGTGCTGCATAATCAGCTACGGGTTTTAGAGCTAGAAATAGCAAGTTAAAA
26695	60533	Cd274	ATATATCTTGTGGAAAGGACGAAACACCG	TCCAAAGGACTTGTACGTGG	GTTTTAGAGCTAGAAATAGCAAGTTAAAA	ATATATCTTGTGGAAAGGACGAAACACCGTCCAAAGGACTTGTACGTGGGTTTTAGAGCTAGAAATAGCAAGTTAAAA
26696	60533	Cd274	ATATATCTTGTGGAAAGGACGAAACACCG	CTCAGCACAGCAACTTCAGG	GTTTTAGAGCTAGAAATAGCAAGTTAAAA	ATATATCTTGTGGAAAGGACGAAACACCGCTCAGCACAGCAACTTCAGGGTTTTAGAGCTAGAAATAGCAAGTTAAAA
26697	60533	Cd274	ATATATCTTGTGGAAAGGACGAAACACCG	GCTTGCGTTAGTGGTGTACT	GTTTTAGAGCTAGAAATAGCAAGTTAAAA	ATATATCTTGTGGAAAGGACGAAACACCGGCTTGCGTTAGTGGTGTACTGTTTTAGAGCTAGAAATAGCAAGTTAAAA
12026	18223	Numbl	ATATATCTTGTGGAAAGGACGAAACACCG	CTATCCTATGTCACTAGACG	GTTTTAGAGCTAGAAATAGCAAGTTAAAA	ATATATCTTGTGGAAAGGACGAAACACCGCTATCCTATGTCACTAGACGGTTTTAGAGCTAGAAATAGCAAGTTAAAA
12027	18223	Numbl	ATATATCTTGTGGAAAGGACGAAACACCG	ATGGCAGCTCATTCAACCGT	GTTTTAGAGCTAGAAATAGCAAGTTAAAA	ATATATCTTGTGGAAAGGACGAAACACCGATGGCAGCTCATTCAACCGTGTTTTAGAGCTAGAAATAGCAAGTTAAAA
12028	18223	Numbl	ATATATCTTGTGGAAAGGACGAAACACCG	TTCTCCTACATATGCCGCGA	GTTTTAGAGCTAGAAATAGCAAGTTAAAA	ATATATCTTGTGGAAAGGACGAAACACCGTTCTCCTACATATGCCGCGAGTTTTAGAGCTAGAAATAGCAAGTTAAAA
12029	18223	Numbl	ATATATCTTGTGGAAAGGACGAAACACCG	CCCGTGCACTCACCCTAACA	GTTTTAGAGCTAGAAATAGCAAGTTAAAA	ATATATCTTGTGGAAAGGACGAAACACCGCCCGTGCACTCACCCTAACAGTTTTAGAGCTAGAAATAGCAAGTTAAAA
3409	12914	Crebbp	ATATATCTTGTGGAAAGGACGAAACACCG	CATACTCGAGATATCCGATG	GTTTTAGAGCTAGAAATAGCAAGTTAAAA	ATATATCTTGTGGAAAGGACGAAACACCGCATACTCGAGATATCCGATGGTTTTAGAGCTAGAAATAGCAAGTTAAAA
3410	12914	Crebbp	ATATATCTTGTGGAAAGGACGAAACACCG	AGCTCTGAAAGATCGCCGCA	GTTTTAGAGCTAGAAATAGCAAGTTAAAA	ATATATCTTGTGGAAAGGACGAAACACCGAGCTCTGAAAGATCGCCGCAGTTTTAGAGCTAGAAATAGCAAGTTAAAA
3411	12914	Crebbp	ATATATCTTGTGGAAAGGACGAAACACCG	TAATGAATCAGGCTCAACAA	GTTTTAGAGCTAGAAATAGCAAGTTAAAA	ATATATCTTGTGGAAAGGACGAAACACCGTAATGAATCAGGCTCAACAAGTTTTAGAGCTAGAAATAGCAAGTTAAAA
3412	12914	Crebbp	ATATATCTTGTGGAAAGGACGAAACACCG	TGAACCTACTGAATCCAAGG	GTTTTAGAGCTAGAAATAGCAAGTTAAAA	ATATATCTTGTGGAAAGGACGAAACACCGTGAACCTACTGAATCCAAGGGTTTTAGAGCTAGAAATAGCAAGTTAAAA
14934	19989	Rpl7	ATATATCTTGTGGAAAGGACGAAACACCG	GAAGGCAAAGCACTATCACA	GTTTTAGAGCTAGAAATAGCAAGTTAAAA	ATATATCTTGTGGAAAGGACGAAACACCGGAAGGCAAAGCACTATCACAGTTTTAGAGCTAGAAATAGCAAGTTAAAA
14935	19989	Rpl7	ATATATCTTGTGGAAAGGACGAAACACCG	CCTTTGTCATCAGAATTCGA	GTTTTAGAGCTAGAAATAGCAAGTTAAAA	ATATATCTTGTGGAAAGGACGAAACACCGCCTTTGTCATCAGAATTCGAGTTTTAGAGCTAGAAATAGCAAGTTAAAA
14936	19989	Rpl7	ATATATCTTGTGGAAAGGACGAAACACCG	TGGAGCCATACATTGCATGG	GTTTTAGAGCTAGAAATAGCAAGTTAAAA	ATATATCTTGTGGAAAGGACGAAACACCGTGGAGCCATACATTGCATGGGTTTTAGAGCTAGAAATAGCAAGTTAAAA
14937	19989	Rpl7	ATATATCTTGTGGAAAGGACGAAACACCG	TTAAGAAAAAGGTTCCTGCT	GTTTTAGAGCTAGAAATAGCAAGTTAAAA	ATATATCTTGTGGAAAGGACGAAACACCGTTAAGAAAAAGGTTCCTGCTGTTTTAGAGCTAGAAATAGCAAGTTAAAA
15006	20085	Rps19	ATATATCTTGTGGAAAGGACGAAACACCG	GCACGGCACCTGTACCTCCG	GTTTTAGAGCTAGAAATAGCAAGTTAAAA	ATATATCTTGTGGAAAGGACGAAACACCGGCACGGCACCTGTACCTCCGGTTTTAGAGCTAGAAATAGCAAGTTAAAA
15007	20085	Rps19	ATATATCTTGTGGAAAGGACGAAACACCG	AACTGGTTCTACACACGAGC	GTTTTAGAGCTAGAAATAGCAAGTTAAAA	ATATATCTTGTGGAAAGGACGAAACACCGAACTGGTTCTACACACGAGCGTTTTAGAGCTAGAAATAGCAAGTTAAAA
15008	20085	Rps19	ATATATCTTGTGGAAAGGACGAAACACCG	TCATGACCAAGATCTATGG	GTTTTAGAGCTAGAAATAGCAAGTTAAAA	ATATATCTTGTGGAAAGGACGAAACACCGTCATGACCAAGATCTATGGGTTTTAGAGCTAGAAATAGCAAGTTAAAA
15009	20085	Rps19	ATATATCTTGTGGAAAGGACGAAACACCG	AGACCCAGCCATTCAGCAG	GTTTTAGAGCTAGAAATAGCAAGTTAAAA	ATATATCTTGTGGAAAGGACGAAACACCGAGACCCAGCCATTCAGCAGGTTTTAGAGCTAGAAATAGCAAGTTAAAA
14890	19934	Rpl22	ATATATCTTGTGGAAAGGACGAAACACCG	AAAAAGCTTGTGGCGAAGGG	GTTTTAGAGCTAGAAATAGCAAGTTAAAA	ATATATCTTGTGGAAAGGACGAAACACCGAAAAAGCTTGTGGCGAAGGGGTTTTAGAGCTAGAAATAGCAAGTTAAAA
14891	19934	Rpl22	ATATATCTTGTGGAAAGGACGAAACACCG	CAAGATCACTGTCACTTCAG	GTTTTAGAGCTAGAAATAGCAAGTTAAAA	ATATATCTTGTGGAAAGGACGAAACACCGCAAGATCACTGTCACTTCAGGTTTTAGAGCTAGAAATAGCAAGTTAAAA
14892	19934	Rpl22	ATATATCTTGTGGAAAGGACGAAACACCG	TGCTACGAACCTTTCTGCTC	GTTTTAGAGCTAGAAATAGCAAGTTAAAA	ATATATCTTGTGGAAAGGACGAAACACCGTGCTACGAACCTTTCTGCTCGTTTTAGAGCTAGAAATAGCAAGTTAAAA
14893	19934	Rpl22	ATATATCTTGTGGAAAGGACGAAACACCG	CAGTTAAGTTCTCTAAGAAG	GTTTTAGAGCTAGAAATAGCAAGTTAAAA	ATATATCTTGTGGAAAGGACGAAACACCGCAGTTAAGTTCTCTAAGAAGGTTTTAGAGCTAGAAATAGCAAGTTAAAA
15002	20084	Rps18	ATATATCTTGTGGAAAGGACGAAACACCG	TTTGACCTCAGGGCGTGGGG	GTTTTAGAGCTAGAAATAGCAAGTTAAAA	ATATATCTTGTGGAAAGGACGAAACACCGTTTGACCTCAGGGCGTGGGGGTTTTAGAGCTAGAAATAGCAAGTTAAAA
15003	20084	Rps18	ATATATCTTGTGGAAAGGACGAAACACCG	AGACATCGACCTCACCAAGA	GTTTTAGAGCTAGAAATAGCAAGTTAAAA	ATATATCTTGTGGAAAGGACGAAACACCGAGACATCGACCTCACCAAGAGTTTTAGAGCTAGAAATAGCAAGTTAAAA
15004	20084	Rps18	ATATATCTTGTGGAAAGGACGAAACACCG	GACAGTACAAGATCCCAGAC	GTTTTAGAGCTAGAAATAGCAAGTTAAAA	ATATATCTTGTGGAAAGGACGAAACACCGGACAGTACAAGATCCCAGACGTTTTAGAGCTAGAAATAGCAAGTTAAAA
15005	20084	Rps18	ATATATCTTGTGGAAAGGACGAAACACCG	AAAATCTAGGTTCTGGCCAA	GTTTTAGAGCTAGAAATAGCAAGTTAAAA	ATATATCTTGTGGAAAGGACGAAACACCGAAAATCTAGGTTCTGGCCAAGTTTTAGAGCTAGAAATAGCAAGTTAAAA
20826	27207	Rps11	ATATATCTTGTGGAAAGGACGAAACACCG	CGATATTCTTGTAGTACCGA	GTTTTAGAGCTAGAAATAGCAAGTTAAAA	ATATATCTTGTGGAAAGGACGAAACACCGCGATATTCTTGTAGTACCGAGTTTTAGAGCTAGAAATAGCAAGTTAAAA
20827	27207	Rps11	ATATATCTTGTGGAAAGGACGAAACACCG	AGGCTTCAAGACGCCTAAAG	GTTTTAGAGCTAGAAATAGCAAGTTAAAA	ATATATCTTGTGGAAAGGACGAAACACCGAGGCTTCAAGACGCCTAAAGGTTTTAGAGCTAGAAATAGCAAGTTAAAA
20828	27207	Rps11	ATATATCTTGTGGAAAGGACGAAACACCG	ATAGTCCCGGCGGATGACAA	GTTTTAGAGCTAGAAATAGCAAGTTAAAA	ATATATCTTGTGGAAAGGACGAAACACCGATAGTCCCGGCGGATGACAAGTTTTAGAGCTAGAAATAGCAAGTTAAAA
20829	27207	Rps11	ATATATCTTGTGGAAAGGACGAAACACCG	CAACATCCCACTCACCAGAC	GTTTTAGAGCTAGAAATAGCAAGTTAAAA	ATATATCTTGTGGAAAGGACGAAACACCGCAACATCCCACTCACCAGACGTTTTAGAGCTAGAAATAGCAAGTTAAAA
83078	NonTarge	MGLibA_66406	ATATATCTTGTGGAAAGGACGAAACACCG	GCGAGGTATTCGGCTCCGCG	GTTTTAGAGCTAGAAATAGCAAGTTAAAA	ATATATCTTGTGGAAAGGACGAAACACCGGCGAGGTATTCGGCTCCGCGGTTTTAGAGCTAGAAATAGCAAGTTAAAA
83079	NonTarge	MGLibA_66407	ATATATCTTGTGGAAAGGACGAAACACCG	GCTTTCACGGAGGTTCGACG	GTTTTAGAGCTAGAAATAGCAAGTTAAAA	ATATATCTTGTGGAAAGGACGAAACACCGGCTTTCACGGAGGTTCGACGGTTTTAGAGCTAGAAATAGCAAGTTAAAA
83080	NonTarge	MGLibA_66408	ATATATCTTGTGGAAAGGACGAAACACCG	ATGTTGCAGTTCGGCTCGAT	GTTTTAGAGCTAGAAATAGCAAGTTAAAA	ATATATCTTGTGGAAAGGACGAAACACCGATGTTGCAGTTCGGCTCGATGTTTTAGAGCTAGAAATAGCAAGTTAAAA
83081	NonTarge	MGLibA_66409	ATATATCTTGTGGAAAGGACGAAACACCG	ACGTGTAAGGCGAACGCCTT	GTTTTAGAGCTAGAAATAGCAAGTTAAAA	ATATATCTTGTGGAAAGGACGAAACACCGACGTGTAAGGCGAACGCCTTGTTTTAGAGCTAGAAATAGCAAGTTAAAA
83082	NonTarge	MGLibA_66410	ATATATCTTGTGGAAAGGACGAAACACCG	GACTCCGGGTACTAAATGTC	GTTTTAGAGCTAGAAATAGCAAGTTAAAA	ATATATCTTGTGGAAAGGACGAAACACCGGACTCCGGGTACTAAATGTCGTTTTAGAGCTAGAAATAGCAAGTTAAAA
83083	NonTarge	MGLibA_66411	ATATATCTTGTGGAAAGGACGAAACACCG	CCGCGCCGTTAGGGAACGAG	GTTTTAGAGCTAGAAATAGCAAGTTAAAA	ATATATCTTGTGGAAAGGACGAAACACCGCCGCGCCGTTAGGGAACGAGGTTTTAGAGCTAGAAATAGCAAGTTAAAA
83084	NonTarge	MGLibA_66412	ATATATCTTGTGGAAAGGACGAAACACCG	ATTGTTCGACCGTCTACGGG	GTTTTAGAGCTAGAAATAGCAAGTTAAAA	ATATATCTTGTGGAAAGGACGAAACACCGATTGTTCGACCGTCTACGGGGTTTTAGAGCTAGAAATAGCAAGTTAAAA
83085	NonTarge	MGLibA_66413	ATATATCTTGTGGAAAGGACGAAACACCG	ACCCATCGGGTGCGATATGG	GTTTTAGAGCTAGAAATAGCAAGTTAAAA	ATATATCTTGTGGAAAGGACGAAACACCGACCCATCGGGTGCGATATGGGTTTTAGAGCTAGAAATAGCAAGTTAAAA

Supplementary Table 6. Combined T-cell/MDSC panel.

Location	Antibody	Clone	Source
	Live/dead		ebio (65-0865-18)
	CD45	30-F11	BioLegend
	CD4	RM4-5	BioLegend
	CD8	53-6.7	BioLegend
	NKp46	29A1.4	BioLegend
	PD-1	J43	ThermoFisher
	Tim-3	215008	R&D Systems
	Lag-3	C9B7W	BioLegend
	CD122	TM-B1	BD Biosciences
Surface	CD62L	MEL-14	BioLegend
	MHCII	2G9	BD Biosciences
	KLRG1	2F1/KLRG1	BioLegend
	F4/80	T45-2342	BD Biosciences
	CD11c	N418	ThermoFisher
	CD11b	M1/70	ThermoFisher
	CD274 (PD-L1)	10F.9G2	BD Biosciences
	Ly6C	HK1.4	BioLegend
	Ly6G	1A8	BD Biosciences
	Granzyme A	GzA-3G8.5	ThermoFisher
	FoxP3	FKJ-16a	ThermoFisher
Intracellular	Ki-67	B56	BD Biosciences
	Arg1	A1exF5	ThermoFisher
	iNos	CXNFT	ThermoFisher
