# Supplementary material for: A tailored in vivo CRISPR screen identifies BAP1 as a potent tumor suppressor of sarcoma
Source: JCI Insight. 2026 Jun 30;11(16):e192686. doi: 10.1172/jci.insight.192686 (PMC13502177; doi:10.1172/jci.insight.192686)

Supplementary Figure 14. Raw images of all western blotting are presented in this manuscript.

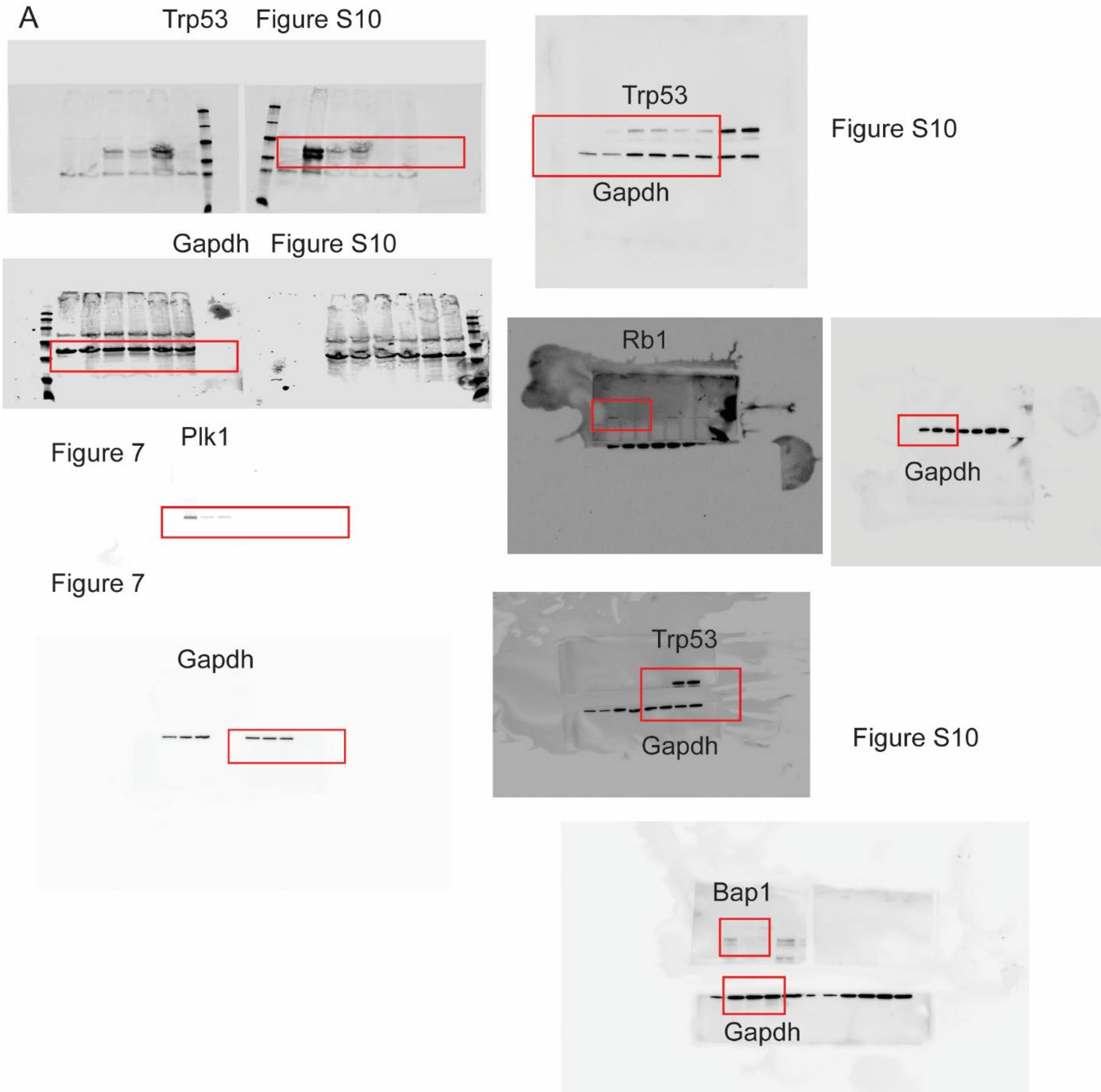

Supplement: Supplementary file 2 [file jciinsight-11-192686-s121.pdf]
